# Supplementary material for: Body composition and physical activity as mediators in the relationship between socioeconomic status and blood pressure in young South African women: a structural equation model analysis
Source: BMJ Open. 2018 Dec 19;8(12):e023404. doi: 10.1136/bmjopen-2018-023404 (PMC6303607; doi:10.1136/bmjopen-2018-023404)
Supplement: Supplementary data [file bmjopen-2018-023404supp001.pdf]

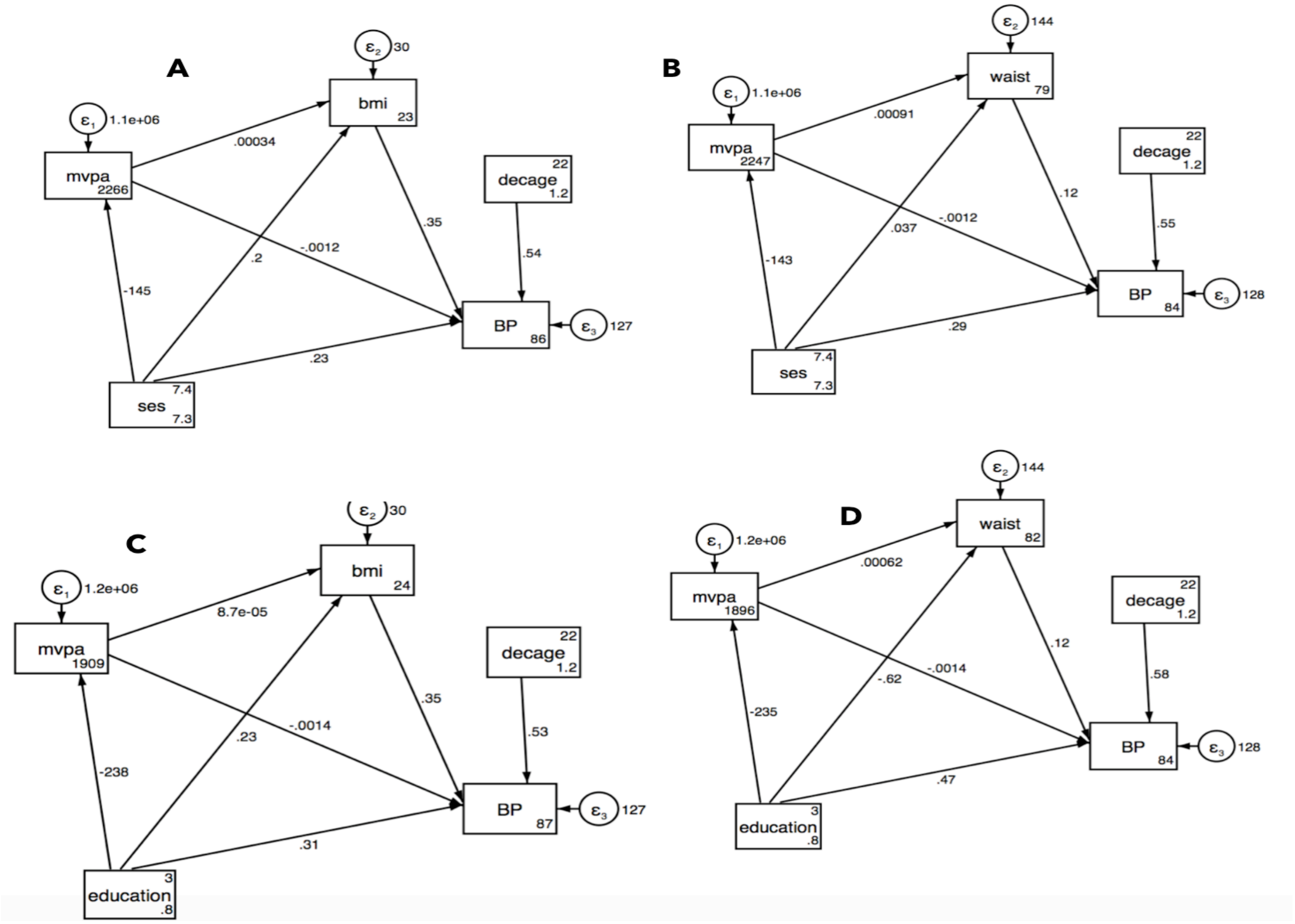

Figure SI; A: Structural equation model for **SES**, MVPA and **BMI** on SBP pooled; B: Structural equation model for **SES**, MVPA and **WC** on SBP pooled; C: Structural equation model for **education,** MVPA and **BMI** on SBP pooled; D: Structural equation model for **education,** MVPA and **WC** on SBP pooled.

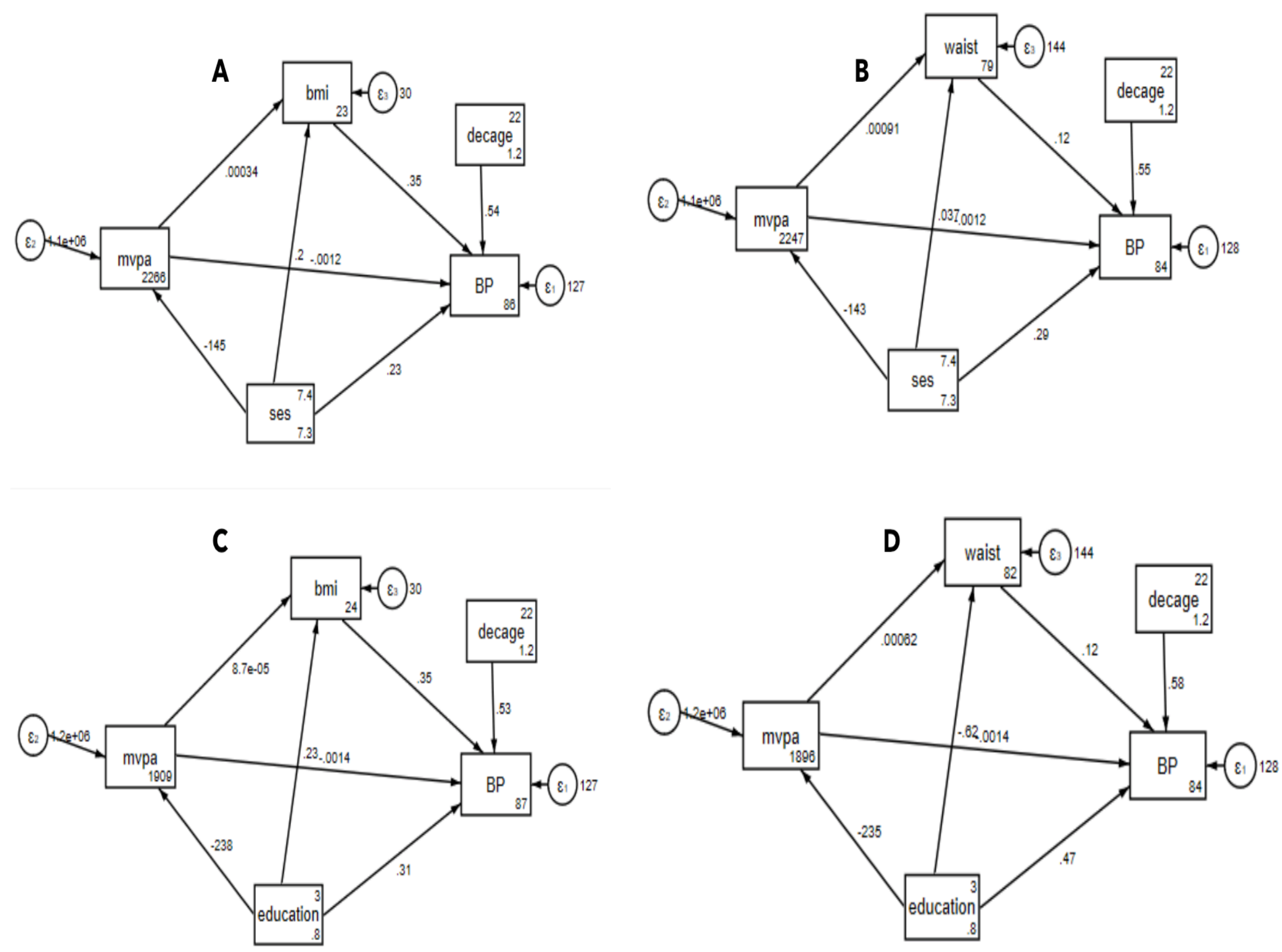

Figure S2 - SEMs based on Satorra–Bentler; A: Structural equation model for **SES**, MVPA and **BMI** on SBP pooled; B: Structural equation model for **SES**, MVPA and **WC** on SBP pooled; C: Structural equation model for **education,** MVPA and **BMI** on SBP pooled; D: Structural equation model for **education,** MVPA and **WC** on SBP pooled.
