# Supplementary material for: Body composition and physical activity as mediators in the relationship between socioeconomic status and blood pressure in young South African women: a structural equation model analysis
Source: BMJ Open. 2018 Dec 19;8(12):e023404. doi: 10.1136/bmjopen-2018-023404 (PMC6303607; doi:10.1136/bmjopen-2018-023404)
Supplement: Author's manuscript [file bmjopen-2018-023404.draft_revisions.pdf]

BMJ Open

BMJ Open is committed to open peer review. As part of this commitment we make the peer review history of every article we publish publicly available.

When an article is published we post the peer reviewers' comments and the authors' responses online. We also post the versions of the paper that were used during peer review. These are the versions that the peer review comments apply to.

The versions of the paper that follow are the versions that were submitted during the peer review process. They are not the versions of record or the final published versions. They should not be cited or distributed as the published version of this manuscript.

BMJ Open is an open access journal and the full, final, typeset and author-corrected version of record of the manuscript is available on our site with no access controls, subscription charges or pay-per-view fees (http://bmjopen.bmj.com).

If you have any questions on BMJ Open's open peer review process please email
info.bmjopen@bmj.com

BMJ Open

Body composition and physical activity as mediators in the relationship between socio-economic status and blood pressure in young South African women: A structural equation model analysis

Journal:	*BMJ Open*
Manuscript ID	bmjopen-2018-023404
Article Type:	Research
Date Submitted by the Author:	10-Apr-2018
Complete List of Authors:	Munthali, Richard; University of the Witwatersrand, MRC/Wits Developmental Pathways for Health Research Unit<br>Manyema, Mercy; Wits University, Epidemiology and Biostatistics<br>Said-Mohamed, Rihlat; University of the Witwatersrand, MRC/Wits Developmental Pathways for Health<br>Kagura, Juliana; University of Witwatersrand, Paediatrics and Child health<br>Tollman, Stephen; University of the Witwatersrand,<br>Kahn, Kathleen; University of the Witwatersrand,<br>Gómez-Olivé, F. Xavier; University of the Witwatersrand, Medical Research Council/Wits Rural Health and Health Transitions Unit (Agincourt), School of Public Health, Faculty of Health Sciences<br>Micklesfield, Lisa; University of the Witwatersrand, MRC/Wits Developmental Pathways for Health Research Unit<br>Dunger, David; University of Cambridge, Paediatrics<br>Norris, Shane; University of Witwatersrand, Paediatrics and Child Health
Keywords:	Hypertension < CARDIOLOGY, Obesity, Socioeconomic status, Physical activity, Structural equation model, Body mass index

SCHOLARONE™
Manuscripts

Body composition and physical activity as mediators in the relationship between socio-economic status and blood pressure in young South African women: A structural equation model analysis

Richard J Munthali[1], Mercy Manyema[1, 2], Rihlat Said-Mohamed[1], Juliana Kagura[1], Stephen Tollman [3,4,5], Kathleen Kahn[3, 4,5], F. Xavier Gómez-Olivé[3], Lisa K. Micklesfield[1], David Dunger [6,1], Shane A. Norris[1]

Affiliations:

[1]MRC/WITS Developmental Pathways for Health Research Unit, Department of Paediatrics, School of Clinical Medicine, Faculty of Health Sciences, University of the Witwatersrand, 7 York Rd, Parktown 2193, Johannesburg, South Africa

[2]DST-NRF Centre of Excellence in Human Development, University of the Witwatersrand, Johannesburg, South Africa

[3]MRC/Wits Rural Public Health and Health Transitions Research Unit, School of Public Health, Faculty of Health Sciences, University of the Witwatersrand, Johannesburg, South Africa

[4] INDEPTH Network, Accra, Ghana

[5]Umeå Centre for Global Health Research, Sweden.

[6]Department of Paediatrics, MRL Wellcome Trust-MRC Institute of Metabolic Science, NIHR Cambridge Comprehensive Biomedical Research Centre, University of Cambridge, Box 116, Addenbrooke's Hospital, Hills Road, Cambridge CB2 0QQ, UK

Conflict of interest: The views expressed in the submitted article are our own and not an official position of the affiliated institutions or funder. Authors have no financial relationships relevant to this article to disclose.

Corresponding Author: Dr. Richard J. Munthali

MRC/Wits Developmental Pathways for Health Research Unit

University of the Witwatersrand

Johannesburg

Tel: +27119331122

Email: munthali@aims.ac.za

For peer review only

Authors' emails:

Richard Junganiko Munthali: munthali@aims.ac.za

Mercy Manyema: mercy.manyema@gmail.com

Rihlat Said-Mohamed: rihlat.saidmohamed@wits.ac.za

Juliana Kagura: julianakagura@gmail.com

Stephen Tollman: stephen.tollman@wits.ac.za

Kathleen Kahn: kathleen.kahn@wits.ac.za

F. Xavier Gómez-Olivé: F.Gomez-OliveCasas@wits.ac.za

Lisa K. Micklesfield: lisa.micklesfield@wits.ac.za

David Dunger: dbd25@cam.ac.uk

Shane A Norris: shane.norris@wits.ac.za

For peer review only

Abstract

Objectives Varying hypertension prevalence across different socio-economic strata within a population has been well reported. However the causal factors and pathways across different settings are less clear, especially in sub-Saharan Africa. Therefore, this study aimed to compare blood pressure levels, and investigate the extent to which socioeconomic status (SES) is associated with blood pressure, in rural and urban South Africa women.

Setting Rural and urban South Africa.

Design Cross-section.

Participants Cross-sectional data on SES, total moderate-vigorous physical activity (PA), anthropometric and blood pressure data were collected on rural (n=509) and urban (n=510) young black women (18-23 years age). Pregnant and mentally or physically disabled women were excluded from the study.

Results The prevalence of combined overweight and obesity (46.5% versus 38.8%) and elevated blood pressure (27.0% versus 9.3%) were higher in urban than rural women respectively. Results from the structural equation modelling showed significant direct positive effects of BMI and SBP in rural, urban and combined datasets. Negative direct effects of SES on SBP and positive total effects of SES on SBP were observed in the rural and combined datasets respectively. In rural young women, SES had direct positive effects on BMI and was negatively associated with MVPA in urban and combined analyses. Body mass index mediated the positive total effects association between SES and SBP in combined analyses (ß; 95%CI, 0.46; 0.15 to 0.76).

Conclusions Though South Africa is undergoing nutritional and epidemiological transitions; the prevalence of elevated BP still varies between rural and urban young women. The association between socioeconomic status and SBP varies considerably in economically diverse populations with BMI being the most significant mediator. There is need to tailor prevention strategies to take account optimizing BMI when designing strategies to reduce future risk of hypertension in young women.

Keywords Blood pressure, Body mass index, Hypertension, Obesity, Urban, Rural, Socioeconomic status, Structural equation model, Physical activity

For peer review only

Strengths

1. The use of structural modelling allowed us to explore direct and indirect (mediation) effects of social economic status, physical activities and body mass index on elevated blood pressure from representative sample of rural and urban population of South African young women.
2. Although the urban and rural cohorts were from two different studies, the same research unit conducted both studies and, therefore, the methodology was harmonized between the two sites, thereby allowing for accurate comparison.

Limitations

1. Other unmeasured data, such as undernutrition in infancy, and dietary patterns were not included in the current analyses. We are currently working on research to address this limitation.
2. The low reliability of self-report data on physical activity could introduce bias. Thus, there is need for more precise, objective measures of physical activity to strengthen the results of our analysis.
3. There is need to do comparison on longitudinal data, especially as the socioeconomic environment is changing rapidly due to rural-urban labor migration and other factors would be helpful to examine these associations over time.

For peer review only

Introduction

High blood pressure (hypertension) is a leading risk factor contributing to the global disease burden, accounting for 7% of global disability-adjusted life years (DALYs) and contributing to the 34.5 million non-communicable disease (NCD) related deaths in 2010 [1, 2]. A recent global meta-analysis, involving 19.1 million individuals, reported that on average there has been a decrease in blood pressure globally, but the low- to middle-income countries (LMICs) have seen an increase in hypertension [3]. The prevalence of high blood pressure in LMICs is estimated at 30% [4, 5] and it is the most significant risk factor for cardiovascular disease, most notably stroke [6]. In 2000, hypertension was estimated to have caused 9% of all deaths and over 390 000 DALYs in South Africa. Further, hypertension contributed to 50% of all strokes and 42% of ischaemic heart disease (IHD), signifying a substantial public health burden [7]. A systematic review of sub-Saharan African (SSA) data shows prevalence rates of hypertension of up to 41% with higher prevalence rates noted in urban compared to rural populations [8, 9]. A recent study in men and women aged 40 to 60 years of age in six sites across four SSA countries, including South Africa, showed the same trend with South African urban and rural cohorts having the highest prevalence (41.6 to 54.1%) [10].

Low and middle-income countries are experiencing both epidemiological and nutritional transitions with urban populations further along the transition as demonstrated by the higher prevalence of obesity and NCDs [4, 5, 8, 10-15]. Some evidence has shown that there are differences in the levels of blood pressure between rural and urban settings [8], while other studies have found no significant differences [16]. According to Glass and McAtee, internal biological systems are sculpted by an interaction between genes and prolonged exposure to particular external environments, a principle they call embodiment [17]. Thus the differences in built and social environments between rural and urban settings may explain the differences in disease prevalence. A Ghanaian study showed that both systolic and diastolic blood pressure were significantly lower in rural participants compared to urban participants [18]. However, a similar study in adolescents found that blood pressure levels were only lower in rural boys, with no difference in the girls [19]. Pediatric and adolescent hypertension have been reported to track into adulthood in a South African urban population [20]. Results on elevated blood pressure from studies in rural South African children have reported prevalence rates varying from 1.0% to 25.4% [21-24]. The factors explaining these differences have not been fully studied in LMICs.

Socioeconomic factors such as education, household income and household assets have been associated with blood pressure levels [25-27]. In a US cohort of young adults, a higher household income remained associated with lower systolic blood pressure (SBP) even after controlling for all potential covariates including age, sex and bio-behavioral factors [28]. Similarly, in a French sample of 30-79 year olds, SBP independently increased and was inversely associated with both individual education and residential neighborhood education [29]. Studies in African countries have also found varying associations between SES and blood pressure patterns, with both positive and negative associations reported [8, 30, 31]. Some studies have speculated that the association between SES and body mass index (BMI), physical activity levels, diet, smoking, alcohol intake and malnutrition may influence blood pressure

For peer review only

patterns [18, 28, 31, 32]. Physical activity has been inversely associated with blood pressure and BMI directly associated with BP in more advanced economies, but inconsistent associations have been reported in LMICs [25, 33-37].

There is a need to examine blood pressure and its determinants in young South African adults given the high rates of overweight and obesity and hypertension observed in this age group [20, 38]. Recent South African reports also indicate that the highest pregnancy rates occur in the age range of 20-24 years, with 26.2% of births reported, followed closely by the 25-29 year age group (25.7%) [39], and therefore targeting young adult women would also reduce adverse health outcomes in their children. To better target policies or programmes in future to address hypertension and obesity in the different settings, it is important to examine more closely rural-urban differences in hypertension due to differences in the epidemiology of obesity, SES divergence in the South African context [23, 26, 30, 40-43]. Therefore, this study aims to compare blood pressure between rural and urban young adult South African women, and to determine whether there is an association between SES and blood pressure and whether it is mediated physical activity and BMI.

Methods

Study sample and site

The rural Agincourt site, 2016 potential the female participants between the ages of 18 and 23 years were in the existing Agincourt Health and Socio-demographic Surveillance System database [44]. Only 996 were located during the data collection period and were invited to participate, of these 509 female participants were recruited after giving consent to participate. The urban sample consisted of 510 young women between the ages of 21 and 24 years who were randomly selected from the sample of 720 females who were part of the Birth-to-Twenty plus (BT20+) Young Adult Survey [45, 46]. Young women (n=51) who were pregnant at the time of the study were excluded. Measurements and questionnaires were completed by trained research assistants and nurses, and were standardised between both sites, to eliminate biases. The study protocols were approved by the Human Research Ethics Committee of the University of the Witwatersrand (Clearance certificates M120138 for the Ntshembo-Hope Cross Sectional Survey in Agincourt and M111182 for the BT20+ survey). A written consent to participate was provided by participants and mentally or physically disabled women were exluded from the study.

Blood pressure

Blood pressure (mm Hg) was the outcome variable and it was measured using an Omron 6 automated machine (Kyoto, Japan). A five minute seated rest was observed before taking the blood pressure measurements. Participants' seated blood pressure was measured three times on the right side, with a 2 min interval between each measurement. The mean for the second and third readings was recorded for the current analysis.

According to the Seventh Report of the Joint National Committee on Prevention, Detection, Evaluation, and Treatment of High Blood Pressure [47], five categories of blood pressure have been established for adults 18 years

For peer review only - http://bmjopen.bmj.com/site/about/guidelines.xhtml

of age and older as shown in Table 1. These cut-offs were utilized in the current study. Prehypertension and hypertension were combined to create a new variable called elevated BP.

Table 1: Blood pressure classification [47]

Classification	Systolic Blood Pressure		Diastolic Blood Pressure
Low	<90	or	< 60
Normal	<120	and	<80
Prehypertension	120-139	or	80-90
High: Stage 1 Hypertension	140-159	or	90-99
High: Stage 2 hypertension	≥160	or	≥100

Systolic Blood Pressure was used in structural equation models (SEM) as it is more relevant in adults, and a good predictor of adverse health outcomes later in life [48], such as CVDs.

Anthropometry

At both sites, participants' height and weight were measured by trained research assistants using standard techniques [49, 50] . Weight was measured in light clothing and barefoot to the nearest 0.1 kg using a digital scale (Tanita model TBF-410; Arlinghton Heights; USA). Height was measured barefoot to the nearest 0.1 cm using a stadiometer (Holtain, Crymych, UK). Waist circumference was measured with a non-stretchable fibreglass tape at the level of the umbilicus. Body mass index (BMI) was calculated as weight/height^2 (kg/m^2).

Socio-economic status (SES)

Physical assets owned in the participants' household were used as a proxy for socio-economic status index [51]. It was generated by summing the number of assets owned in the household from the following: television, car, washing machine, fridge, phone, radio, microwave, cell phone, DVD/Video, DSTV (cable channel), computer, internet, medical aid. Previous studies in this population have shown that the sum of physical assets (household assets) is closely related to the household per capital expenditure and household income [51-53]. The household SES is regarded as a good measure of accumulated household wealth so it is a more reflective wealth index than income of a household's wealth over time.

Physical activity

For peer review only - http://bmjopen.bmj.com/site/about/guidelines.xhtml

The Global Physical Activity Questionnaire (GPAQ), developed for global physical activity surveillance, was completed via interview to obtain self-reported physical activity [54]. Total moderate-vigorous intensity physical activity (MVPA) in minutes per week (mins/wk) was calculated by adding occupation, travel-related and leisure time moderate and vigorous intensity physical activity. Sitting time (mins/wk) was used as a proxy for sedentary time.

Statistical analyses

Analysis of variance and student's t test, and Chi-squared tests and Wilcoxon rank sum test for non-parametric variables, were conducted to compare study characteristics between urban and rural young women. Structural equation modeling (SEM), with missing data option, was used to test and estimate the direct and indirect associations between variables, most especially the mediation roles of physical activity (MVPA) or sedentary time (sitting), and body composition (BMI and waist circumference), in the association between SES and blood pressure (systolic blood pressure).

Direct, indirect and total effects were computed and recorded, and the proportion of the total effect mediated was calculated. To evaluate the best fitting model for our data, we calculated different goodness of fit indices including Chi-squared test, Root mean squared error of approximation (RMSEA), Comparative fit index (CFI), Tucker-Lewis index (TLI) and Standardized root mean squared residual (SRMR) [55]. Though the Chi-squared test has been popularly used as a goodness of fit index, it has been reported to be biased and not reliable as the only goodness of fit index. It is also highly sensitive to sample size [56, 57] , and often inflated with non-normal data such as physical activity data and we therefore employed the Hu and Bentler's Two-Index Presentation Strategy (1999) combination rule, with cut off values depending on the fitness index, to determine the best model fit [55, 58].

If the direct and indirect effects had opposite signs (negative or positive effects) the proportion mediated was assessed using the absolute values for all indirect and direct effects [59]. All the analyses were conducted using STATA (version 13.0; STATA Corp., College Station, TX, USA).

Results

Study characteristics

Descriptive statistics for the non-pregnant study participants (urban, n=492; rural, n=476) are presented in Table 2. There was no difference in BMI or waist circumference between the urban and rural participants, but the prevalence of overweight and obesity was significantly higher in the urban (46.5%) compared to the rural young women (38.8%). Household SES was significantly higher in the urban compared to the rural group. Self reported physical activity (total MVPA) was significantly higher in the rural than urban women ($p<0.001$), and the urban women spent significantly more time sitting than their rural counterparts ($p<0.001$). Systolic and diastolic BP were significantly higher in the urban group, as was the prevalence of elevated BP (27.0 vs. 9.3%).

For peer review only - http://bmjopen.bmj.com/site/about/guidelines.xhtml

Structural equation models for BMI and waist circumference

Results from the SEMs for SES associations with SBP via MVPA and BMI are presented in Tables 3a, 3b and 3c for urban, rural and combined analyses respectively, and also shown in Figures 1, 2, 3. No significant direct or indirect effects via (MVPA or BMI) of SES on SBP were observed in either the urban or rural women, but there were significant direct effects of SES on MVPA. Results showed that individuals with a higher SES index were less likely to be physically active in pooled data and urban women. In rural women, a one-unit increase in total household assets was associated with a decrease of 0.65 mmHg (-1.19 to -0.10) in SBP and an increase of 0.27 kg/m^2 in BMI (0.1 to 0.53) (**Tables 3a, 3b and Figures 1, 2)**. The SEM for the combined sample showed a significant indirect effect of household SES on SBP via BMI, with 50% of the total effect being mediated by BMI **(Table 3c and Figure 3).** Direct positive effects of BMI on SBP were observed in both settings and the pooled sample with a 1 kg/m^2 increase in BMI being associated with an increase of 0.37 mmHg (0.21 to 0.53) and 0.33 (0.12 to 0.54) mmHg SBP in urban and rural young women, respectively. Similar results were observed when including waist circumference as the body composition indicator (data not shown).

Discussion

A rising prevalence of hypertension has been reported in South Africa, with Peer et al. reporting a higher prevalence in 2008 (35.6%) compared to 1990 (21.6 %) in men and women aged 25-74 years in an urban black community in Cape Town, South Africa [40]. We have shown in young adult women from urban and rural South Africa, an overall elevated BP prevalence of 18.4 % (27.0 % in urban and 9.3 % in rural). We have also shown a direct effect of BMI on SBP in the urban and rural women separately, as well as when pooled, thereby providing further evidence of an association between overall adiposity and blood pressure. The total effects of SES on SBP were the same in both settings.

Prevalence data on elevated BP and hypertension from other countries in sub-Saharan Africa have shown conflicting results when comparing urban and rural communities. In Malawi, a higher prevalence of hypertension in urban compared to rural communities has been reported and attributed to differences in lifestyle as rural communities participate in subsistence based agricultural activities while the urban community has a more westernized lifestyle with higher salt intake and physical inactivity [9]. Similarly, data from Ghana have shown a higher mean SBP and DBP and a higher prevalence of hypertension in urban communities [18, 60]. In the PURE study in South Africa, Pisa and colleagues reported that both urban adult men and women had higher mean blood pressures in comparison to their rural peers though the overall CVD risk factors were equally prevalent in both settings [41]. In contrast, findings from Cameroon have reported a higher BP prevalence in rural compared to urban men and women older than 40 years old, while Kenyan studies have reported no significant differences [16, 61]. Results from six urban and rural sites in four sub-Saharan African countries – Kenya, South Africa, Ghana and Burkina Faso – have reported a prevalence of hypertension in women aged between 40 and 60 years ranging from 15.1% in rural Burkina Faso to 54.1% in urban South Africa [10]. It was also reported that in all three South African sites, both rural and urban, the

For peer review only

prevalence of hypertension was higher than in the other three countries [10]. These findings show the complex health transitions occurring in SSA and the impact that this is having on cardio-metabolic disease risk.

Our study showed significant differences in SES between the urban and rural samples, as well a big variation in SES between these two settings. The social patterning of CVD risk factors, including hypertension, in SSA and LMICs has in part been attributed to differences in countries' socioeconomic development. Previous results from five countries, (two high income and three LMICs), reported that hypertension and other CVD risk factors were substantially associated with education and wealth status; individuals with less education and lower wealth generally showing higher prevalence of CVD risk factors [62] . The effect of SES in this study is most evident in the rural women for whom household SES was lower (compared to urban) and who may be transitioning faster (both nutritionally and economically) than the urban women. Though SES is positively associated with BMI in rural young women, it is negatively associated with SBP. There may be other factors, such as physical activity due to agricultural activities or dietary patterns, which were not recorded. In addition, the weight gain observed might not be due to fat mass but rather to muscle mass and bone mass, which has been reported to be associated with SBP before [63].

In Mexico, women in rural and upper SES categories were likely to have a higher SBP, while we have reported that a higher SES was associated with a decrease in SBP in rural communities. At population level, there is a need to consider different SES categories and monitor the effect of transitioning from one category to another on hypertension, since these categories may respond differently to an increase or a decrease in their SES. Kagura and colleagues tracked SES in South African children and reported that moving from the low SES in infancy to a higher SES in adolescence had a protective effect on SBP level in young adulthood [26]. Our results have shown that this could be more pronounced in rural areas.

We observed a positive association between SES and BMI in the rural sample and the same direction of effects was observed in the urban (though not significant), which is in line with results reported in many LMICs including South Africa, but in contrast with those reported in higher income populations [33, 34, 62]. A systematic review of studies between 1989 to 2007 reported that SES was positively associated with obesity in the middle transitioning economies such as South Africa and Jamaica [64]. We have shown that both in the rural and urban participants (not significant), a higher SES resulted in reduced SBP, while the pooled (combined) analysis showed a positive total effect association between SES and SBP. This could be due to the introduction of more variation in SES when data from both sites are combined; with many individuals with low SES in the rural area, the associations became skewed towards the low SES individuals. This may suggest that different transitional levels of SES have different effects on hypertension risk depending on the environment (either urban or rural). Though not significant, the total effects of SES on SBP are the same in both rural and urban hence the differences in prevalence cannot be explained by the setting or SES alone. In urban and rural settings of four countries (Kenya, Namibia, Nigeria and Tanzania), the prevalence of age standardized hypertension was similarly high and ranging from 19.3 % to 38.0 % [11]. Cois and colleagues reported that a higher SES was associated with lower SBP in a nationally representative sample of South African women [25] using SEM models. Physical exercise, alcohol use, smoking and resting heart rate and BMI

For peer review only - http://bmjopen.bmj.com/site/about/guidelines.xhtml

were reported to be the mediators of the indirect of the association between SES and SBP in men but not in women, suggesting that other factors may play a major role in women [25]. Similarly, our results show that neither PA nor BMI mediate the association between SES and SBP in urban and rural settings, suggesting that other factors may explain the association. Among those, dietary patterns and stress have been reported to be independently associated with SBP [65, 66].

The significant direct associations between BMI and SBP or hypertension are in line with other findings in South Africa and within the SSA region [11, 33, 40, 42, 67, 68]. This link was consistent in rural, urban and combined data sets, indicating the importance of BMI in the aetiology of blood pressure. Munthali et al reported that the link between obesity and hypertension could be observed as early as five years of age. Children with early onset of obesity were at higher risk of developing hypertension in late adolescence [38].

In this study, using SEM models to explore the mediation role of BMI and PA helped quantify potential contributions of these variables to the effect of SES on SBP. The results show that PA was not a significant mediator in the association between SES and BP in the urban or the rural samples. SES was negatively associated with MVPA in urban and pooled samples, indicating that as individuals transition from low to higher SES, they reduce their physical activity level. We speculate that these differences in the association between SES and SBP in both our rural and urban results and in those from high-income countries are due to differences in levels of nutritional and epidemiological transition in these regions [69, 70]. Those with low SES in high-income countries are likely to consume cheaper, more energy dense foods, participate in less leisure time physical activity and be more sedentary [71, 72] In LMICs, agricultural activities remain a part of everyday life and a day-to-day activity in rural living, while those with higher SES in the same settings rapidly adopt the westernized life style with less PA, fewer agricultural activities and home grown food. However, this speculation is not supported by the data on PA in this study despite the rural participants having a higher PA. Our understanding of the Agincourt rural economy is that agriculture is quite a minor aspect though very useful to augment the household income.

The limitations of this study are that other unmeasured data, such as undernutrition in infancy, which is a known risk factor for high blood pressure later in life [73], and dietary patterns were not included in the current analyses. We are currently working on research to address this limitation. We can also not rule out the role of genetics. Secondly, the low reliability of self-report data on physical activity could introduce bias. Thus, there is need for more precise, objective measures of physical activity to strengthen the results of our analysis. Lastly, longitudinal data, especially as the socioeconomic environment is changing rapidly due to rural-urban labor migration and other factors would be helpful to examine these associations over time.

Conclusions

Though the prevalence of overweight or obesity is relatively higher in both rural and urban than those reported in other SSA countries, women in the urban setting were at more risk for elevated blood pressure than their rural counterparts. The link between socioeconomic status and SBP varies in a more economically diverse population, as

For peer review only

For peer review only - http://bmjopen.bmj.com/site/about/guidelines.xhtml

seen with the combined rural and urban dataset, with BMI being the most likely mediator. There is need to consider optimizing BMI as a key intervention strategy in young adults in part to combat hypertension.

Funding

Norris is supported by the UK MRC DfID African Research Leader Scheme and by the DST-NRF Centre of Excellence in Human Development at the University of the Witwatersrand, Johannesburg. Birth to Twenty data collection was supported by the Wellcome Trust under Grant [092097/Z/10/Z]. The MRC/Wits- Agincourt Unit is supported by the South African Medical Research Council, and the Wellcome Trust under Grants [058893/Z/99/A, 069683/Z/02/Z, 085477/Z/08/Z, 085477/B/08/Z]. Opinions expressed and conclusions arrived at, are those of the authors and are not to be attributed to the CoE in Human Development.

Conflict of interest

Authors have no conflicts of interest to disclose.

Consent for publication

Not applicable

Availability of data and material

The datasets used and/or analysed during the current study are available from the Developmental Pathways for Health Research Unit data management department by contacting Prof. Shane A Norris on reasonable request

Competing interest

The authors declare that they have no competing interests

Authors' contributions

RJM and SAN conceptualized the manuscript. RJM analyzed the data. RJM MM RSM JK ST KK FXG LKM DD SAN interpreted the data. RJM wrote the manuscript and all authors were involved in editing and approving the final manuscript.

Acknowledgements

We wish to thank the Bt20+ and Agincourt participants for taking part in the study and the Bt20+ and Agincourt team for their relentless support throughout the study.

Ethics Approval and Consent to Participate

Prior to the study, the study protocols were approved by the Human Research Ethics Committee of the University of the Witwatersrand (Clearance certificates M120138 for the Ntshembo-Hope Cross Sectional Survey in Agincourt and M111182 for the BT20+ survey). Independent written informed consent to participate was obtained from participants.

For peer review only

References

1. Lim SS, Vos T, Flaxman AD, Danaei G, Shibuya K, Adair-Rohani H, AlMazroa MA, Amann M, Anderson HR, Andrews KG *et al*: **A comparative risk assessment of burden of disease and injury attributable to 67 risk factors and risk factor clusters in 21 regions, 1990–2010: a systematic analysis for the Global Burden of Disease Study 2010**. *The Lancet* 2012, **380**(9859):2224-2260.
2. Lozano R, Naghavi M, Foreman K, Lim S, Shibuya K, Aboyans V, Abraham J, Adair T, Aggarwal R, Ahn SY: **Global and regional mortality from 235 causes of death for 20 age groups in 1990 and 2010: a systematic analysis for the Global Burden of Disease Study 2010**. *The Lancet* 2013, **380**(9859):2095-2128.
3. Ezzati M, Geleijnse J: **Worldwide trends in blood pressure from 1975 to 2015**. *The Lancet* 2017, **389**(10064):37-55.
4. Sarki AM, Nduka CU, Stranges S, Kandala N-B, Uthman OA: **Prevalence of Hypertension in Low- and Middle-Income Countries: A Systematic Review and Meta-Analysis**. *Medicine* 2015, **94**(50):e1959.
5. Chow CK, Teo KK, Rangarajan S, Islam S, Gupta R, Avezum A, Bahonar A, Chifamba J, Dagenais G, Diaz R: **Prevalence, awareness, treatment, and control of hypertension in rural and urban communities in high-, middle-, and low-income countries**. *Jama* 2013, **310**(9):959-968.
6. Kearney PM, Whelton M, Reynolds K, Muntner P, Whelton PK, He J: **Global burden of hypertension: analysis of worldwide data**. *The lancet* 2005, **365**(9455):217-223.
7. Norman R, Gaziano T, Laubscher R, Steyn K, Bradshaw D, Collaboration SACRA: **Estimating the burden of disease attributable to high blood pressure in South Africa in 2000**. *South African Medical Journal* 2007, **97**(8):692-698.
8. Addo J, Smeeth L, Leon DA: **Hypertension in Sub-Saharan Africa a systematic review**. *Hypertension* 2007, **50**(6):1012-1018.
9. Simmons D, Barbour G, Congleton J, Levy J, Meacher P, Saul H, Sowerby T: **Blood pressure and salt intake in Malawi: an urban rural study**. *Journal of epidemiology and community health* 1986, **40**(2):188-192.
10. Gómez-Olivé FX, Ali SA, Made F, Kyobutungi C, Nonterah E, Micklesfield L, Alberts M, Boua R, Hazelhurst S, Debpuur C: **Stark Regional and Sex Differences in the Prevalence and Awareness of Hypertension: An H3Africa AWI-Gen Study Across 6 Sites in Sub-Saharan Africa**. *Global Heart* 2017.
11. Hendriks ME, Wit FW, Roos MT, Brewster LM, Akande TM, de Beer IH, Mfinanga SG, Kahwa AM, Gatongi P, Van Rooy G: **Hypertension in sub-Saharan Africa: cross-sectional surveys in four rural and urban communities**. *PloS one* 2012, **7**(3):e32638.
12. Lemogoum D, Seedat YK, Mabadeje AF, Mendis S, Bovet P, Onwubere B: **International Forum for Hypertension control and prevention in Africa. Recommendations for prevention, diagnosis and**

For peer review only

management of hypertension and cardiovascular risk factors in sub-Saharan Africa. *J Hypertens* 2003, **21**.

13. Schutte A, Botha S, Fourie C, Gafane-Matemane L, Kruger R, Lammertyn L, Malan L, Mels C, Schutte R, Smith W: **Recent advances in understanding hypertension development in sub-Saharan Africa**. *Journal of Human Hypertension* 2017.
14. Thorogood M, Connor MD, Lewando Hundt G, Tollman SM: **Understanding and managing hypertension in an African sub-district: A multidisciplinary approach1**. *Scandinavian Journal of Public Health* 2007, **35**(69 suppl):52-59.
15. Yusufali A, Khatib R, Islam S, AlHabib K, Kelishadi R, Rangarajan S, Yusuf S: **LBOS 03-01 PREVALENCE, AWARENESS, TREATMENT, AND CONTROL OF HYPERTENSION IN THE MIDDLE EAST: RESULTS FROM THE PROSPECTIVE URBAN RURAL EPIDEMIOLOGY (PURE) STUDY**. *J Hypertens* 2016, **34 Suppl 1**:e551.
16. Poulter N, Khaw K, Hopwood B, Mugambi M, Peart W, Rose G, Sever P: **Blood pressure and its correlates in an African tribe in urban and rural environments**. *Journal of epidemiology and community health* 1984, **38**(3):181-185.
17. Glass TA, McAtee MJ: **Behavioral science at the crossroads in public health: extending horizons, envisioning the future**. *Social science & medicine* 2006, **62**(7):1650-1671.
18. Agyemang C: **Rural and urban differences in blood pressure and hypertension in Ghana, West Africa**. *Public Health* 2006, **120**.
19. Agyemang C, Redekop WK, Owusu-Dabo E, Bruijnzeels MA: **Blood pressure patterns in rural, semi-urban and urban children in the Ashanti region on Ghana, West Africa**. *BMC Public Health* 2005, **5**.
20. Kagura J, Adair LS, Musa MG, Pettifor JM, Norris SA: **Blood pressure tracking in urban black South African children: birth to twenty cohort**. *BMC pediatrics* 2015, **15**(1):1.
21. Goon D, Amusa L, Mhlongo D, Khoza L, Any-Anwu F: **Elevated blood pressure among Rural South African Children in Thohoyandou, South Africa**. *Iran J Pub Health* 2013, **42**.
22. Kemp C, Pienaar AE, Schutte AE: **The prevalence of hypertension and the relationship with body composition in Grade 1 learners in the North West Province of South Africa**. *South African Journal of Sports Medicine* 2011, **23**(4).
23. Monyeki K, Kemper H, Makgae P: **The association of fat patterning with blood pressure in rural South African children: the Ellisras Longitudinal Growth and Health Study**. *International journal of epidemiology* 2006, **35**(1):114-120.
24. Schutte A, Van Rooyen J, Huisman H, Kruger H, Malan N, De Ridder J: **Dietary risk markers that contribute to the aetiology of hypertension in black South African children: the THUSA BANA study**. *Journal of human hypertension* 2003, **17**(1):29-35.
25. Cois A, Ehrlich R: **Analysing the socioeconomic determinants of hypertension in South Africa: a structural equation modelling approach**. *BMC public health* 2014, **14**(1):414.

For peer review only

26. Kagura J, Adair LS, Pisa PT, Griffiths PL, Pettifor JM, Norris SA: **Association of socioeconomic status change between infancy and adolescence, and blood pressure, in South African young adults: Birth to Twenty Cohort**. *BMJ open* 2016, **6**(3):e008805.

27. Wilson DK, Kliewer W, Plybon L, Sica DA: **Socioeconomic status and blood pressure reactivity in healthy black adolescents**. *Hypertension* 2000, **35**(1):496-500.

28. Brummett BH, Babyak MA, Siegler IC, Shanahan M, Harris KM, Elder GH, Williams RB: **Systolic Blood Pressure, Socioeconomic Status, and Biobehavioral Risk Factors in a Nationally Representative US Young Adult Sample**. *Hypertension* 2011, **58**(2):161-166.

29. Chaix B, Bean K, Leal C, Thomas F, Havard S, Evans D, Jégo B, Pannier B: **Individual/Neighborhood Social Factors and Blood Pressure in the RECORD Cohort Study**. *Which Risk Factors Explain the Associations?* 2010, **55**(3):769-775.

30. Griffiths PL, Sheppard ZA, Johnson W, Cameron N, Pettifor JM, Norris SA: **Associations between household and neighbourhood socioeconomic status and systolic blood pressure among urban South African adolescents**. *Journal of biosocial science* 2012, **44**(04):433-458.

31. Longo-Mbenza B, Luila EL, M'Buyamba-Kabangu J: **Nutritional status, socio-economic status, heart rate, and blood pressure in African school children and adolescents**. *International journal of cardiology* 2007, **121**(2):171-177.

32. Aounallah-Skhiri H, El Ati J, Traissac P, Romdhane HB, Eymard-Duvernay S, Delpeuch F, Achour N, Maire B: **Blood pressure and associated factors in a North African adolescent population. A national cross-sectional study in Tunisia**. *BMC Public Health* 2012, **12**(1):1.

33. Afrifa–Anane E, Agyemang C, Codjoe SNA, Ogedegbe G, Aikins Ad-G: **The association of physical activity, body mass index and the blood pressure levels among urban poor youth in Accra, Ghana**. *BMC public health* 2015, **15**(1):269.

34. Anane AE, Agyemang C, Codjoe SNA, Ogedegbe G, Aikins A: **The association of physical activity, Body mass index and blood pressure among urban poor youth in Accra, Ghana**. *BMC Public Health* 2015, **15**.

35. Hu G, Barengo NC, Tuomilehto J, Lakka TA, Nissinen A, Jousilahti P: **Relationship of physical activity and body mass index to the risk of hypertension: a prospective study in Finland**. *Hypertension* 2004, **43**(1):25-30.

36. Juraschek SP, Blaha MJ, Whelton SP, Blumenthal R, Jones SR, Keteyian SJ, Schairer J, Brawner CA, Al-Mallah MH: **Physical fitness and hypertension in a population at risk for cardiovascular disease: the Henry Ford ExercIse Testing (FIT) Project**. *Journal of the American Heart Association* 2014, **3**(6):e001268.

37. Väistö J, Eloranta A-M, Viitasalo A, Tompuri T, Lintu N, Karjalainen P, Lampinen E-K, Ågren J, Laaksonen DE, Lakka H-M: **Physical activity and sedentary behaviour in relation to cardiometabolic risk in children: cross-sectional findings from the Physical Activity and Nutrition in Children (PANIC) Study**. *International Journal of Behavioral Nutrition and Physical Activity* 2014, **11**(1):55.

For peer review only - http://bmjopen.bmj.com/site/about/guidelines.xhtml

38. Munthali RJ, Kagura J, Lombard Z, Norris SA: **Childhood adiposity trajectories are associated with late adolescent blood pressure: birth to twenty cohort**. *BMC Public Health* 2016, **16**(1):1-10.
39. STATSSA: **Mortality and causes of death in South Africa, 2015: Findings from death notification**. In. Pretoria: Statistics South Africa; 2017 1–140. .
40. Peer N, Steyn K, Lombard C, Gwebushe N, Levitt N: **A high burden of hypertension in the urban black population of Cape Town: The Cardiovascular Risk in Black South Africans (CRIBSA) Study**. *PLoS One* 2013, **8**(11):e78567.
41. Pisa P, Behanan R, Vorster H, Kruger A: **Social drift of cardiovascular disease risk factors in Africans from the North West Province of South Africa: the PURE study: cardiovascular topics**. *Cardiovascular journal of Africa* 2012, **23**(7):371-388.
42. Steyn K, Fourie J, Lombard C, Katzenellenbogen J, Bourne L, Jooste P: **Hypertension in the black community of the Cape Peninsula, South Africa**. *East African medical journal* 1996, **73**(11):758-763.
43. Tibazarwa K, Ntyintyane L, Sliwa K, Gerntholtz T, Carrington M, Wilkinson D, Stewart S: **A time bomb of cardiovascular risk factors in South Africa: results from the Heart of Soweto Study "Heart Awareness Days"**. *International journal of cardiology* 2009, **132**(2):233-239.
44. Kahn K, Collinson MA, Gómez-Olivé FX, Mokoena O, Twine R, Mee P, Afolabi SA, Clark BD, Kabudula CW, Khosa A: **Profile: Agincourt health and socio-demographic surveillance system**. *International journal of epidemiology* 2012, **41**(4):988-1001.
45. Pradeilles R, Rousham EK, Norris SA, Griffiths PL: **Urban South African adolescents' perceptions of their neighborhood socio-economic environments: the Birth to Twenty plus cohort study**. *Children Youth and Environments* 2014, **24**(3):173-200.
46. Richter L, Norris S, Pettifor J, Yach D, Cameron N: **Cohort profile: Mandela's children: the 1990 Birth to Twenty study in South Africa**. *International journal of epidemiology* 2007, **36**(3):504-511.
47. Chobanian AV: **Joint National Committee on Prevention, Detection, Evaluation, and Treatment of High Blood Pressure. National Heart, Lung, and Blood Institute; National High Blood Pressure Education Program Coordinating Committee: Seventh report of the Joint National Committee on Prevention, Detection, Evaluation, and Treatment of High Blood Pressure**. *Hypertension* 2003, **42**:1206-1252.
48. Strandberg TE, Pitkala K: **What is the most important component of blood pressure: systolic, diastolic or pulse pressure?** *Current opinion in nephrology and hypertension* 2003, **12**(3):293-297.
49. Cameron N: **The measurement of human growth**: Taylor & Francis; 1984.
50. Lohman TG, Roche AF, Martorell R: **Anthropometric standardization reference manual**: Human kinetics books; 1988.
51. Houweling TA, Kunst AE, Mackenbach JP: **Measuring health inequality among children in developing countries: does the choice of the indicator of economic status matter?** *International journal for equity in health* 2003, **2**(1):8.

For peer review only

52. Feeley AB, Musenge E, Pettifor JM, Norris SA: **Investigation into longitudinal dietary behaviours and household socio-economic indicators and their association with BMI Z-score and fat mass in South African adolescents: the Birth to Twenty (Bt20) cohort**. *Public health nutrition* 2013, **16**(04):693-703.

53. Jones LL, Griffiths PL, Adair LS, Norris SA, Richter LM, Cameron N: **A comparison of the socio-economic determinants of growth retardation in South African and Filipino infants**. *Public health nutrition* 2008, **11**(12):1220-1228.

54. Bull FC, Maslin TS, Armstrong T: **Global physical activity questionnaire (GPAQ): nine country reliability and validity study**. *Journal of Physical Activity and health* 2009, **6**(6):790-804.

55. Hu Lt, Bentler PM: **Cutoff criteria for fit indexes in covariance structure analysis: Conventional criteria versus new alternatives**. *Structural equation modeling: a multidisciplinary journal* 1999, **6**(1):1-55.

56. Schermelleh-Engel K, Moosbrugger H, Müller H: **Evaluating the fit of structural equation models: Tests of significance and descriptive goodness-of-fit measures**. *Methods of psychological research online* 2003, **8**(2):23-74.

57. Vandenberg RJ: **Introduction: Statistical and Methodological Myths and Urban Legends: Where, Pray Tell, Did They Get This Idea?** In.: Sage Publications Sage CA: Thousand Oaks, CA; 2006.

58. Fan X, Sivo SA: **Sensitivity of fit indexes to misspecified structural or measurement model components: Rationale of two-index strategy revisited**. *Structural Equation Modeling* 2005, **12**(3):343-367.

59. Alwin DF, Hauser RM: **The decomposition of effects in path analysis**. *American sociological review* 1975:37-47.

60. Kerry SM, Emmett L, Micah FB, Martin-Peprah R, Antwi S, Phillips RO, Plange-Rhule J, Eastwood JB, Cappuccio FP: **Rural and semi-urban differences in salt intake, and its dietary sources, in Ashanti, West Africa**. *Ethnicity & disease* 2005, **15**(1):33-39.

61. Lissock CNAA, Sobngwi E, Ngassam E, Ngoa LSE: **Rural and urban differences in metabolic profiles in a Cameroonian population**. *Pan African Medical Journal* 2011, **10**.

62. Stringhini S, Forrester TE, Plange-Rhule J, Lambert EV, Viswanathan B, Riesen W, Korte W, Levitt N, Tong L, Dugas LR: **The social patterning of risk factors for noncommunicable diseases in five countries: evidence from the modeling the epidemiologic transition study (METS)**. *BMC Public Health* 2016, **16**(1):956.

63. de Hoog ML, van Eijsden M, Stronks K, Gemke RJ, Vrijkotte TG: **Association between body size and blood pressure in children from different ethnic origins**. *Cardiovascular diabetology* 2012, **11**(1):136.

64. Dinsa G, Goryakin Y, Fumagalli E, Suhrcke M: **Obesity and socioeconomic status in developing countries: a systematic review**. *Obesity reviews* 2012, **13**(11):1067-1079.

65. Masala G, Bendinelli B, Versari D, Saieva C, Ceroti M, Santagiuliana F, Caini S, Salvini S, Sera F, Taddei S: **Anthropometric and dietary determinants of blood pressure in over 7000 Mediterranean women:**

For peer review only

the European Prospective Investigation into Cancer and Nutrition-Florence cohort. *Journal of hypertension* 2008, **26**(11):2112-2120.

66. Wilson BL, Albright GL, Steiner SS, Andreassi JL: **Cardiodynamic response to psychological and cold pressor stress: further evidence for stimulus response specificity and directional fractionation**. *Biofeedback and Self-regulation* 1991, **16**(1):45-53.

67. Goon D, Amusa L, Mhlongo D, Khoza L, Any-Anwu F: **Elevated blood pressure among rural South African children in Thohoyandou, South Africa**. *Iranian journal of public health* 2013, **42**(5):489.

68. Moselakgomo VK, Toriola AL, Shaw BS, Goon DT, Akinyemi O: **Body mass index, overweight, and blood pressure among adolescent schoolchildren in Limpopo province, South Africa**. *Revista Paulista de Pediatria* 2012, **30**(4):562-569.

69. Lynch JW, Smith GD, Kaplan GA, House JS: **Income inequality and mortality: importance to health of individual income, psychosocial environment, or material conditions**. *BMJ: British Medical Journal* 2000, **320**(7243):1200.

70. Ploubidis GB, Mathenge W, De Stavola B, Grundy E, Foster A, Kuper H: **Socioeconomic position and later life prevalence of hypertension, diabetes and visual impairment in Nakuru, Kenya**. *International journal of public health* 2013, **58**(1):133-141.

71. Drewnowski A: **The cost of US foods as related to their nutritive value**. *The American journal of clinical nutrition* 2010, **92**(5):1181-1188.

72. Popkin BM: **The nutrition transition and obesity in the developing world**. *The Journal of nutrition* 2001, **131**(3):871S-873S.

73. Sawaya AL, Sesso R, Florencio TM, Fernandes MT, Martins PA: **Association between chronic undernutrition and hypertension**. *Maternal & child nutrition* 2005, **1**(3):155-163.

For peer review only

For peer review only - http://bmjopen.bmj.com/site/about/guidelines.xhtml

Table 2: Descriptive characteristics

	Total	n	Urban	n	Rural	p value
Age (years)	22.04 (1.24)	492	22.77 (0.49)	476	21.28 (1.31)	0.001
Weight (kg)	64.62 (14.82)	492	64.67 (15.6)	473	64.55 (14.03)	0.90
Height (m)	1.61 (0.007)	492	1.60 (0.07)	475	1.61 (0.07)	0.001
BMI (kg/m^2)	25.05 (5.59)	492	25.32 (5.91)	473	24.78 (5.24)	0.13
BMI classification (%)						0.015
Underweight (<18.4 kg/m^2)	5.98		7.10		4.82	
Normal weight (18.5-24.9 kg/m^2)	51.34		46.45		56.39	
Overweight (25-29.9 kg/m^2)	26.19		29.21		23.06	
Obese (>=30 kg/m^2)	16.49		17.24		15.72	
Waist circumference (cm)	80.60 (12.08)	493	80.18 (12.63)	477	81.03 (11.47)	0.26
Household SES index (sum of assets)	7.24 (2.70)	493	8.83 (2.37)	476	5.59 (1.91)	0.0000
Total MVPA (min/week)*	870(280-1810)	492	420(160-900)	385	1680(970-2580)	<0.001
Sitting time (mins/day)*	300 (240-480)	492	360 (240-480)	385	300 (180-360)	<0.001
Systolic blood pressure	106.68 (11.64)	492	110.30 (11.4)	471	102.89 (10.7)	0.000
Diastolic blood pressure	70.23 (9.00)	492	72.78 (8.3)	471	67.57 (9.0)	0.000
BP classification (%)						0.000
Low BP	12.46		5.49		19.75	
Normal BP	69.16		67.48		70.91	
Prehypertension	16.20		23.58		8.49	
Hypertensive	2.18		3.46		0.85	
Elevated BP (%)	18.38		27.04		9.34	0.000

Data presented as mean (SD) otherwise stated

* Median(IQR)

For peer review only

Table 3a: Structural equation model for SES, MVPA and BMI on SBP in urban women

**Effect of: N=489**	**Outcome:**	**Direct effects(95% CI)**	**Indirect effects(95% CI)**	**Total effects(95% CI)**	**Proportion of total effect mediated**
**Household assets**	SBP	-0.34 (-0.75; 0.07)		-0.29 (-0.70; 0.12)	0.13 [a]
	via BMI		0.05 (-0.05; 0.14)		
	BMI	0.13 (-0.09; 0.35)		0.11 (-0.11; 0.33)	0.1 [a]
	via MVPA		-0.014 (-0.05; 0.013)		
	MVPA	-41.71 (-73.48; -9.94)**		-41.71 (-73.48; -9.94)**	
**MVPA**	SBP	-0.0002 (-0.001; 0.001)		-0.0000 (-0.0012; 0.0011)	0.3 [a]
	via BMI		0.0001 (-0.0001; 0.0004)		
**BMI**	SBP	0.37 (0.21; 0.53)***		0.37 (0.21; 0.53)***	

Adjusted for age; * P<0.05; ** P< 0.01; ***P< 0.001; SBP; systolic blood pressure, MVPA; moderate to vigorous intensity physical activity, BMI; body mass index, SES; social economic status, [a] Assessed using the absolute values for both indirect and direct effects

Urban Fit Indices: LR test of model vs. saturated: chi2(4) = 0.97, Prob > chi2 = 0.91; RMSEA = 0.00**;** **CFI= 1.00** Comparative fit index; TLI= 1.12 Tucker-Lewis index; **SRMR=0.011:** Standardized root mean squared residual, CD= 0.017 Coefficient of determination.

For peer review only - http://bmjopen.bmj.com/site/about/guidelines.xhtml

Table 3b: Structural equation model for SES, MVPA and BMI on SBP in rural women

**Effect of:**<br>**N= 378**	**Outcome:**	**Direct effects(95% CI)**	**Indirect effects(95% CI)**	**Total effects(95% CI)**	**Proportion of total effect mediated**
**Household assets**	SBP<br>via BMI	-0.65 (-1.19; -0.096)*	0.08 (-0.04; 0.19)	-0.56 (-1.12; -0.02)*	0.11 [a]
	BMI<br>via MVPA	0.27 (0.01; 0.53)*	-0.01 (-0.04; 0.01)	0.26 (-0.005; 0.53)*	0.04
	MVPA	-29.51 (-87.81; 28.78)		-29.51 (-87.81; 28.78)	
**MVPA**	SBP<br>via BMI	0.0004 (-.0005729 .0013)	0.0001 (-0.0000; 0.0003)	0.0005 (-0.0005; 0.0015)	0.2
**BMI**	SBP	0.33 (0.12; 0.54)**		0.33 (0.12; 0.54)**	

Adjusted for age; * P<0.05; ** P< 0.01; ***P< 0.001; SBP; systolic blood pressure, MVPA; moderate to vigorous physical activity, BMI; body mass index, SES; social economic status, [a] Assessed using the absolute values for both indirect and direct effects

Rural Fit Indices: LR test of model vs. saturated: chi2(4) = 10.51, Prob > chi2 = 0.03; RMSEA = 0.066**; CFI= 0.72** Comparative fit index; TLI= 0.37 Tucker-Lewis index; **SRMR= 0.04 :** Standardized root mean squared residual, CD= 0.03 Coefficient of determination.

For peer review only - http://bmjopen.bmj.com/site/about/guidelines.xhtml

Table 3c: Structural equation model for SES, MVPA and BMI on SBP in the pooled sample of urban and rural women

**Effect of: N=867**	**Outcome:**	**Direct effects(95% CI)**	**Indirect effects(95% CI)**	**Total effects(95% CI)**	**Proportion of total effect mediated**
**Household assets**	SBP	0.23 (-0.08; 0.54)		0.46 (0.15; 0.76)**	0.5
	via BMI		0.23 (0.10; 0.35)***		
	BMI	0.20 (0.05; 0.34)**		0.15 (0.01; 0.29)*	0.25 [a]
	via MVPA		-0.05 (-.100; 0.003)		
	MVPA	-144.83 (-170.55; -119.12)***		-144.83 (-170.55; -119.12)***	
**MVPA**	SBP	-0.001 (-0.002; -0.0005)**		-0.001 (-0.002; -0.0003)**	0.1 [a]
	via BMI		0.0001 (-0.0000; 0.0002)		
**BMI**	SBP	0.35 (0.21; 0.49)***		0.35 (0.21; 0.49)***	

Adjusted for age; * P<0.05; ** P< 0.01; ***P< 0.001; SBP; systolic blood pressure, MVPA; moderate to vigorous intensity physical activity, BMI; body mass index, SES; social economic status, [a] Assessed using the absolute values for both indirect and direct effects

Pooled Fit Indices: LR test of model vs. saturated: chi2(4) = 24.829, Prob > chi2 = 0.000; RMSEA = 0.077**; CFI= 0.89** Comparative fit index; TLI= 0.75 Tucker-Lewis index; **SRMR=0.033:** Standardized root mean squared residual, CD=0.137 Coefficient of determination.

For peer review only - http://bmjopen.bmj.com/site/about/guidelines.xhtml

Figure legends

Figure 1: Structural equation model for SES, MVPA and BMI on SBP in urban

Figure 2: Structural equation model for SES, MVPA and BMI on SBP in rural

Figure 3: Structural equation model for SES, MVPA and BMI on SBP pooled

For peer review only

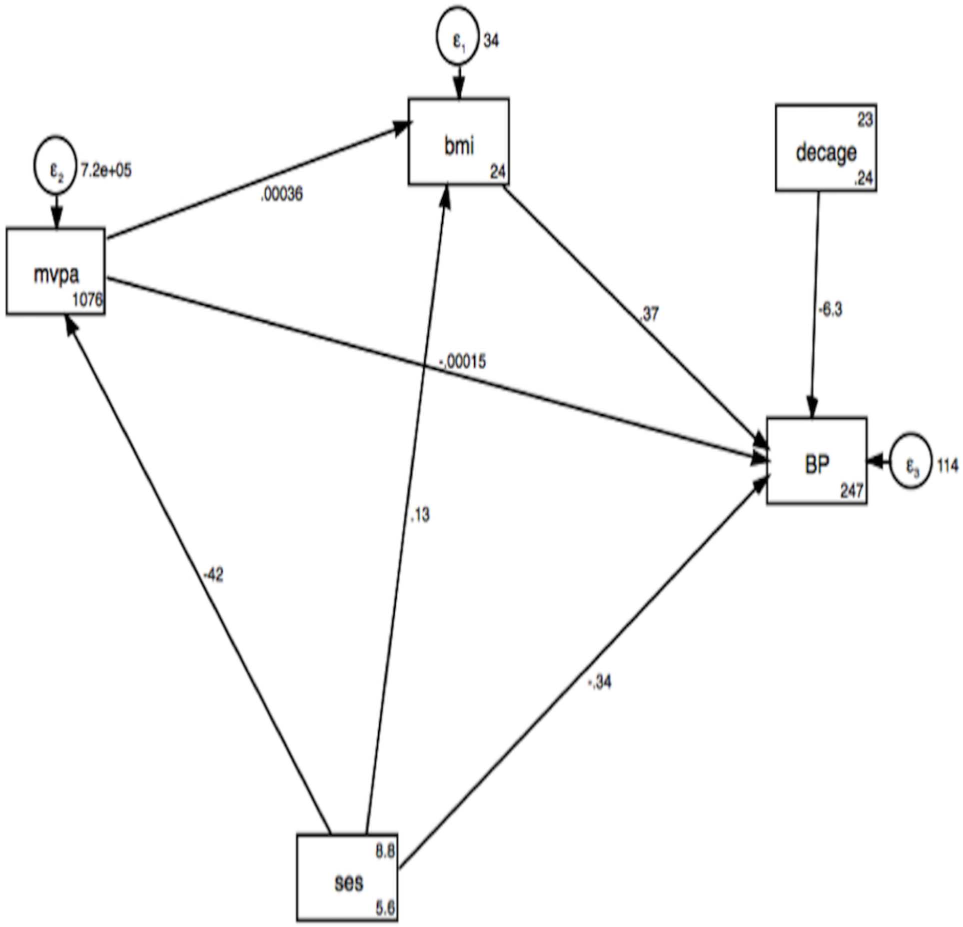

Structural equation model for SES, MVPA and BMI on SBP in urban

90x90mm (300 x 300 DPI)

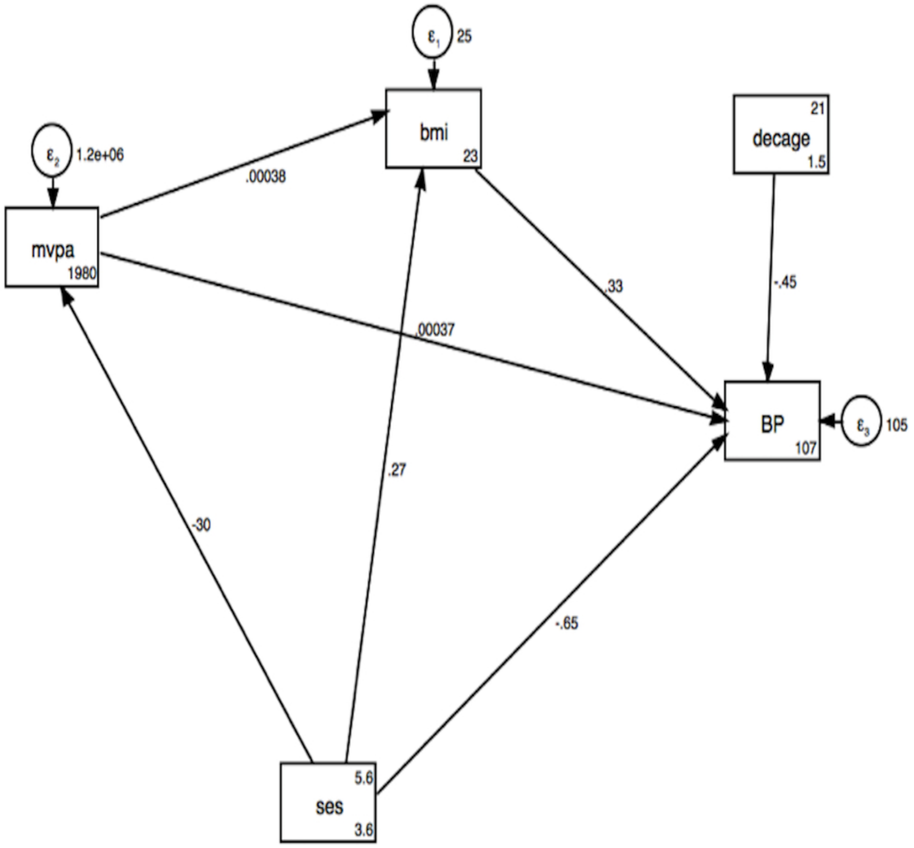

Structural equation model for SES, MVPA and BMI on SBP in rural

90x90mm (300 x 300 DPI)

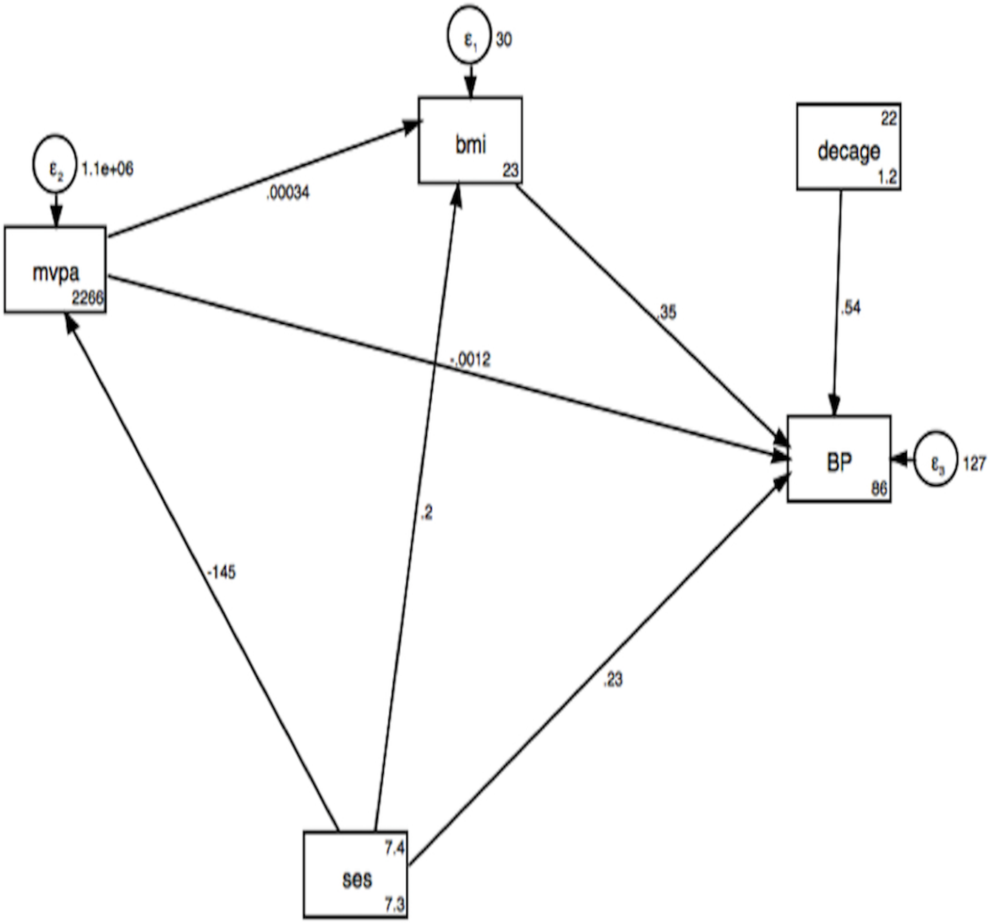

Structural equation model for SES, MVPA and BMI on SBP pooled

90x90mm (300 x 300 DPI)

STROBE Statement—checklist of items that should be included in reports of observational studies

	Item No	Recommendation
**Title and abstract**	1	(*a*) Indicate the study's design with a commonly used term in the title or the abstract **MS Page 4 Par 2**
		(*b*) Provide in the abstract an informative and balanced summary of what was done and what was found **MS Page 4**
**Introduction**		
Background/rationale	2	Explain the scientific background and rationale for the investigation being reported **MS Pages 5-6**
Objectives	3	State specific objectives, including any prespecified hypotheses **MS Page 6 Par 2**
**Methods**		
Study design	4	Present key elements of study design early in the paper **Methods: MS Page 6 Par 3**
Setting	5	Describe the setting, locations, and relevant dates, including periods of recruitment, exposure, follow-up, and data collection **Methods: MS Page 6 Par 3**
Participants	6	(*a*) *Cohort study*—Give the eligibility criteria, and the sources and methods of selection of participants. Describe methods of follow-up **N/A**<br><br>*Case-control study*—Give the eligibility criteria, and the sources and methods of case ascertainment and control selection. Give the rationale for the choice of cases and controls **N/A**<br>*Cross-sectional study*—Give the eligibility criteria, and the sources and methods of selection of participants **Methods: MS Page 6 Par 3**
		(*b*) *Cohort study*—For matched studies, give matching criteria and number of exposed and unexposed **N/A**<br>*Case-control study*—For matched studies, give matching criteria and the number of controls per case **N/A**
Variables	7	Clearly define all outcomes, exposures, predictors, potential confounders, and effect modifiers. Give diagnostic criteria, if applicable **Methods: MS Page 6 Par 4 – Page 8 Par 1**
Data sources/ measurement	8*	For each variable of interest, give sources of data and details of methods of assessment (measurement). Describe comparability of assessment methods if there is more than one group **Methods: MS Page 6 Par 4 – Page 8 Par 1**
Bias	9	Describe any efforts to address potential sources of bias **Methods: MS Page 6 Par 3**
Study size	10	Explain how the study size was arrived at **Methods: MS Page 6 Par 3**
Quantitative variables	11	Explain how quantitative variables were handled in the analyses. If applicable, describe which groupings were chosen and why **Methods: MS Page 8 Par 2**
Statistical methods	12	(*a*) Describe all statistical methods, including those used to control for confounding **Statistical analyses: MS Page 8 Par 2 and 3**
		(*b*) Describe any methods used to examine subgroups and interactions **N/A**
		(*c*) Explain how missing data were addressed **Statistical analyses: MS Page 8 Par 2**
		(*d*) *Cohort study*—If applicable, explain how loss to follow-up was addressed<br>*Case-control study*—If applicable, explain how matching of cases and controls was

For peer review only - http://bmjopen.bmj.com/site/about/guidelines.xhtml

addressed **N/A**

Cross-sectional study—If applicable, describe analytical methods taking account of sampling strategy **N/A**

(*e*) Describe any sensitivity analyses **N/A**

Continued on next page

For peer review only

**Results**		
Participants	13*	(a) Report numbers of individuals at each stage of study—eg numbers potentially eligible, examined for eligibility, confirmed eligible, included in the study, completing follow-up, and analysed **Results: Page 8 Par 5 – Page 9 Par 1**
		(b) Give reasons for non-participation at each stage **N/A**
		(c) Consider use of a flow diagram **N/A**
Descriptive data	14*	(a) Give characteristics of study participants (eg demographic, clinical, social) and information on exposures and potential confounders **Page 8 Par 5 – Page 9 Par 1**
		(b) Indicate number of participants with missing data for each variable of interest **Results: Page 8 Par 5**
		(c) *Cohort study*—Summarise follow-up time (eg, average and total amount**) N/A**
Outcome data	15*	*Cohort study*—Report numbers of outcome events or summary measures over time **N/A**
		Case-control study—Report numbers in each exposure category, or summary measures of exposure **N/A**
		Cross-sectional study—Report numbers of outcome events or summary measures **Results: Page 8 Par 5**
Main results	16	(*a*) Give unadjusted estimates and, if applicable, confounder-adjusted estimates and their precision (eg, 95% confidence interval). Make clear which confounders were adjusted for and why they were included **Results: Page 9 Par 1**
		(*b*) Report category boundaries when continuous variables were categorized **N/A**
		(*c*) If relevant, consider translating estimates of relative risk into absolute risk for a meaningful time period **N/A**
Other analyses	17	Report other analyses done—eg analyses of subgroups and interactions, and sensitivity analyses **N/A**
**Discussion**		
Key results	18	Summarise key results with reference to study objectives **Discussion: Page 9 Par 2**
Limitations	19	Discuss limitations of the study, taking into account sources of potential bias or imprecision. Discuss both direction and magnitude of any potential bias **Discussion: Page 11 Par 4**
Interpretation	20	Give a cautious overall interpretation of results considering objectives, limitations, multiplicity of analyses, results from similar studies, and other relevant evidence **Page 9 Par 3 – Page 11 Par 3**
Generalisability	21	Discuss the generalisability (external validity) of the study results **Discussion: Page 9 Par 3 – Page 11**
**Other information**		
Funding	22	Give the source of funding and the role of the funders for the present study and, if applicable, for the original study on which the present article is based **Funding: Page 12 Par 1**

*Give information separately for cases and controls in case-control studies and, if applicable, for exposed and unexposed groups in cohort and cross-sectional studies.

Note: An Explanation and Elaboration article discusses each checklist item and gives methodological background and published examples of transparent reporting. The STROBE checklist is best used in conjunction with this article (freely available on the Web sites of PLoS Medicine at http://www.plosmedicine.org/, Annals of Internal Medicine at http://www.annals.org/, and Epidemiology at http://www.epidem.com/). Information on the STROBE Initiative is available at www.strobe-statement.org.

For peer review only

For peer review only - http://bmjopen.bmj.com/site/about/guidelines.xhtml

BMJ Open

Body composition and physical activity as mediators in the relationship between socio-economic status and blood pressure in young South African women: A structural equation model analysis

Journal:	*BMJ Open*
Manuscript ID	bmjopen-2018-023404.R1
Article Type:	Research
Date Submitted by the Author:	12-Jul-2018

Complete List of Authors:	Munthali, Richard; University of the Witwatersrand, MRC/Wits Developmental Pathways for Health Research Unit<br>Manyema, Mercy; Wits University, Epidemiology and Biostatistics<br>Said-Mohamed, Rihlat; University of the Witwatersrand, MRC/Wits Developmental Pathways for Health<br>Kagura, Juliana; University of Witwatersrand, Paediatrics and Child health<br>Tollman, Stephen; University of the Witwatersrand,<br>Kahn, Kathleen; University of the Witwatersrand,<br>Gómez-Olivé, F. Xavier; University of the Witwatersrand, Medical Research Council/Wits Rural Health and Health Transitions Unit (Agincourt), School of Public Health, Faculty of Health Sciences<br>Micklesfield, Lisa; University of the Witwatersrand, MRC/Wits Developmental Pathways for Health Research Unit<br>Dunger, David; University of Cambridge, Paediatrics<br>Norris, Shane; University of Witwatersrand, Paediatrics and Child Health

**Primary Subject Heading**:	Epidemiology
Secondary Subject Heading:	Public health, Epidemiology
Keywords:	Hypertension < CARDIOLOGY, Obesity, Socioeconomic status, Physical activity, Structural equation model, Body mass index

SCHOLARONE™
Manuscripts

Body composition and physical activity as mediators in the relationship between socio-economic status and blood pressure in young South African women: A structural equation model analysis

Richard J Munthali[1], Mercy Manyema[1, 2], Rihlat Said-Mohamed[1], Juliana Kagura[1], Stephen Tollman [3,4,5], Kathleen Kahn[3, 4,5], F. Xavier Gómez-Olivé[3], Lisa K. Micklesfield[1], David Dunger [6,1], Shane A. Norris[1]

Affiliations:

[1]MRC/WITS Developmental Pathways for Health Research Unit, Department of Paediatrics, School of Clinical Medicine, Faculty of Health Sciences, University of the Witwatersrand, 7 York Rd, Parktown 2193, Johannesburg, South Africa

[2]DST-NRF Centre of Excellence in Human Development, University of the Witwatersrand, Johannesburg, South Africa

[3]MRC/Wits Rural Public Health and Health Transitions Research Unit, School of Public Health, Faculty of Health Sciences, University of the Witwatersrand, Johannesburg, South Africa

[4] INDEPTH Network, Accra, Ghana

[5]Umeå Centre for Global Health Research, Sweden.

[6]Department of Paediatrics, MRL Wellcome Trust-MRC Institute of Metabolic Science, NIHR Cambridge Comprehensive Biomedical Research Centre, University of Cambridge, Box 116, Addenbrooke's Hospital, Hills Road, Cambridge CB2 0QQ, UK

Conflict of interest: The views expressed in the submitted article are our own and not an official position of the affiliated institutions or funder. Authors have no financial relationships relevant to this article to disclose.

Corresponding Author: Dr. Richard J. Munthali

MRC/Wits Developmental Pathways for Health Research Unit

University of the Witwatersrand

Johannesburg

Tel: +27119331122

Email: munthali@aims.ac.za

For peer review only

Authors' emails:

Richard Junganiko Munthali: munthali@aims.ac.za
Mercy Manyema: mercy.manyema@gmail.com
Rihlat Said-Mohamed: rihlat.saidmohamed@wits.ac.za
Juliana Kagura: julianakagura@gmail.com
Stephen Tollman: stephen.tollman@wits.ac.za
Kathleen Kahn: kathleen.kahn@wits.ac.za
F. Xavier Gómez-Olivé: F.Gomez-OliveCasas@wits.ac.za
Lisa K. Micklesfield: lisa.micklesfield@wits.ac.za
David Dunger: dbd25@cam.ac.uk
Shane A Norris: shane.norris@wits.ac.za

For peer review only

Abstract

Objectives Varying hypertension prevalence across different socio-economic strata within a population has been well reported. However the causal factors and pathways across different settings are less clear, especially in sub-Saharan Africa. Therefore, this study aimed to compare blood pressure (BP) levels, and investigate the extent to which socioeconomic status (SES) is associated with blood pressure, in rural and urban South Africa women.

Setting Rural and urban South Africa.

Design Cross-sectional.

Participants Cross-sectional data on SES, total moderate-vigorous physical activity (MVPA), anthropometric and blood pressure were collected on rural (n=509) and urban (n=510) young black women (18-23 years age). Pregnant and mentally or physically disabled women were excluded from the study.

Results The prevalence of combined overweight and obesity (46.5% versus 38.8%) and elevated BP (27.0% versus 9.3%) were higher in urban than rural women respectively. Results from the structural equation modelling showed significant direct positive effects of body mass index (BMI) on systolic BP (SBP) in rural, urban and pooled datasets. Negative direct effects of SES on SBP and positive total effects of SES on SBP were observed in the rural and pooled datasets respectively. In rural young women, SES had direct positive effects on BMI and was negatively associated with MVPA in urban and pooled analyses. BMI mediated the positive total effects association between SES and SBP in pooled analyses (ß; 95%CI, 0.46; 0.15 to 0.76).

Conclusions Though South Africa is undergoing nutritional and epidemiological transitions; the prevalence of elevated BP still varies between rural and urban young women. The association between SES and SBP varies considerably in economically diverse populations with BMI being the most significant mediator. There is a need to tailor prevention strategies to take into account optimizing BMI when designing strategies to reduce future risk of hypertension in young women.

Keywords Blood pressure, Body mass index, Hypertension, Obesity, Urban, Rural, Socioeconomic status, Structural equation model, Physical activity

For peer review only

Strengths

1. The use of structural equation modelling allowed us to explore direct and indirect (mediation) effects of social economic status, physical activity and body mass index on elevated blood pressure from a representative sample of rural and urban populations of South African young women.
2. Although the urban and rural cohorts were from two different studies, the same research unit conducted both studies and, therefore, the data collection and management process were consistent between the two sites, thereby allowing for accurate comparison.

Limitations

1. Other unmeasured data, such as undernutrition in infancy, and dietary patterns were not included in the current analyses.
2. The low reliability of self-report data on physical activity could introduce bias. Thus, there is need for more accurate, objective measures of physical activity to strengthen the results of our analysis.
3. There is a need to do comparison on longitudinal data, especially as the socioeconomic environment is changing rapidly due to rural-urban labor migration and other factors would be helpful to examine these associations over time.

For peer review only

Introduction

High blood pressure (BP) or hypertension is a leading risk factor accounting for 7% of global disability-adjusted life years (DALYs) and contributing to the 34.5 million non-communicable disease (NCD) related deaths in 2010 [1, 2]. A recent global meta-analysis, involving 19.1 million individuals, reported that on average there has been a decrease in BP globally, but low- to middle-income countries (LMICs) have seen an increase in hypertension [3]. The prevalence of high BP in LMICs is estimated at 30% [4, 5] and it is the most significant risk factor for cardiovascular disease, most notably stroke [6]. In 2000, hypertension was estimated to have caused 9% of all deaths and over 390 000 DALYs in South Africa. Further, hypertension contributed to 50% of all strokes and 42% of ischaemic heart disease (IHD), signifying a substantial public health burden [7]. A systematic review of sub-Saharan African (SSA) data shows prevalence rates of hypertension of up to 41% with higher prevalence rates noted in urban compared to rural populations [8, 9]. A study in men and women aged 40 to 60 years of age in six sites across four SSA countries, including South Africa, showed the same trend with South African urban and rural cohorts having the highest prevalence of hypertension (41.6 to 54.1%) [10].

LMICs are experiencing both epidemiological and nutritional transitions with urban populations further along the transition as demonstrated by the higher prevalence of obesity and NCDs [4, 5, 8, 10-15]. Some evidence has shown that there are differences in the levels of BP between rural and urban settings [8], while other studies have found no significant differences [16]. According to Glass and McAtee, internal biological systems are sculpted by an interaction between genes and prolonged exposure to particular external environments, a principle they call embodiment [17]. Thus the differences in built and social environments between rural and urban settings may explain the differences in disease prevalence. A Ghanaian study showed that both systolic blood pressure (SBP) and diastolic blood pressure (DBP) were significantly lower in rural participants compared to urban participants [18]. However, a similar study in adolescents found that BP levels were only lower in rural boys, with no difference in the girls [19]. Pediatric and adolescent hypertension have been reported to track into adulthood in a South African urban population [20]. Results on elevated BP from studies in rural South African children have reported prevalence rates varying from 1.0% to 25.4% [21-24]. The factors explaining these differences have not been fully studied in LMICs.

Socioeconomic factors such as education, household income and household assets have been associated with BP levels [25-27]. In a US cohort of young adults, a higher household income remained associated with lower SBP even after controlling for all potential covariates including age, sex and bio-behavioral factors [28]. Similarly, in a French sample of 30-79 year olds, SBP independently increased and was inversely associated with both individual education and residential neighborhood education [29]. Studies in African countries have also found varying associations between socioeconomic status (SES) and BP patterns, with both positive and negative associations reported [8, 30, 31]. Some studies have speculated that the association between SES and body mass index (BMI), physical activity levels, diet, smoking, alcohol intake and malnutrition may influence BP patterns [18, 28, 31, 32].

For peer review only

For peer review only - http://bmjopen.bmj.com/site/about/guidelines.xhtml

Physical activity has been inversely associated with blood pressure and BMI directly associated with BP in more advanced economies, but inconsistent associations have been reported in LMICs [25, 33-37].

There is a need to examine BP and its determinants in young South African adults given the high rates of overweight and obesity and hypertension observed in this age group [20, 38]. Recent South African reports also indicate that the highest pregnancy rates occur in the age range of 20-24 years, with 26.2% of births reported, followed closely by the 25-29 year age group (25.7%) [39], and therefore targeting young adult women would also reduce adverse health outcomes in their children. It is important to closely examine rural-urban differences in hypertension due to differences in the epidemiology of obesity and SES divergence in the South African context, in order to better suit interventions to the different settings [23, 26, 30, 40-43]. Therefore, this study aims to compare BP levels between rural and urban young adult South African women, and to determine whether there is an association between SES and BP, and whether it is mediated by physical activity and BMI.

Methods

Study sample and site

The rural Agincourt site, 2016 potential the female participants between the ages of 18 and 23 years were in the existing Agincourt Health and Socio-demographic Surveillance System database [44]. Only 996 were located during the data collection period and were invited to participate and of these, 509 female participants were recruited. The urban sample consisted of 510 young women between the ages of 22 and 23 years who were randomly selected from the sample of 720 females who were part of the Birth-to-Twenty plus (BT20+) Young Adult Survey [45, 46]. Young women (n=51; 33 in rural and 18 in urban) who were pregnant at the time of the study were excluded, see the study design flow chart in **Figure 1**. Measurements and questionnaires were completed by trained research assistants and nurses, and were standardised between both sites, to eliminate biases. The study protocols were approved by the Human Research Ethics Committee of the University of the Witwatersrand (Clearance certificates M120138 for the Ntshembo-Hope Cross Sectional Survey in Agincourt and M111182 for the BT20+ survey). Written consent to participate was provided by participants, and mentally or physically disabled women were excluded from the study.

Patient and Public Involvement

No patients private or public were involved in this study, as it was a community population based.

Blood pressure

Blood pressure (mm Hg) was the outcome variable and it was measured using an Omron 6 automated machine (Kyoto, Japan). A five minute seated rest was observed before taking the BP measurements. Participants' seated BP was measured three times on the right side, with a 2-minute interval between each measurement. The mean for the

For peer review only

second and third readings was recorded for the current analysis. We had various cuff sizes and the appropriate size was used to accommodate differences in arm circumference.

According to the Seventh Report of the Joint National Committee on Prevention, Detection, Evaluation, and Treatment of High Blood Pressure [47], five categories of BP have been established for adults 18 years of age and older as shown in Table 1. These cut-offs were utilized in the current study. Prehypertension and hypertension were combined to create a new variable called elevated BP.

Table 1: Blood pressure classification [47]

Classification	Systolic Blood Pressure		Diastolic Blood Pressure
Low	<90	or	< 60
Normal	<120	and	<80
Prehypertension	120-139	or	80-90
High: Stage 1 Hypertension	140-159	or	90-99
High: Stage 2 hypertension	≥160	or	≥100

SBP was used in structural equation models (SEM) as it is more relevant in adults, and a good predictor of adverse health outcomes later in life [48], such as CVDs.

Anthropometry

At both sites, participants' height and weight were measured by trained research assistants using standard techniques [49, 50] . Weight was measured in light clothing and barefoot to the nearest 0.1 kg using a digital scale (Tanita model TBF-410; Arlinghton Heights; USA). Height was measured barefoot to the nearest 0.1 cm using a stadiometer (Holtain, Crymych, UK). Waist circumference was measured with a non-stretchable fibreglass tape at the level of the umbilicus. BMI was calculated as weight/height^2 (kg/m^2).

Socio-economic status (SES)

For peer review only

Physical assets owned in the participants' household were used as a proxy for SES index [51]. It was generated by summing the number of assets owned in the household from the following: television, car, washing machine, fridge, phone, radio, microwave, cell phone, DVD/Video, DSTV (cable channel), computer, internet, medical aid. Previous studies in this population have shown that the sum of physical assets (household assets) is closely related to the household per capital expenditure and household income [51-53]. The household SES is regarded as a good measure of accumulated household wealth so it is a more reflective wealth index than income of a household's wealth over time.

Physical activity

The Global Physical Activity Questionnaire (GPAQ), developed for global physical activity surveillance, was completed via interview to obtain self-reported physical activity [54]. Total MVPA in minutes per week (mins/wk) was calculated by adding occupation, travel-related and leisure time moderate and vigorous intensity physical activity. Sitting time (mins/wk) was used as a proxy for sedentary time.

Statistical analyses

Analysis of variance and student's t test, and Chi-squared tests and Wilcoxon rank sum test for non-parametric variables, were conducted to compare study characteristics between urban and rural young women. Structural equation modeling (SEM), with missing data option, was used to test and estimate the direct and indirect associations between variables, most especially the mediation roles of physical activity (MVPA) or sedentary time (sitting), and body composition (BMI and WC), in the association between SES and SBP. SEMs allow us to assess the mediation effects of multiple mediators [55]. SEM decomposed SES-BP associations into two parts, direct (unmediated) and indirect (mediated through MVPA/sitting and BMI/WC).

Direct, indirect and total effects were computed and recorded, and the proportion of the total effect mediated was calculated. To evaluate the best fitting model for our data, we calculated different goodness of fit indices including Chi-squared test, Root mean squared error of approximation (RMSEA), Comparative fit index (CFI), Tucker-Lewis index (TLI) and Standardized root mean squared residual (SRMR) [56]. Though the Chi-squared test has been popularly used as a goodness of fit index, it has been reported to be biased and not reliable as the only goodness of fit index. It is also highly sensitive to sample size [57, 58] , and often inflated with non-normal data such as physical activity data and we therefore employed the Hu and Bentler's Two-Index Presentation Strategy (1999) combination rule, with cut off values depending on the fitness index, to determine the best model fit [56, 59]. We estimated the coefficients (β) with 95% confidence intervals (95% CI) for the direct, indirect and total effects and also calculated the proportion of association mediated by indirect effects. If the direct and indirect effects had opposite signs (negative or positive effects) the proportion mediated was assessed using the absolute values for all indirect and direct effects [60].

All the analyses were conducted using STATA (version 13.0; STATA Corp., College Station, TX, USA). We confirmed SEM results by running the SEM with the Satorra–Bentler and Huber-White (Robust) Sandwich

For peer review only - http://bmjopen.bmj.com/site/about/guidelines.xhtml

Estimator options [61] in STATA (version 15.1; STATA Corp., College Station, TX, USA). These options relax the normality assumption hence are robust to non-normal data, which would be the case for mvpa and SES in the current study. A P-value < 0.05 was considered statistically significant.

Results

Study characteristics

Descriptive statistics for the non-pregnant study participants (urban, n=492; rural, n=476) are presented in Table 2. There was no difference in BMI or waist circumference between the urban and rural participants, but the prevalence of overweight and obesity was significantly higher in the urban (46.5%) compared to the rural young women (38.8%). Household SES was significantly higher in the urban compared to the rural group. Self-reported MVPA was significantly higher in the rural than urban women (p<0.001), and the urban women spent significantly more time sitting than their rural counterparts (p<0.001). Systolic and diastolic BP were significantly higher in the urban group, as was the prevalence of elevated BP (27.0 vs. 9.3%).

Structural equation models for body mass index and Waist circumference

Results from the SEMs for SES associations with SBP via MVPA and BMI are presented in Tables 3a, 3b and 3c for urban, rural and pooled analyses respectively, and also shown in Figures 1, 2, 3. No significant direct or indirect effects via (MVPA or BMI) of SES on SBP were observed in either the urban or rural women, but there were significant direct effects of SES on MVPA. Results showed that individuals with a higher SES index were less likely to be physically active in pooled data and urban women. In rural women, a one-unit increase in total household assets was associated with a decrease of 0.65 mmHg (95% CI: -1.19 to -0.10) in SBP and an increase of 0.27 kg/m^2 in BMI (95% CI: 0.1 to 0.53) (**Tables 3a, 3b and Figures 2, 3)**. The SEM for the pooled sample showed a significant indirect effect of household SES on SBP via BMI, with 50% of the total effect being mediated by BMI **(Table 3c and Figure 4).** Direct positive effects of BMI on SBP were observed in both settings and the pooled sample with a 1 kg/m^2 increase in BMI being associated with an increase of 0.37 mmHg (95% CI: 0.21 to 0.53) and 0.33 (95% CI: 0.12 to 0.54) mmHg SBP in urban and rural young women, respectively. Similar results were observed when including waist circumference as the body composition indicator (data not shown).

Discussion

For peer review only

A rising prevalence of hypertension has been reported in South Africa. Peer and colleagues reported a higher prevalence in 2008 (35.6%) compared to 1990 (21.6 %) in men and women aged 25-74 years in an urban black community in Cape Town, South Africa [40]. We have shown in young adult women from urban and rural South Africa, an overall elevated BP prevalence of 18.4 % (27.0 % in urban and 9.3 % in rural). We have also shown a direct effect of BMI on SBP in the urban and rural women separately, as well as when pooled, thereby providing further evidence of an association between overall adiposity and blood pressure. The total effects of SES on SBP were the same in both settings.

Prevalence data on elevated BP and hypertension from other countries in SSA have shown conflicting results when comparing urban and rural communities. In Malawi, a higher prevalence of hypertension in urban compared to rural communities has been reported and attributed to differences in lifestyle as rural communities participate in subsistence based agricultural activities while the urban community has a more westernized lifestyle with higher salt intake and lower physical inactivity [9]. Similarly, data from Ghana have shown a higher mean SBP and DBP and a higher prevalence of hypertension in urban communities [18, 62]. In the PURE study in South Africa, Pisa and colleagues reported that both urban adult men and women had higher mean blood pressures in comparison to their rural peers though the overall CVD risk factors were equally prevalent in both settings [41]. In contrast, findings from Cameroon have reported a higher BP prevalence in rural compared to urban men and women older than 40 years old, while Kenyan studies have reported no significant differences [16, 63]. Results from six urban and rural sites in four SSA countries – Kenya, South Africa, Ghana and Burkina Faso – have reported a prevalence of hypertension in women aged between 40 and 60 years ranging from 15.1% in rural Burkina Faso to 54.1% in urban South Africa [10]. It was also reported that in all three South African sites, both rural and urban, the prevalence of hypertension was higher than in the other three countries [10]. These findings show the complex health transitions occurring in SSA and the impact that this is having on cardio-metabolic disease risk.

Our study showed significant differences in SES between the urban and rural samples, as well a big variation in SES within these two settings. The social patterning of CVD risk factors, including hypertension, in SSA and LMICs has in part been attributed to differences in countries' socioeconomic development. Previous results from five countries, (two high income and three LMICs), reported that hypertension and other CVD risk factors were substantially associated with education and wealth status; individuals with less education and lower wealth generally showing higher prevalence of CVD risk factors [64] . The effect of SES in this study is most evident in the rural women for whom household SES was lower (compared to urban) and who may be transitioning faster (both nutritionally and economically) than the urban young women. Though SES is positively associated with BMI in rural young women, it is negatively associated with SBP. There may be other factors, such as physical activity due to agricultural activities or dietary patterns, which were not recorded. In addition, the weight gain observed might not be due to fat mass, which has been reported to be positively associated with SBP before [65], but rather to muscle mass and bone mass.

In Mexico, women in rural and upper SES categories were likely to have a higher SBP, while we have reported that a higher SES was associated with a decrease in SBP in rural communities. At a population level, there is a need to

For peer review only - http://bmjopen.bmj.com/site/about/guidelines.xhtml

consider different SES categories and monitor the effects of transitioning from one SES category to another on hypertension, since these categories may respond differently to an increase or a decrease in their SES. Kagura and colleagues tracked SES in South African children and reported that moving from the low SES in infancy to a higher SES in adolescence had a protective effect on SBP level in young adulthood [26]. Our results have shown that this could be more pronounced in rural areas.

We observed a positive association between SES and BMI in the rural sample and the same direction of effects was observed in the urban, though not significant. This is in line with results reported in many LMICs including South Africa, but in contrast with those reported in higher income populations [33, 34, 64]. A systematic review of studies between 1989 to 2007 reported that SES was positively associated with obesity in the middle transitioning economies such as South Africa and Jamaica [66]. We have shown that both in the rural and urban participants (not significant), a higher SES resulted in reduced SBP, while the pooled analysis showed a positive total effect association between SES and SBP. This could be due to the introduction of more variation in SES when data from both sites are pooled; with many individuals with low SES in the rural area, the associations became skewed towards the low SES individuals. This may suggest that different transitional levels of SES have different effects on hypertension risk depending on the environment (either urban or rural). Though not significant, the total effects of SES on SBP are the same in both rural and urban hence the differences in prevalence cannot be explained by the setting or SES alone. In urban and rural settings of four countries (Kenya, Namibia, Nigeria and Tanzania), the prevalence of age standardized hypertension was similarly high and ranging from 19.3 % to 38.0 % [11]. Cois and colleagues reported that a higher SES was associated with lower SBP in a nationally representative sample of South African women [25] using SEM models. Alcohol use, PA, smoking and resting heart rate and BMI were reported to be the mediators of the indirect of the association between SES and SBP in men but not in women, suggesting that other factors may play a major role in women [25]. Similarly, our results show that neither PA nor BMI mediate the association between SES and SBP in urban and rural settings, suggesting that other factors may explain the association. Among those, dietary patterns and stress have been reported to be independently associated with SBP [67, 68].

The significant direct associations between BMI and SBP are in line with other findings in South Africa and within the SSA region [11, 33, 40, 42, 69, 70]. This link was consistent in rural, urban and pooled data sets, indicating the importance of BMI in the aetiology of high BP. Munthali and colleagues reported that the link between obesity and hypertension could be observed as early as five years of age. Children with early onset of obesity were at higher risk of developing hypertension in late adolescence [38].

In this study, using SEM models to explore the mediation role of BMI and PA helped quantify potential contributions of these variables to the effect of SES on SBP. The results show that PA was not a significant mediator in the association between SES and BP in the urban or the rural samples. SES was negatively associated with MVPA in urban and pooled samples, indicating that as individuals transition from low to higher SES, they reduce their physical activity level. We speculate that these differences in the association between SES and SBP in both our rural and urban results and in those from high-income countries are due to differences in levels of nutritional and

For peer review only - http://bmjopen.bmj.com/site/about/guidelines.xhtml

epidemiological transition in these regions [71, 72]. Those with low SES in high-income countries are likely to consume cheaper, more energy dense foods, participate in less leisure time PA and be more sedentary [73, 74] In LMICs, agricultural activities remain a part of everyday life and a day-to-day activity in rural living, while those with higher SES in the same settings rapidly adopt the westernized life style with less PA, fewer agricultural activities and home grown food. However, this speculation is not supported by the data on PA in this study despite the rural participants having a higher PA. Our understanding of the Agincourt rural economy is that agriculture is quite a minor aspect though very useful to augment the household income.

The limitations of this study are that other unmeasured data, such as undernutrition in infancy, which is a known risk factor for high BP later in life [75], and dietary patterns were not included in the current analyses. We are currently working on research to address this limitation. We can also not rule out the role of genetics. Secondly, the low reliability of self-report data on PA could introduce bias. Thus, there is need for more precise, objective measures of physical activity to strengthen the results of our analysis. Lastly, longitudinal data, especially as the socioeconomic environment is changing rapidly due to rural-urban labor migration and other factors would be helpful to examine these associations over time. The cross-sectional design lacks a temporal component between the factors analyzed. Thus, it is difficult to say anything certain about the direction of the associations, hence the need for the longitudinal data.

Conclusions

Though the prevalence of overweight or obesity is relatively higher in both rural and urban than those reported in other SSA countries, women in the urban setting were at more risk for elevated blood pressure than their rural counterparts. The link between SES and SBP varies in a more economically diverse population, as seen with the pooled rural and urban dataset, with BMI being the most likely mediator. There is need to consider optimizing BMI as a key intervention strategy in young adults in part to combat hypertension. Our findings should be replicated with prospective data.

Funding

Norris is supported by the UK MRC DfID African Research Leader Scheme and by the DST-NRF Centre of Excellence in Human Development at the University of the Witwatersrand, Johannesburg. Birth to Twenty data collection was supported by the Wellcome Trust under Grant [092097/Z/10/Z]. The MRC/Wits- Agincourt Unit is supported by the South African Medical Research Council, and the Wellcome Trust under Grants [058893/Z/99/A, 069683/Z/02/Z, 085477/Z/08/Z, 085477/B/08/Z]. Opinions expressed and conclusions arrived at, are those of the authors and are not to be attributed to the CoE in Human Development.

Conflict of interest

For peer review only - http://bmjopen.bmj.com/site/about/guidelines.xhtml

Authors have no conflicts of interest to disclose.

Consent for publication

Not applicable

Data Sharing

The datasets used and/or analysed during the current study are available from the Developmental Pathways for Health Research Unit data management department by contacting Prof. Shane A Norris on reasonable request

Competing interest

The authors declare that they have no competing interests

Authors' contributions

RJM and SAN conceptualized the manuscript. RJM analyzed the data. RJM MM RSM JK ST KK FXG LKM DD SAN interpreted the data. RJM wrote the manuscript and all authors were involved in editing and approving the final manuscript.

Acknowledgements

We wish to thank the Bt20+ and Agincourt participants for taking part in the study and the Bt20+ and Agincourt team for their relentless support throughout the study.

Ethics Approval and Consent to Participate

Prior to the study, the study protocols were approved by the Human Research Ethics Committee of the University of the Witwatersrand (Clearance certificates M120138 for the Ntshembo-Hope Cross Sectional Survey in Agincourt and M111182 for the BT20+ survey). Independent written informed consent to participate was obtained from participants.

For peer review only

References

1. Lim SS, Vos T, Flaxman AD, Danaei G, Shibuya K, Adair-Rohani H, AlMazroa MA, Amann M, Anderson HR, Andrews KG *et al*: **A comparative risk assessment of burden of disease and injury attributable to 67 risk factors and risk factor clusters in 21 regions, 1990–2010: a systematic analysis for the Global Burden of Disease Study 2010**. *The Lancet* 2012, **380**(9859):2224-2260.
2. Lozano R, Naghavi M, Foreman K, Lim S, Shibuya K, Aboyans V, Abraham J, Adair T, Aggarwal R, Ahn SY: **Global and regional mortality from 235 causes of death for 20 age groups in 1990 and 2010: a systematic analysis for the Global Burden of Disease Study 2010**. *The Lancet* 2013, **380**(9859):2095-2128.
3. Ezzati M, Geleijnse J: **Worldwide trends in blood pressure from 1975 to 2015**. *The Lancet* 2017, **389**(10064):37-55.
4. Sarki AM, Nduka CU, Stranges S, Kandala N-B, Uthman OA: **Prevalence of Hypertension in Low- and Middle-Income Countries: A Systematic Review and Meta-Analysis**. *Medicine* 2015, **94**(50):e1959.
5. Chow CK, Teo KK, Rangarajan S, Islam S, Gupta R, Avezum A, Bahonar A, Chifamba J, Dagenais G, Diaz R: **Prevalence, awareness, treatment, and control of hypertension in rural and urban communities in high-, middle-, and low-income countries**. *Jama* 2013, **310**(9):959-968.
6. Kearney PM, Whelton M, Reynolds K, Muntner P, Whelton PK, He J: **Global burden of hypertension: analysis of worldwide data**. *The lancet* 2005, **365**(9455):217-223.
7. Norman R, Gaziano T, Laubscher R, Steyn K, Bradshaw D, Collaboration SACRA: **Estimating the burden of disease attributable to high blood pressure in South Africa in 2000**. *South African Medical Journal* 2007, **97**(8):692-698.
8. Addo J, Smeeth L, Leon DA: **Hypertension in Sub-Saharan Africa a systematic review**. *Hypertension* 2007, **50**(6):1012-1018.
9. Simmons D, Barbour G, Congleton J, Levy J, Meacher P, Saul H, Sowerby T: **Blood pressure and salt intake in Malawi: an urban rural study**. *Journal of epidemiology and community health* 1986, **40**(2):188-192.
10. Gómez-Olivé FX, Ali SA, Made F, Kyobutungi C, Nonterah E, Micklesfield L, Alberts M, Boua R, Hazelhurst S, Debpuur C: **Stark Regional and Sex Differences in the Prevalence and Awareness of Hypertension: An H3Africa AWI-Gen Study Across 6 Sites in Sub-Saharan Africa**. *Global Heart* 2017.
11. Hendriks ME, Wit FW, Roos MT, Brewster LM, Akande TM, de Beer IH, Mfinanga SG, Kahwa AM, Gatongi P, Van Rooy G: **Hypertension in sub-Saharan Africa: cross-sectional surveys in four rural and urban communities**. *PloS one* 2012, **7**(3):e32638.
12. Lemogoum D, Seedat YK, Mabadeje AF, Mendis S, Bovet P, Onwubere B: **International Forum for Hypertension control and prevention in Africa. Recommendations for prevention, diagnosis and management of hypertension and cardiovascular risk factors in sub-Saharan Africa**. *J Hypertens* 2003, **21**.
13. Schutte A, Botha S, Fourie C, Gafane-Matemane L, Kruger R, Lammertyn L, Malan L, Mels C, Schutte R, Smith W: **Recent advances in understanding hypertension development in sub-Saharan Africa**. *Journal of Human Hypertension* 2017.
14. Thorogood M, Connor MD, Lewando Hundt G, Tollman SM: **Understanding and managing hypertension in an African sub-district: A multidisciplinary approach1**. *Scandinavian Journal of Public Health* 2007, **35**(69 suppl):52-59.
15. Yusufali A, Khatib R, Islam S, AlHabib K, Kelishadi R, Rangarajan S, Yusuf S: **LBOS 03-01 PREVALENCE, AWARENESS, TREATMENT, AND CONTROL OF HYPERTENSION IN THE**

For peer review only - http://bmjopen.bmj.com/site/about/guidelines.xhtml

MIDDLE EAST: RESULTS FROM THE PROSPECTIVE URBAN RURAL EPIDEMIOLOGY (PURE) STUDY. *J Hypertens* 2016, **34 Suppl 1**:e551.

16. Poulter N, Khaw K, Hopwood B, Mugambi M, Peart W, Rose G, Sever P: **Blood pressure and its correlates in an African tribe in urban and rural environments**. *Journal of epidemiology and community health* 1984, **38**(3):181-185.
17. Glass TA, McAtee MJ: **Behavioral science at the crossroads in public health: extending horizons, envisioning the future**. *Social science & medicine* 2006, **62**(7):1650-1671.
18. Agyemang C: **Rural and urban differences in blood pressure and hypertension in Ghana, West Africa**. *Public Health* 2006, **120**.
19. Agyemang C, Redekop WK, Owusu-Dabo E, Bruijnzeels MA: **Blood pressure patterns in rural, semi-urban and urban children in the Ashanti region on Ghana, West Africa**. *BMC Public Health* 2005, **5**.
20. Kagura J, Adair LS, Musa MG, Pettifor JM, Norris SA: **Blood pressure tracking in urban black South African children: birth to twenty cohort**. *BMC pediatrics* 2015, **15**(1):1.
21. Goon D, Amusa L, Mhlongo D, Khoza L, Any-Anwu F: **Elevated blood pressure among Rural South African Children in Thohoyandou, South Africa**. *Iran J Pub Health* 2013, **42**.
22. Kemp C, Pienaar AE, Schutte AE: **The prevalence of hypertension and the relationship with body composition in Grade 1 learners in the North West Province of South Africa**. *South African Journal of Sports Medicine* 2011, **23**(4).
23. Monyeki K, Kemper H, Makgae P: **The association of fat patterning with blood pressure in rural South African children: the Ellisras Longitudinal Growth and Health Study**. *International journal of epidemiology* 2006, **35**(1):114-120.
24. Schutte A, Van Rooyen J, Huisman H, Kruger H, Malan N, De Ridder J: **Dietary risk markers that contribute to the aetiology of hypertension in black South African children: the THUSA BANA study**. *Journal of human hypertension* 2003, **17**(1):29-35.
25. Cois A, Ehrlich R: **Analysing the socioeconomic determinants of hypertension in South Africa: a structural equation modelling approach**. *BMC public health* 2014, **14**(1):414.
26. Kagura J, Adair LS, Pisa PT, Griffiths PL, Pettifor JM, Norris SA: **Association of socioeconomic status change between infancy and adolescence, and blood pressure, in South African young adults: Birth to Twenty Cohort**. *BMJ open* 2016, **6**(3):e008805.
27. Wilson DK, Kliewer W, Plybon L, Sica DA: **Socioeconomic status and blood pressure reactivity in healthy black adolescents**. *Hypertension* 2000, **35**(1):496-500.
28. Brummett BH, Babyak MA, Siegler IC, Shanahan M, Harris KM, Elder GH, Williams RB: **Systolic Blood Pressure, Socioeconomic Status, and Biobehavioral Risk Factors in a Nationally Representative US Young Adult Sample**. *Hypertension* 2011, **58**(2):161-166.
29. Chaix B, Bean K, Leal C, Thomas F, Havard S, Evans D, Jégo B, Pannier B: **Individual/Neighborhood Social Factors and Blood Pressure in the RECORD Cohort Study**. *Which Risk Factors Explain the Associations?* 2010, **55**(3):769-775.
30. Griffiths PL, Sheppard ZA, Johnson W, Cameron N, Pettifor JM, Norris SA: **Associations between household and neighbourhood socioeconomic status and systolic blood pressure among urban South African adolescents**. *Journal of biosocial science* 2012, **44**(04):433-458.
31. Longo-Mbenza B, Luila EL, M'Buyamba-Kabangu J: **Nutritional status, socio-economic status, heart rate, and blood pressure in African school children and adolescents.** *International journal of cardiology* 2007, **121**(2):171-177.
32. Aounallah-Skhiri H, El Ati J, Traissac P, Romdhane HB, Eymard-Duvernay S, Delpeuch F, Achour N, Maire B: **Blood pressure and associated factors in a North African adolescent population. A national cross-sectional study in Tunisia**. *BMC Public Health* 2012, **12**(1):1.

For peer review only - http://bmjopen.bmj.com/site/about/guidelines.xhtml

33. Afrifa–Anane E, Agyemang C, Codjoe SNA, Ogedegbe G, Aikins Ad-G: **The association of physical activity, body mass index and the blood pressure levels among urban poor youth in Accra, Ghana**. *BMC public health* 2015, **15**(1):269.
34. Anane AE, Agyemang C, Codjoe SNA, Ogedegbe G, Aikins A: **The association of physical activity, Body mass index and blood pressure among urban poor youth in Accra, Ghana**. *BMC Public Health* 2015, **15**.
35. Hu G, Barengo NC, Tuomilehto J, Lakka TA, Nissinen A, Jousilahti P: **Relationship of physical activity and body mass index to the risk of hypertension: a prospective study in Finland**. *Hypertension* 2004, **43**(1):25-30.
36. Juraschek SP, Blaha MJ, Whelton SP, Blumenthal R, Jones SR, Keteyian SJ, Schairer J, Brawner CA, Al-Mallah MH: **Physical fitness and hypertension in a population at risk for cardiovascular disease: the Henry Ford ExercIse Testing (FIT) Project**. *Journal of the American Heart Association* 2014, **3**(6):e001268.
37. Väistö J, Eloranta A-M, Viitasalo A, Tompuri T, Lintu N, Karjalainen P, Lampinen E-K, Ågren J, Laaksonen DE, Lakka H-M: **Physical activity and sedentary behaviour in relation to cardiometabolic risk in children: cross-sectional findings from the Physical Activity and Nutrition in Children (PANIC) Study**. *International Journal of Behavioral Nutrition and Physical Activity* 2014, **11**(1):55.
38. Munthali RJ, Kagura J, Lombard Z, Norris SA: **Childhood adiposity trajectories are associated with late adolescent blood pressure: birth to twenty cohort**. *BMC Public Health* 2016, **16**(1):1-10.
39. STATSSA: **Mortality and causes of death in South Africa, 2015: Findings from death notification**. In*.* Pretoria: Statistics South Africa; 2017 1–140. .
40. Peer N, Steyn K, Lombard C, Gwebushe N, Levitt N: **A high burden of hypertension in the urban black population of Cape Town: The Cardiovascular Risk in Black South Africans (CRIBSA) Study**. *PLoS One* 2013, **8**(11):e78567.
41. Pisa P, Behanan R, Vorster H, Kruger A: **Social drift of cardiovascular disease risk factors in Africans from the North West Province of South Africa: the PURE study: cardiovascular topics**. *Cardiovascular journal of Africa* 2012, **23**(7):371-388.
42. Steyn K, Fourie J, Lombard C, Katzenellenbogen J, Bourne L, Jooste P: **Hypertension in the black community of the Cape Peninsula, South Africa**. *East African medical journal* 1996, **73**(11):758-763.
43. Tibazarwa K, Ntyintyane L, Sliwa K, Gerntholtz T, Carrington M, Wilkinson D, Stewart S: **A time bomb of cardiovascular risk factors in South Africa: results from the Heart of Soweto Study "Heart Awareness Days"**. *International journal of cardiology* 2009, **132**(2):233-239.
44. Kahn K, Collinson MA, Gómez-Olivé FX, Mokoena O, Twine R, Mee P, Afolabi SA, Clark BD, Kabudula CW, Khosa A: **Profile: Agincourt health and socio-demographic surveillance system**. *International journal of epidemiology* 2012, **41**(4):988-1001.
45. Pradeilles R, Rousham EK, Norris SA, Griffiths PL: **Urban South African adolescents' perceptions of their neighborhood socio-economic environments: the Birth to Twenty plus cohort study**. *Children Youth and Environments* 2014, **24**(3):173-200.
46. Richter L, Norris S, Pettifor J, Yach D, Cameron N: **Cohort profile: Mandela's children: the 1990 Birth to Twenty study in South Africa**. *International journal of epidemiology* 2007, **36**(3):504-511.
47. Chobanian AV: **Joint National Committee on Prevention, Detection, Evaluation, and Treatment of High Blood Pressure. National Heart, Lung, and Blood Institute; National High Blood Pressure Education Program Coordinating Committee: Seventh report of the Joint National Committee on Prevention, Detection, Evaluation, and Treatment of High Blood Pressure**. *Hypertension* 2003, **42**:1206-1252.

For peer review only - http://bmjopen.bmj.com/site/about/guidelines.xhtml

48. Strandberg TE, Pitkala K: **What is the most important component of blood pressure: systolic, diastolic or pulse pressure?** *Current opinion in nephrology and hypertension* 2003, **12**(3):293-297.
49. Cameron N: **The measurement of human growth**: Taylor & Francis; 1984.
50. Lohman TG, Roche AF, Martorell R: **Anthropometric standardization reference manual**: Human kinetics books; 1988.
51. Houweling TA, Kunst AE, Mackenbach JP: **Measuring health inequality among children in developing countries: does the choice of the indicator of economic status matter?** *International journal for equity in health* 2003, **2**(1):8.
52. Feeley AB, Musenge E, Pettifor JM, Norris SA: **Investigation into longitudinal dietary behaviours and household socio-economic indicators and their association with BMI Z-score and fat mass in South African adolescents: the Birth to Twenty (Bt20) cohort**. *Public health nutrition* 2013, **16**(04):693-703.
53. Jones LL, Griffiths PL, Adair LS, Norris SA, Richter LM, Cameron N: **A comparison of the socio-economic determinants of growth retardation in South African and Filipino infants**. *Public health nutrition* 2008, **11**(12):1220-1228.
54. Bull FC, Maslin TS, Armstrong T: **Global physical activity questionnaire (GPAQ): nine country reliability and validity study**. *Journal of Physical Activity and health* 2009, **6**(6):790-804.
55. VanderWeele TJ: **Mediation analysis: a practitioner's guide**. *Annual review of public health* 2016, **37**:17-32.
56. Hu Lt, Bentler PM: **Cutoff criteria for fit indexes in covariance structure analysis: Conventional criteria versus new alternatives**. *Structural equation modeling: a multidisciplinary journal* 1999, **6**(1):1-55.
57. Schermelleh-Engel K, Moosbrugger H, Müller H: **Evaluating the fit of structural equation models: Tests of significance and descriptive goodness-of-fit measures**. *Methods of psychological research online* 2003, **8**(2):23-74.
58. Vandenberg RJ: **Introduction: Statistical and Methodological Myths and Urban Legends: Where, Pray Tell, Did They Get This Idea?** In.: Sage Publications Sage CA: Thousand Oaks, CA; 2006.
59. Fan X, Sivo SA: **Sensitivity of fit indexes to misspecified structural or measurement model components: Rationale of two-index strategy revisited**. *Structural Equation Modeling* 2005, **12**(3):343-367.
60. Alwin DF, Hauser RM: **The decomposition of effects in path analysis**. *American sociological review* 1975:37-47.
61. Williams R, Allison PD, Moral-Benito E: **Xtdpdml: Linear dynamic panel-data estimation using maximum likelihood and structural equation modeling**. In.; 2016.
62. Kerry SM, Emmett L, Micah FB, Martin-Peprah R, Antwi S, Phillips RO, Plange-Rhule J, Eastwood JB, Cappuccio FP: **Rural and semi-urban differences in salt intake, and its dietary sources, in Ashanti, West Africa**. *Ethnicity & disease* 2005, **15**(1):33-39.
63. Lissock CNAA, Sobngwi E, Ngassam E, Ngoa LSE: **Rural and urban differences in metabolic profiles in a Cameroonian population**. *Pan African Medical Journal* 2011, **10**.
64. Stringhini S, Forrester TE, Plange-Rhule J, Lambert EV, Viswanathan B, Riesen W, Korte W, Levitt N, Tong L, Dugas LR: **The social patterning of risk factors for noncommunicable diseases in five countries: evidence from the modeling the epidemiologic transition study (METS)**. *BMC Public Health* 2016, **16**(1):956.
65. de Hoog ML, van Eijsden M, Stronks K, Gemke RJ, Vrijkotte TG: **Association between body size and blood pressure in children from different ethnic origins**. *Cardiovascular diabetology* 2012, **11**(1):136.

For peer review only

For peer review only - http://bmjopen.bmj.com/site/about/guidelines.xhtml

66. Dinsa G, Goryakin Y, Fumagalli E, Suhrcke M: **Obesity and socioeconomic status in developing countries: a systematic review**. *Obesity reviews* 2012, **13**(11):1067-1079.
67. Masala G, Bendinelli B, Versari D, Saieva C, Ceroti M, Santagiuliana F, Caini S, Salvini S, Sera F, Taddei S: **Anthropometric and dietary determinants of blood pressure in over 7000 Mediterranean women: the European Prospective Investigation into Cancer and Nutrition-Florence cohort**. *Journal of hypertension* 2008, **26**(11):2112-2120.
68. Wilson BL, Albright GL, Steiner SS, Andreassi JL: **Cardiodynamic response to psychological and cold pressor stress: further evidence for stimulus response specificity and directional fractionation**. *Biofeedback and Self-regulation* 1991, **16**(1):45-53.
69. Goon D, Amusa L, Mhlongo D, Khoza L, Any-Anwu F: **Elevated blood pressure among rural South African children in Thohoyandou, South Africa**. *Iranian journal of public health* 2013, **42**(5):489.
70. Moselakgomo VK, Toriola AL, Shaw BS, Goon DT, Akinyemi O: **Body mass index, overweight, and blood pressure among adolescent schoolchildren in Limpopo province, South Africa**. *Revista Paulista de Pediatria* 2012, **30**(4):562-569.
71. Lynch JW, Smith GD, Kaplan GA, House JS: **Income inequality and mortality: importance to health of individual income, psychosocial environment, or material conditions**. *BMJ: British Medical Journal* 2000, **320**(7243):1200.
72. Ploubidis GB, Mathenge W, De Stavola B, Grundy E, Foster A, Kuper H: **Socioeconomic position and later life prevalence of hypertension, diabetes and visual impairment in Nakuru, Kenya**. *International journal of public health* 2013, **58**(1):133-141.
73. Drewnowski A: **The cost of US foods as related to their nutritive value**. *The American journal of clinical nutrition* 2010, **92**(5):1181-1188.
74. Popkin BM: **The nutrition transition and obesity in the developing world**. *The Journal of nutrition* 2001, **131**(3):871S-873S.
75. Sawaya AL, Sesso R, Florencio TM, Fernandes MT, Martins PA: **Association between chronic undernutrition and hypertension**. *Maternal & child nutrition* 2005, **1**(3):155-163.

For peer review only

Table 2: Descriptive characteristics

	Total	n	Urban	n	Rural	p value
Age (years)	22.04 (1.24)	492	22.77 (0.49)	476	21.28 (1.31)	0.001
Weight (kg)	64.62 (14.82)	492	64.67 (15.6)	473	64.55 (14.03)	0.90
Height (m)	1.61 (0.007)	492	1.60 (0.07)	475	1.61 (0.07)	0.001
BMI (kg/m^2)	25.05 (5.59)	492	25.32 (5.91)	476	24.78 (5.24)	0.13
BMI classification (%)		492		476		0.015
Underweight (<18.4 kg/m^2)	5.98		7.10		4.82	
Normal weight (18.5-24.9 kg/m^2)	51.34		46.45		56.39	
Overweight (25-29.9 kg/m^2)	26.19		29.21		23.06	
Obese (>=30 kg/m^2)	16.49		17.24		15.72	
Waist circumference (cm)	80.60 (12.08)	492	80.18 (12.63)	476	81.03 (11.47)	0.26
Central obesity, WC ≥ 80 cm , %	43.81	492	45.70	476	44.74	0.55
Household SES index (sum of assets)	7.24 (2.70)	492	8.83 (2.37)	476	5.59 (1.91)	<0.001
Total MVPA (min/week)*	870(280-1810)	492	420(160-900)	385	1680(970-2580)	<0.001
Sitting time (mins/day)*	300 (240-480)	492	360 (240-480)	385	300 (180-360)	<0.001
Systolic blood pressure	106.68 (11.64)	492	110.30 (11.4)	471	102.89 (10.7)	<0.001
Diastolic blood pressure	70.23 (9.00)	492	72.78 (8.3)	471	67.57 (9.0)	<0.001
BP classification (%)		492		471		<0.001
Low BP	12.46		5.49		19.75	
Normal BP	69.16		67.48		70.91	
Prehypertension	16.20		23.58		8.49	
Hypertensive	2.18		3.46		0.85	
Elevated BP (%)	18.38		27.04		9.34	<0.001
Highest Education attained (%)		480		371		<0.001
Primary school	1.18		0		2.70	
Secondary school	60.75		48.33		76.81	
Tertiary education	38.07		51.67		20.49	

Data presented as mean (SD) otherwise stated; * Median (IQR)

For peer review only

Table 3a: Structural equation model for SES, MVPA and BMI on SBP in urban women

**Effect of: N=489**	**Outcome:**	**Direct effects(95% CI)**	**Indirect effects(95% CI)**	**Total effects(95% CI)**	**Proportion of total effect mediated**
**Household assets**	SBP	-0.34 (-0.75; 0.07)		-0.29 (-0.70; 0.12)	0.13 [a]
	via BMI		0.05 (-0.05; 0.14)		
	BMI	0.13 (-0.09; 0.35)		0.11 (-0.11; 0.33)	0.1 [a]
	via MVPA		-0.014 (-0.05; 0.013)		
	MVPA	-41.71 (-73.48; -9.94)**		-41.71 (-73.48; -9.94)**	
**MVPA**	SBP	-0.0002 (-0.001; 0.001)		-0.0000 (-0.0012; 0.0011)	0.3 [a]
	via BMI		0.0001 (-0.0001; 0.0004)		
**BMI**	SBP	0.37 (0.21; 0.53)***		0.37 (0.21; 0.53)***	

Adjusted for age; * P<0.05; ** P< 0.01; ***P< 0.001; SBP; systolic blood pressure, MVPA; moderate to vigorous intensity physical activity, BMI; body mass index, SES; social economic status, [a] Assessed using the absolute values for both indirect and direct effects

Urban Fit Indices: LR test of model vs. saturated: chi2(4) = 0.97, Prob > chi2 = 0.91; RMSEA = 0.00**;** **CFI= 1.00** Comparative fit index; TLI= 1.12 Tucker-Lewis index; **SRMR=0.011:** Standardized root mean squared residual, CD= 0.017 Coefficient of determination.

For peer review only - http://bmjopen.bmj.com/site/about/guidelines.xhtml

Table 3b: Structural equation model for SES, MVPA and BMI on SBP in rural women

**Effect of:**<br>**N= 378**	**Outcome:**	**Direct effects(95% CI)**	**Indirect effects(95% CI)**	**Total effects(95% CI)**	**Proportion of total effect mediated**
**Household assets**	SBP<br>via BMI	-0.65 (-1.19; -0.096)*	0.08 (-0.04; 0.19)	-0.56 (-1.12; -0.02)*	0.11 [a]
	BMI<br>via MVPA	0.27 (0.01; 0.53)*	-0.01 (-0.04; 0.01)	0.26 (-0.005; 0.53)*	0.04
	MVPA	-29.51 (-87.81; 28.78)		-29.51 (-87.81; 28.78)	
**MVPA**	SBP<br>via BMI	0.0004 (-.0005729 .0013)	0.0001 (-0.0000; 0.0003)	0.0005 (-0.0005; 0.0015)	0.2
**BMI**	SBP	0.33 (0.12; 0.54)**		0.33 (0.12; 0.54)**	

Adjusted for age; * P<0.05; ** P< 0.01; ***P< 0.001; SBP; systolic blood pressure, MVPA; moderate to vigorous physical activity, BMI; body mass index, SES; social economic status, [a] Assessed using the absolute values for both indirect and direct effects

Rural Fit Indices: LR test of model vs. saturated: chi2(4) = 10.51, Prob > chi2 = 0.03; RMSEA = 0.066**; CFI= 0.72** Comparative fit index; TLI= 0.37 Tucker-Lewis index; **SRMR= 0.04 :** Standardized root mean squared residual, CD= 0.03 Coefficient of determination.

For peer review only

Table 3c: Structural equation model for SES, MVPA and BMI on SBP in the pooled sample of urban and rural women

**Effect of: N=867**	**Outcome:**	**Direct effects(95% CI)**	**Indirect effects(95% CI)**	**Total effects(95% CI)**	**Proportion of total effect mediated**
**Household assets**	SBP	0.23 (-0.08; 0.54)		0.46 (0.15; 0.76)**	0.5
	via BMI		0.23 (0.10; 0.35)***		
	BMI	0.20 (0.05; 0.34)**		0.15 (0.01; 0.29)*	0.25 [a]
	via MVPA		-0.05 (-.100; 0.003)		
	MVPA	-144.83 (-170.55; -119.12)***		-144.83 (-170.55; -119.12)***	
**MVPA**	SBP	-0.001 (-0.002; -0.0005)**		-0.001 (-0.002; -0.0003)**	0.1 [a]
	via BMI		0.0001 (-0.0000; 0.0002)		
**BMI**	SBP	0.35 (0.21; 0.49)***		0.35 (0.21; 0.49)***	

Adjusted for age; * P<0.05; ** P< 0.01; ***P< 0.001; SBP; systolic blood pressure, MVPA; moderate to vigorous intensity physical activity, BMI; body mass index, SES; social economic status, [a] Assessed using the absolute values for both indirect and direct effects

Pooled Fit Indices: LR test of model vs. saturated: chi2(4) = 24.829, Prob > chi2 = 0.000; RMSEA = 0.077**;** **CFI= 0.89** Comparative fit index; TLI= 0.75 Tucker-Lewis index; **SRMR=0.033:** Standardized root mean squared residual, CD=0.137 Coefficient of determination.

For peer review only - http://bmjopen.bmj.com/site/about/guidelines.xhtml

Figure legends

Figure 1: Selection of study participants in rural and urban

Figure 2: Structural equation model for SES, MVPA and BMI on SBP in urban

Figure 3: Structural equation model for SES, MVPA and BMI on SBP in rural

Figure 4: Structural equation model for SES, MVPA and BMI on SBP pooled

For peer review only

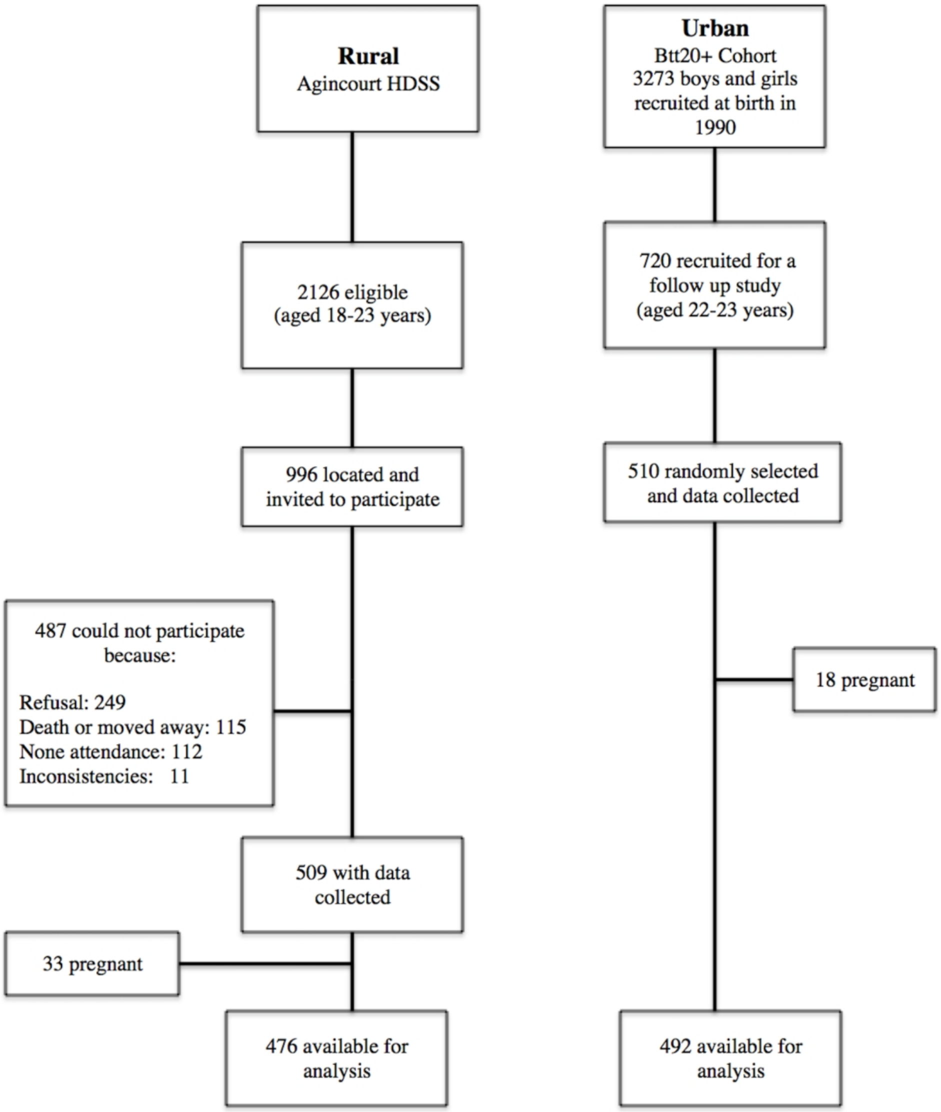

Figure 1: Selection of study participants in rural and urban

144x167mm (300 x 300 DPI)

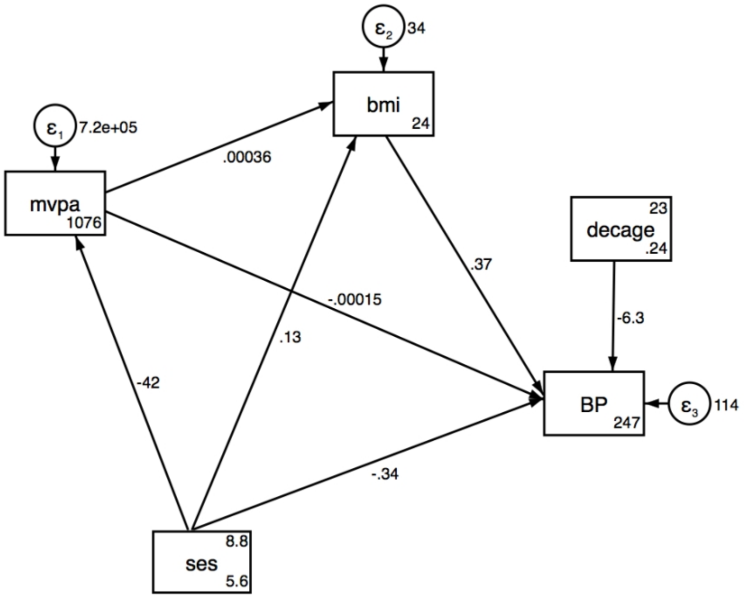

Figure 2: Structural equation model for SES, MVPA and BMI on SBP in urban

106x70mm (300 x 300 DPI)

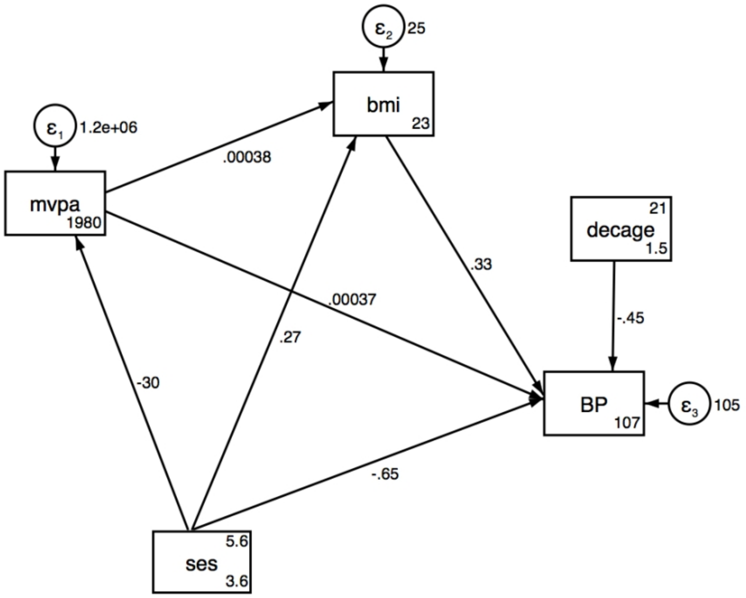

Figure 3: Structural equation model for SES, MVPA and BMI on SBP in rural

106x70mm (300 x 300 DPI)

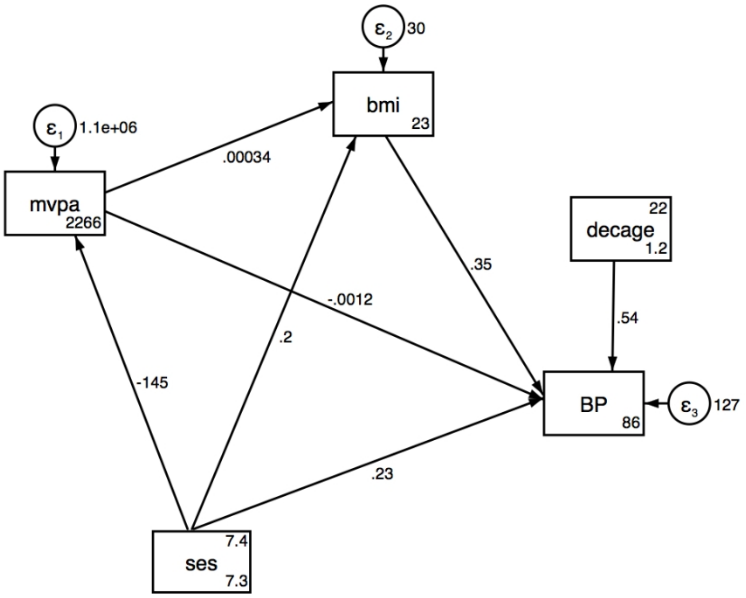

Figure 4: Structural equation model for SES, MVPA and BMI on SBP pooled

106x70mm (300 x 300 DPI)

STROBE Statement—checklist of items that should be included in reports of observational studies

	Item No	Recommendation
**Title and abstract**	1	(*a*) Indicate the study's design with a commonly used term in the title or the abstract **MS Page 4 Par 2**
		(*b*) Provide in the abstract an informative and balanced summary of what was done and what was found **MS Page 4**
**Introduction**		
Background/rationale	2	Explain the scientific background and rationale for the investigation being reported **MS Pages 5-6**
Objectives	3	State specific objectives, including any prespecified hypotheses **MS Page 6 Par 2**
**Methods**		
Study design	4	Present key elements of study design early in the paper **Methods: MS Page 6 Par 3**
Setting	5	Describe the setting, locations, and relevant dates, including periods of recruitment, exposure, follow-up, and data collection **Methods: MS Page 6 Par 3**
Participants	6	(*a*) *Cohort study*—Give the eligibility criteria, and the sources and methods of selection of participants. Describe methods of follow-up **N/A**<br><br>*Case-control study*—Give the eligibility criteria, and the sources and methods of case ascertainment and control selection. Give the rationale for the choice of cases and controls **N/A**<br>*Cross-sectional study*—Give the eligibility criteria, and the sources and methods of selection of participants **Methods: MS Page 6 Par 3**
		(*b*) *Cohort study*—For matched studies, give matching criteria and number of exposed and unexposed **N/A**<br>*Case-control study*—For matched studies, give matching criteria and the number of controls per case **N/A**
Variables	7	Clearly define all outcomes, exposures, predictors, potential confounders, and effect modifiers. Give diagnostic criteria, if applicable **Methods: MS Page 6 Par 4 – Page 8 Par 1**
Data sources/ measurement	8*	For each variable of interest, give sources of data and details of methods of assessment (measurement). Describe comparability of assessment methods if there is more than one group **Methods: MS Page 6 Par 4 – Page 8 Par 1**
Bias	9	Describe any efforts to address potential sources of bias **Methods: MS Page 6 Par 3**
Study size	10	Explain how the study size was arrived at **Methods: MS Page 6 Par 3**
Quantitative variables	11	Explain how quantitative variables were handled in the analyses. If applicable, describe which groupings were chosen and why **Methods: MS Page 8 Par 2**
Statistical methods	12	(*a*) Describe all statistical methods, including those used to control for confounding **Statistical analyses: MS Page 8 Par 2 and 3**
		(*b*) Describe any methods used to examine subgroups and interactions **N/A**
		(*c*) Explain how missing data were addressed **Statistical analyses: MS Page 8 Par 2**
		(*d*) *Cohort study*—If applicable, explain how loss to follow-up was addressed<br>*Case-control study*—If applicable, explain how matching of cases and controls was

For peer review only - http://bmjopen.bmj.com/site/about/guidelines.xhtml

addressed **N/A**

Cross-sectional study—If applicable, describe analytical methods taking account of sampling strategy **N/A**

(*e*) Describe any sensitivity analyses **N/A**

Continued on next page

For peer review only

For peer review only - http://bmjopen.bmj.com/site/about/guidelines.xhtml

Results		
Participants	13*	(a) Report numbers of individuals at each stage of study—eg numbers potentially eligible, examined for eligibility, confirmed eligible, included in the study, completing follow-up, and analysed **Results: Page 8 Par 5 – Page 9 Par 1**
		(b) Give reasons for non-participation at each stage **N/A**
		(c) Consider use of a flow diagram **N/A**
Descriptive data	14*	(a) Give characteristics of study participants (eg demographic, clinical, social) and information on exposures and potential confounders **Page 8 Par 5 – Page 9 Par 1**
		(b) Indicate number of participants with missing data for each variable of interest **Results: Page 8 Par 5**
		(c) *Cohort study*—Summarise follow-up time (eg, average and total amount**) N/A**
Outcome data	15*	*Cohort study*—Report numbers of outcome events or summary measures over time **N/A**
		Case-control study—Report numbers in each exposure category, or summary measures of exposure **N/A**
		Cross-sectional study—Report numbers of outcome events or summary measures **Results: Page 8 Par 5**
Main results	16	(*a*) Give unadjusted estimates and, if applicable, confounder-adjusted estimates and their precision (eg, 95% confidence interval). Make clear which confounders were adjusted for and why they were included **Results: Page 9 Par 1**
		(*b*) Report category boundaries when continuous variables were categorized **N/A**
		(*c*) If relevant, consider translating estimates of relative risk into absolute risk for a meaningful time period **N/A**
Other analyses	17	Report other analyses done—eg analyses of subgroups and interactions, and sensitivity analyses **N/A**
**Discussion**		
Key results	18	Summarise key results with reference to study objectives **Discussion: Page 9 Par 2**
Limitations	19	Discuss limitations of the study, taking into account sources of potential bias or imprecision. Discuss both direction and magnitude of any potential bias **Discussion: Page 11 Par 4**
Interpretation	20	Give a cautious overall interpretation of results considering objectives, limitations, multiplicity of analyses, results from similar studies, and other relevant evidence **Page 9 Par 3 – Page 11 Par 3**
Generalisability	21	Discuss the generalisability (external validity) of the study results **Discussion: Page 9 Par 3 – Page 11**
**Other information**		
Funding	22	Give the source of funding and the role of the funders for the present study and, if applicable, for the original study on which the present article is based **Funding: Page 12 Par 1**

*Give information separately for cases and controls in case-control studies and, if applicable, for exposed and unexposed groups in cohort and cross-sectional studies.

Note: An Explanation and Elaboration article discusses each checklist item and gives methodological background and published examples of transparent reporting. The STROBE checklist is best used in conjunction with this article (freely available on the Web sites of PLoS Medicine at http://www.plosmedicine.org/, Annals of Internal Medicine at http://www.annals.org/, and Epidemiology at http://www.epidem.com/). Information on the STROBE Initiative is available at www.strobe-statement.org.

For peer review only - http://bmjopen.bmj.com/site/about/guidelines.xhtml

BMJ Open

Body composition and physical activity as mediators in the relationship between socio-economic status and blood pressure in young South African women: A structural equation model analysis

Journal:	*BMJ Open*
Manuscript ID	bmjopen-2018-023404.R2
Article Type:	Research
Date Submitted by the Author:	08-Nov-2018
Complete List of Authors:	Munthali, Richard; University of the Witwatersrand, MRC/Wits Developmental Pathways for Health Research Unit<br>Manyema, Mercy; Wits University, Epidemiology and Biostatistics<br>Said-Mohamed, Rihlat; University of the Witwatersrand, MRC/Wits Developmental Pathways for Health<br>Kagura, Juliana; University of Witwatersrand, Paediatrics and Child health<br>Tollman, Stephen; University of the Witwatersrand,<br>Kahn, Kathleen; University of the Witwatersrand,<br>Gómez-Olivé, F. Xavier; University of the Witwatersrand, Medical Research Council/Wits Rural Health and Health Transitions Unit (Agincourt), School of Public Health, Faculty of Health Sciences<br>Micklesfield, Lisa; University of the Witwatersrand, MRC/Wits Developmental Pathways for Health Research Unit<br>Dunger, David; University of Cambridge, Paediatrics<br>Norris, Shane; University of Witwatersrand, Paediatrics and Child Health
**Primary Subject Heading**:	Epidemiology
Secondary Subject Heading:	Public health, Epidemiology
Keywords:	Hypertension < CARDIOLOGY, Obesity, Socioeconomic status, Physical activity, Structural equation model, Body mass index

SCHOLARONE™
Manuscripts

Body composition and physical activity as mediators in the relationship between socio-economic status and blood pressure in young South African women: A structural equation model analysis

Richard J Munthali[1], Mercy Manyema[1, 2], Rihlat Said-Mohamed[1], Juliana Kagura[1], Stephen Tollman [3,4,5], Kathleen Kahn[3, 4,5], F. Xavier Gómez-Olivé[3], Lisa K. Micklesfield[1], David Dunger [6,1], Shane A. Norris[1]

Affiliations:

[1]MRC/WITS Developmental Pathways for Health Research Unit, Department of Paediatrics, School of Clinical Medicine, Faculty of Health Sciences, University of the Witwatersrand, 7 York Rd, Parktown 2193, Johannesburg, South Africa

[2]DST-NRF Centre of Excellence in Human Development, University of the Witwatersrand, Johannesburg, South Africa

[3]MRC/Wits Rural Public Health and Health Transitions Research Unit, School of Public Health, Faculty of Health Sciences, University of the Witwatersrand, Johannesburg, South Africa

[4] INDEPTH Network, Accra, Ghana

[5]Umeå Centre for Global Health Research, Sweden.

[6]Department of Paediatrics, MRL Wellcome Trust-MRC Institute of Metabolic Science, NIHR Cambridge Comprehensive Biomedical Research Centre, University of Cambridge, Box 116, Addenbrooke's Hospital, Hills Road, Cambridge CB2 0QQ, UK

Conflict of interest: The views expressed in the submitted article are our own and not an official position of the affiliated institutions or funder. Authors have no financial relationships relevant to this article to disclose.

For peer review only

Corresponding Author: Dr. Richard J. Munthali

MRC/Wits Developmental Pathways for Health Research Unit

University of the Witwatersrand

Johannesburg

Tel: +27119331122

Email: munthali@aims.ac.za

Authors' emails:

Richard Junganiko Munthali: munthali@aims.ac.za

Mercy Manyema: mercy.manyema@gmail.com

Rihlat Said-Mohamed: rihlat.saidmohamed@wits.ac.za

Juliana Kagura: julianakagura@gmail.com

Stephen Tollman: stephen.tollman@wits.ac.za

Kathleen Kahn: kathleen.kahn@wits.ac.za

F. Xavier Gómez-Olivé: F.Gomez-OliveCasas@wits.ac.za

Lisa K. Micklesfield: lisa.micklesfield@wits.ac.za

David Dunger: dbd25@cam.ac.uk

Shane A Norris: shane.norris@wits.ac.za

For peer review only

For peer review only

Abstract

For peer review only - http://bmjopen.bmj.com/site/about/guidelines.xhtml

Objectives Varying hypertension prevalence across different socio-economic strata within a population has been well reported. However the causal factors and pathways across different settings are less clear, especially in sub-Saharan Africa. Therefore, this study aimed to compare blood pressure (BP) levels, and investigate the extent to which socioeconomic status (SES) is associated with blood pressure, in rural and urban South Africa women.

Setting Rural and urban South Africa.

Design Cross-sectional.

Participants Cross-sectional data on SES, total moderate-vigorous physical activity (MVPA), anthropometric and blood pressure were collected on rural (n=509) and urban (n=510) young black women (18-23 years age). Pregnant and mentally or physically disabled women were excluded from the study.

Results The prevalence of combined overweight and obesity (46.5% versus 38.8%) and elevated BP (27.0% versus 9.3%) were higher in urban than rural women respectively. Results from the structural equation modelling showed significant direct positive effects of body mass index (BMI) on systolic BP (SBP) in rural, urban and pooled datasets. Negative direct effects of SES on SBP and positive total effects of SES on SBP were observed in the rural and pooled datasets respectively. In rural young women, SES had direct positive effects on BMI and was negatively associated with MVPA in urban and pooled analyses. BMI mediated the positive total effects association between SES and SBP in pooled analyses (ß; 95%CI, 0.46; 0.15 to 0.76).

Conclusions Though South Africa is undergoing nutritional and epidemiological transitions; the prevalence of elevated BP still varies between rural and urban young women. The association

For peer review only

between SES and SBP varies considerably in economically diverse populations with BMI being the most significant mediator. There is a need to tailor prevention strategies to take into account optimizing BMI when designing strategies to reduce future risk of hypertension in young women.

Keywords Blood pressure, Body mass index, Hypertension, Obesity, Urban, Rural, Socioeconomic status, Structural equation model, Physical activity

For peer review only

Strengths

1. The use of structural equation modelling allowed us to explore direct and indirect (mediation) effects of social economic status, physical activity and body mass index on elevated blood pressure from a representative sample of rural and urban populations of South African young women.
2. Although the urban and rural cohorts were from two different studies, the same research unit conducted both studies and, therefore, the data collection and management process were consistent between the two sites, thereby allowing for accurate comparison.

Limitations

1. Other unmeasured data, such as undernutrition in infancy, and dietary patterns were not included in the current analyses.
2. The low reliability of self-report data on physical activity could introduce bias. Thus, there is need for more accurate, objective measures of physical activity

For peer review only

Introduction

High blood pressure (BP) or hypertension is a leading risk factor accounting for 7% of global disability-adjusted life years (DALYs) and contributing to the 34.5 million non-communicable disease (NCD) related deaths in 2010 [1, 2]. A recent global meta-analysis, involving 19.1 million individuals, reported that on average there has been a decrease in BP globally, but low- to middle-income countries (LMICs) have seen an increase in hypertension [3]. The prevalence of high BP in LMICs is estimated at 30% [4, 5] and it is the most significant risk factor for cardiovascular disease, most notably stroke [6]. In 2000, hypertension was estimated to have caused 9% of all deaths and over 390 000 DALYs in South Africa. Further, hypertension contributed to 50% of all strokes and 42% of ischaemic heart disease (IHD), signifying a substantial public health burden [7]. A systematic review of sub-Saharan African (SSA) data shows prevalence rates of hypertension of up to 41% with higher prevalence rates noted in urban compared to rural populations [8, 9]. A study in men and women aged 40 to 60 years of age in six sites across four SSA countries, including South Africa, showed the same trend with South African urban and rural cohorts having the highest prevalence of hypertension (41.6 to 54.1%) [10].

LMICs are experiencing both epidemiological and nutritional transitions with urban populations further along the transition as demonstrated by the higher prevalence of obesity and NCDs [4, 5, 8, 10-15]. Some evidence has shown that there are differences in the levels of BP between rural and urban settings [8], while other studies have found no significant differences [16].

For peer review only

According to Glass and McAtee, internal biological systems are sculpted by an interaction between genes and prolonged exposure to particular external environments, a principle they call embodiment [17]. Thus the differences in built and social environments between rural and urban settings may explain the differences in disease prevalence. A Ghanaian study showed that both systolic blood pressure (SBP) and diastolic blood pressure (DBP) were significantly lower in rural participants compared to urban participants [18]. However, a similar study in adolescents found that BP levels were only lower in rural boys, with no difference in the girls [19]. Pediatric and adolescent hypertension have been reported to track into adulthood in a South African urban population [20]. Results on elevated BP from studies in rural South African children have reported prevalence rates varying from 1.0% to 25.4% [21-24]. The factors explaining these differences have not been fully studied in LMICs.

Socioeconomic factors such as education, household income and household assets have been associated with BP levels [25-27]. In a US cohort of young adults, a higher household income remained associated with lower SBP even after controlling for all potential covariates including age, sex and bio-behavioral factors [28]. Similarly, in a French sample of 30-79 year olds, SBP independently increased and was inversely associated with both individual education and residential neighborhood education [29]. Studies in African countries have also found varying associations between socioeconomic status (SES) and BP patterns, with both positive and negative associations reported [8, 30, 31]. Some studies have speculated that the association between SES and body mass index (BMI), physical activity levels, diet, smoking, alcohol intake and malnutrition may influence BP patterns [18, 28, 31, 32]. Physical activity has been inversely associated with blood pressure and BMI directly associated with BP in more advanced economies, but inconsistent associations have been reported in LMICs [25, 33-37].

For peer review only

There is a need to examine BP and its determinants in young South African adults given the high rates of overweight and obesity, and hypertension observed in this age group [20, 38]. Recent South African reports also indicate that the highest pregnancy rates occur in the age range of 20-24 years, with 26.2% of births reported, followed closely by the 25-29 year age group (25.7%) [39], and therefore targeting young adult women would also reduce adverse health outcomes in their children. It is important to closely examine rural-urban differences in hypertension due to differences in the epidemiology of obesity and SES divergence in the South African context, in order to better suit interventions to the different settings [23, 26, 30, 40-43]. Therefore, this study aims to compare BP levels between rural and urban young adult South African women, and to determine whether there is an association between SES and BP, and whether it is mediated by physical activity and BMI.

Methods

Study sample and site

The rural Agincourt site, 2016 potential female participants between the ages of 18 and 23 years were in the existing Agincourt Health and Socio-demographic Surveillance System database [44]. Only 996 were located during the data collection period and were invited to participate and of these, 509 female participants were recruited. The urban sample consisted of 510 young women between the ages of 22 and 23 years who were randomly selected from the sample of 720 females who were part of the Birth-to-Twenty plus (BT20+) Young Adult Survey [45, 46]. Young women (n=51; 33 in rural and 18 in urban) who were pregnant at the time of the study were excluded, see the study design flow chart in **Figure 1**. Measurements and questionnaires were completed by trained research assistants and nurses, and were standardised between both sites, to eliminate biases. The study protocols were approved by the

For peer review only

Human Research Ethics Committee of the University of the Witwatersrand (Clearance certificates M120138 for the Ntshembo-Hope Cross Sectional Survey in Agincourt and M111182 for the BT20+ survey). Written consent to participate was provided by participants, and mentally or physically disabled women were excluded from the study.

Patient and Public Involvement

No patients private or public were involved in this study, as it was a community population based.

Blood pressure

Blood pressure (mm Hg) was the outcome variable and it was measured using an Omron 6 automated machine (Kyoto, Japan). A five minute seated rest was observed before taking the BP measurements. Participants' seated BP was measured three times on the right side, with a 2-minute interval between each measurement. The mean for the second and third readings was recorded for the current analysis. We had various cuff sizes and the appropriate size was used to accommodate differences in arm circumference.

According to the Seventh Report of the Joint National Committee on Prevention, Detection, Evaluation, and Treatment of High Blood Pressure [47], five categories of BP have been established for adults 18 years of age and older as shown in Table 1. These cut-offs were utilized in the current study. Prehypertension and hypertension were combined to create a new variable called elevated BP.

Table 1: Blood pressure classification [47]

For peer review only - http://bmjopen.bmj.com/site/about/guidelines.xhtml

Classification	Systolic Blood Pressure		Diastolic Blood Pressure
Low	<90	or	< 60
Normal	<120	and	<80
Prehypertension	120-139	or	80-90
High: Stage 1 Hypertension	140-159	or	90-99
High: Stage 2 hypertension	≥160	or	≥100

For peer review only

SBP was used in structural equation models (SEM) as it is more relevant in adults, and a good predictor of adverse health outcomes later in life [48], such as CVDs.

Anthropometry

At both sites, participants' height and weight were measured by trained research assistants using standard techniques [49, 50] . Weight was measured in light clothing and barefoot to the

nearest 0.1 kg using a digital scale (Tanita model TBF-410; Arlinghton Heights; USA). Height was measured barefoot to the nearest 0.1 cm using a stadiometer (Holtain, Crymych, UK). Waist circumference was measured with a non-stretchable fibreglass tape at the level of the umbilicus. BMI was calculated as weight/height^2 (kg/m^2).

Socio-economic status (SES)

Physical assets owned in the participants' household were used as a proxy for SES index [51]. It was generated by summing the number of assets owned in the household from the following: television, car, washing machine, fridge, phone, radio, microwave, cell phone, DVD/Video, DSTV (cable channel), computer, internet, medical aid. Previous studies in this population have shown that the sum of physical assets (household assets) is closely related to the household per capital expenditure and household income [51-53]. The household SES is regarded as a good measure of accumulated household wealth so it is a more reflective wealth index than income of a household's wealth over time.

Physical activity

The Global Physical Activity Questionnaire (GPAQ), developed for global physical activity surveillance, was completed via interview to obtain self-reported physical activity [54]. Total MVPA in minutes per week (mins/wk) was calculated by adding occupation, travel-related and leisure time moderate and vigorous intensity physical activity. Sitting time (mins/wk) was used as a proxy for sedentary time.

Statistical analyses

Analysis of variance and student's t test, and Chi-squared tests and Wilcoxon rank sum test for non-parametric variables, were conducted to compare study characteristics between urban and rural young women. Structural equation modeling (SEM), was used to test and estimate the

For peer review only - http://bmjopen.bmj.com/site/about/guidelines.xhtml

direct and indirect associations between variables, most especially the mediation roles of physical activity (MVPA) or sedentary time (sitting), and body composition (BMI and WC), in the association between SES and SBP. SEMs allow us to assess the mediation effects of multiple mediators [55]. SEM decomposed SES-BP associations into two parts, direct (unmediated) and indirect (mediated through MVPA/sitting and BMI/WC).

Direct, indirect and total effects were computed and recorded, and the proportion of the total effect mediated was calculated. To evaluate the best fitting model for our data, we calculated different goodness of fit indices including Chi-squared test, Root mean squared error of approximation (RMSEA), Comparative fit index (CFI), Tucker-Lewis index (TLI) and Standardized root mean squared residual (SRMR) [56]. Though the Chi-squared test has been popularly used as a goodness of fit index, it has been reported to be biased and not reliable as the only goodness of fit index. It is also highly sensitive to sample size [57, 58] , and often inflated with non-normal data such as physical activity data and we therefore employed the Hu and Bentler's Two-Index Presentation Strategy (1999) combination rule, with cut off values depending on the fitness index, to determine the best model fit [56, 59]. We estimated the coefficients (β) with 95% confidence intervals (95% CI) for the direct, indirect and total effects and also calculated the proportion of association mediated by indirect effects. If the direct and indirect effects had opposite signs (negative or positive effects) the proportion mediated was assessed using the absolute values for all indirect and direct effects [60].

All the analyses were conducted using STATA (version 13.0; STATA Corp., College Station, TX, USA). We confirmed SEM results by running the SEM with the Satorra-Bentler and Huber-White (Robust) Sandwich Estimator options [61] in STATA (version 15.1; STATA Corp., College Station, TX, USA). These options relax the normality assumption hence are robust to non-normal data, which would be the case for mvpa and SES in the current study. A P-value < 0.05 was considered statistically significant.

For peer review only - http://bmjopen.bmj.com/site/about/guidelines.xhtml

Results

Study characteristics

Descriptive statistics for the non-pregnant study participants (urban, n=492; rural, n=476) are presented in Table 2. There was no difference in BMI or waist circumference between the urban and rural participants, but the prevalence of overweight and obesity was significantly higher in the urban (46.5%) compared to the rural young women (38.8%). Household SES was significantly higher in the urban compared to the rural group. Self-reported MVPA was significantly higher in the rural than urban women ($p<0.001$), and the urban women spent significantly more time sitting than their rural counterparts ($p<0.001$). Systolic and diastolic BP were significantly higher in the urban group, as was the prevalence of elevated BP (27.0 vs. 9.3%).

Structural equation models for body mass index and waist circumference

Results from the SEMs for SES associations with SBP via MVPA and BMI are presented in Tables 3a, 3b and 3c for urban, rural and pooled analyses respectively, and also shown in Figures 1, 2, 3. No significant direct or indirect effects via (MVPA or BMI) of SES on SBP were observed in the urban women, but there were significant direct effects of SES on MVPA. Results showed that individuals with a higher SES index were less likely to be physically active in pooled data and urban women. In rural women, a one-unit increase in total household assets was associated with a decrease of 0.65 mmHg (95% CI: -1.19 to -0.10) in SBP and an increase of 0.27 kg/m^2 in BMI (95% CI: 0.1 to 0.53) (**Tables 3a, 3b and Figures 2, 3)**. The

For peer review only

332 SEM for the pooled sample showed a significant indirect effect of household SES on SBP via
333 BMI, with 50% of the total effect being mediated by BMI **(Table 3c and Figure 4).** Direct
334 positive effects of BMI on SBP were observed in both settings and the pooled sample with a 1
335 kg/m^2 increase in BMI being associated with an increase of 0.37 mmHg (95% CI: 0.21 to 0.53)
336 and 0.33 (95% CI: 0.12 to 0.54) mmHg SBP in urban and rural young women, respectively.
337 Similar results were observed when including waist circumference as the body composition
338 indicator as shown in the SEM path diagrams with estimates in Figure S1 (supplementary
339 data). The results from the SEMs with the Satorra-Bentler adjustment option, accounting for
340 non-normality of the exposure, are shown in Figure S2 (supplementary data).

341 **Discussion**

342 A rising prevalence of hypertension has been reported in South Africa. Peer and colleagues
343 reported a higher prevalence in 2008 (35.6%) compared to 1990 (21.6 %) in men and women
344 aged 25-74 years in an urban black community in Cape Town, South Africa [40]. We have
345 shown in young adult women from urban and rural South Africa, an overall elevated BP
346 prevalence of 18.4 % (27.0 % in urban and 9.3 % in rural). We have also shown a direct
347 effect of BMI on SBP in the urban and rural women separately, as well as when pooled,
348 thereby providing further evidence of an association between overall adiposity and blood
349 pressure. The total effects of SES on SBP were the same in both settings.

350 Prevalence data on elevated BP and hypertension from other countries in SSA have shown
351 conflicting results when comparing urban and rural communities. In Malawi, a higher prevalence
352 of hypertension in urban compared to rural communities has been reported and attributed to
353 differences in lifestyle as rural communities participate in subsistence based agricultural
354 activities while the urban community has a more westernized lifestyle with higher salt intake
355 and lower physical inactivity [9]. Similarly, data from Ghana have shown a higher mean SBP

For peer review only - http://bmjopen.bmj.com/site/about/guidelines.xhtml

and DBP and a higher prevalence of hypertension in urban communities [18, 62]. In the PURE study in South Africa, Pisa and colleagues reported that both urban adult men and women had higher mean blood pressures in comparison to their rural peers though the overall CVD risk factors were equally prevalent in both settings [41]. In contrast, findings from Cameroon have reported a higher BP prevalence in rural compared to urban men and women older than 40 years old, while Kenyan studies have reported no significant differences [16, 63]. Results from six urban and rural sites in four SSA countries – Kenya, South Africa, Ghana and Burkina Faso – have reported a prevalence of hypertension in women aged between 40 and 60 years ranging from 15.1% in rural Burkina Faso to 54.1% in urban South Africa [10]. It was also reported that in all three South African sites, both rural and urban, the prevalence of hypertension was higher than in the other three countries [10]. These findings show the complex health transitions occurring in SSA and the impact that this is having on cardio-metabolic disease risk.

Our study showed significant differences in SES between the urban and rural samples, as well a big variation in SES within these two settings. The social patterning of CVD risk factors, including hypertension, in SSA and LMICs has in part been attributed to differences in countries' socioeconomic development. Previous results from five countries, (two high income and three LMICs), reported that hypertension and other CVD risk factors were substantially associated with education and wealth status; individuals with less education and lower wealth generally showing higher prevalence of CVD risk factors [64] . The effect of SES in this study is most evident in the rural women for whom household SES was lower (compared to urban) and who may be transitioning faster (both nutritionally and economically) than the urban young women. Though SES is positively associated with BMI in rural young women, it is negatively associated with SBP. There may be other factors, such as physical activity due to agricultural activities or dietary patterns, which were not recorded. In addition, the weight gain observed

For peer review only

might not be due to fat mass, which has been reported to be positively associated with SBP before [65], but rather to muscle mass and bone mass.

In Mexico, women in rural and upper SES categories were likely to have a higher SBP, while we have reported that a higher SES was associated with a decrease in SBP in rural communities. At a population level, there is a need to consider different SES categories and monitor the effects of transitioning from one SES category to another on hypertension, since these categories may respond differently to an increase or a decrease in their SES. Kagura and colleagues tracked SES in South African children and reported that moving from the low SES in infancy to a higher SES in adolescence had a protective effect on SBP level in young adulthood [26]. Our results have shown that this could be more pronounced in rural areas.

We observed a positive association between SES and BMI in the rural sample and the same direction of effects was observed in the urban, though not significant. This is in line with results reported in many LMICs including South Africa, but in contrast with those reported in higher income populations [33, 34, 64]. A systematic review of studies between 1989 to 2007 reported that SES was positively associated with obesity in the middle transitioning economies such as South Africa and Jamaica [66]. We have shown that both in the rural and urban participants (not significant), a higher SES resulted in reduced SBP, while the pooled analysis showed a positive total effect association between SES and SBP. This could be due to the introduction of more variation in SES when data from both sites are pooled; with many individuals with low SES in the rural area, the associations became skewed towards the low SES individuals. This may suggest that different transitional levels of SES have different effects on hypertension risk depending on the environment (either urban or rural). Though not significant, the total effects of SES on SBP are the same in both rural and urban hence the differences in prevalence cannot be explained by the setting or SES alone. In urban and rural settings of four countries (Kenya, Namibia, Nigeria and Tanzania), the prevalence of age

For peer review only - http://bmjopen.bmj.com/site/about/guidelines.xhtml

standardized hypertension was similarly high and ranging from 19.3 % to 38.0 % [11]. Cois and colleagues reported that a higher SES was associated with lower SBP in a nationally representative sample of South African women [25] using SEM models. Alcohol use, PA, smoking and resting heart rate and BMI were reported to be the mediators of the indirect of the association between SES and SBP in men but not in women, suggesting that other factors may play a major role in women [25]. Similarly, our results show that neither PA nor BMI mediate the association between SES and SBP in urban and rural settings in isolation, suggesting that other factors may explain the association. Among those, dietary patterns and stress have been reported to be independently associated with SBP [67, 68].

The significant direct associations between BMI and SBP are in line with other findings in South Africa and within the SSA region [11, 33, 40, 42, 69, 70]. This link was consistent in rural, urban and pooled data sets, indicating the importance of BMI in the aetiology of high BP. Munthali and colleagues reported that the link between obesity and hypertension could be observed as early as five years of age. Children with early onset of obesity were at higher risk of developing hypertension in late adolescence [38].

In this study, using SEM models to explore the mediation role of BMI and PA helped quantify potential contributions of these variables to the effect of SES on SBP. The results show that PA was not a significant mediator in the association between SES and BP in the urban or the rural samples. SES was negatively associated with MVPA in urban and pooled samples, indicating that as individuals transition from low to higher SES, they reduce their physical activity level. We speculate that these differences in the association between SES and SBP in both our rural and urban results, and in those from high-income countries are due to differences in levels of nutritional and epidemiological transition in these regions [71, 72]. Those with low SES in high-income countries are likely to consume cheaper, more energy dense foods, participate in less leisure time PA and be more sedentary [73, 74]. In LMICs,

For peer review only

agricultural activities remain a part of everyday life and a day-to-day activity in rural living, while those with higher SES in the same settings rapidly adopt the westernized life style with less PA, fewer agricultural activities and home grown food. However, this speculation is not supported by the data on PA in this study despite the rural participants having a higher PA. Our understanding of the Agincourt rural economy is that agriculture is quite a minor aspect though very useful to augment the household income.

The limitations of this study are that other unmeasured data, such as undernutrition in infancy, which is a known risk factor for high BP later in life [75], and dietary patterns were not included in the current analyses. We are currently working on research to address this limitation. We can also not rule out the role of genetics. Secondly, the low reliability of self-report data on PA could introduce bias. Thus, there is need for more precise, objective measures of physical activity to strengthen the results of our analysis. Lastly, longitudinal data, especially as the socioeconomic environment is changing rapidly due to rural-urban labor migration and other factors would be helpful to examine these associations over time. The cross-sectional design lacks a temporal component between the factors analyzed. Thus, it is difficult to say anything certain about the direction of the associations, hence the need for the longitudinal data.

Conclusions

Though the prevalence of overweight or obesity is relatively higher in both rural and urban than those reported in other SSA countries, women in the urban setting were at more risk for elevated blood pressure than their rural counterparts. The link between SES and SBP varies in a more economically diverse population, as seen with the pooled rural and urban dataset, with BMI being the most likely mediator. There is need to consider optimizing BMI as a key

For peer review only

intervention strategy in young adults in part to combat hypertension. Our findings should be replicated with prospective data.

Funding

Norris is supported by the UK MRC DfID African Research Leader Scheme and by the DST-NRF Centre of Excellence in Human Development at the University of the Witwatersrand, Johannesburg. Birth to Twenty data collection was supported by the Wellcome Trust under Grant [092097/Z/10/Z]. The MRC/Wits- Agincourt Unit is supported by the South African Medical Research Council, and the Wellcome Trust under Grants [058893/Z/99/A, 069683/Z/02/Z, 085477/Z/08/Z, 085477/B/08/Z]. Opinions expressed and conclusions arrived at, are those of the authors and are not to be attributed to the CoE in Human Development.

Conflict of interest

Authors have no conflicts of interest to disclose.

Consent for publication

Not applicable

Data Sharing

The datasets used and/or analysed during the current study are available from the Developmental Pathways

for Health Research Unit data management department by contacting Prof. Shane A Norris on reasonable request

Competing interest

The authors declare that they have no competing interests

Authors' contributions

For peer review only

RJM and SAN conceptualized the manuscript. RJM analyzed the data. RJM MM RSM JK ST KK FXG LKM DD SAN interpreted the data. RJM wrote the manuscript and all authors were involved in editing and approving the final manuscript.

Acknowledgements

We wish to thank the Bt20+ and Agincourt participants for taking part in the study and the Bt20+ and Agincourt

team for their relentless support throughout the study.

Ethics Approval and Consent to Participate

Prior to the study, the study protocols were approved by the Human Research Ethics Committee of the University of the Witwatersrand (Clearance certificates M120138 for the Ntshembo-Hope Cross Sectional Survey in Agincourt and M111182 for the BT20+ survey). Independent written informed consent to participate was obtained from participants.

For peer review only

References

1. Lim SS, Vos T, Flaxman AD, Danaei G, Shibuya K, Adair-Rohani H, AlMazroa MA, Amann M, Anderson HR, Andrews KG *et al*: **A comparative risk assessment of burden of disease and injury attributable to 67 risk factors and risk factor clusters in 21 regions, 1990–2010: a systematic analysis for the Global Burden of Disease Study 2010**. *The Lancet* 2012, **380**(9859):2224-2260.
2. Lozano R, Naghavi M, Foreman K, Lim S, Shibuya K, Aboyans V, Abraham J, Adair T, Aggarwal R, Ahn SY: **Global and regional mortality from 235 causes of death for 20 age groups in 1990 and 2010: a systematic analysis for the Global Burden of Disease Study 2010**. *The Lancet* 2013, **380**(9859):2095-2128.
3. Ezzati M, Geleijnse J: **Worldwide trends in blood pressure from 1975 to 2015**. *The Lancet* 2017, **389**(10064):37-55.
4. Sarki AM, Nduka CU, Stranges S, Kandala N-B, Uthman OA: **Prevalence of Hypertension in Low- and Middle-Income Countries: A Systematic Review and Meta-Analysis**. *Medicine* 2015, **94**(50):e1959.
5. Chow CK, Teo KK, Rangarajan S, Islam S, Gupta R, Avezum A, Bahonar A, Chifamba J, Dagenais G, Diaz R: **Prevalence, awareness, treatment, and control of hypertension in rural and urban communities in high-, middle-, and low-income countries**. *Jama* 2013, **310**(9):959-968.
6. Kearney PM, Whelton M, Reynolds K, Muntner P, Whelton PK, He J: **Global burden of hypertension: analysis of worldwide data**. *The lancet* 2005, **365**(9455):217-223.
7. Norman R, Gaziano T, Laubscher R, Steyn K, Bradshaw D, Collaboration SACRA: **Estimating the burden of disease attributable to high blood pressure in South Africa in 2000**. *South African Medical Journal* 2007, **97**(8):692-698.
8. Addo J, Smeeth L, Leon DA: **Hypertension in Sub-Saharan Africa a systematic review**. *Hypertension* 2007, **50**(6):1012-1018.
9. Simmons D, Barbour G, Congleton J, Levy J, Meacher P, Saul H, Sowerby T: **Blood pressure and salt intake in Malawi: an urban rural study**. *Journal of epidemiology and community health* 1986, **40**(2):188-192.
10. Gómez-Olivé FX, Ali SA, Made F, Kyobutungi C, Nonterah E, Micklesfield L, Alberts M, Boua R, Hazelhurst S, Debpuur C: **Stark Regional and Sex Differences in the Prevalence and Awareness of Hypertension: An H3Africa AWI-Gen Study Across 6 Sites in Sub-Saharan Africa**. *Global Heart* 2017.

For peer review only

For peer review only - http://bmjopen.bmj.com/site/about/guidelines.xhtml

11. Hendriks ME, Wit FW, Roos MT, Brewster LM, Akande TM, de Beer IH, Mfinanga SG, Kahwa AM, Gatongi P, Van Rooy G: **Hypertension in sub-Saharan Africa: cross-sectional surveys in four rural and urban communities.** *PloS one* 2012, **7**(3):e32638.
12. Lemogoum D, Seedat YK, Mabadeje AF, Mendis S, Bovet P, Onwubere B: **International Forum for Hypertension control and prevention in Africa. Recommendations for prevention, diagnosis and management of hypertension and cardiovascular risk factors in sub-Saharan Africa**. *J Hypertens* 2003, **21**.
13. Schutte A, Botha S, Fourie C, Gafane-Matemane L, Kruger R, Lammertyn L, Malan L, Mels C, Schutte R, Smith W: **Recent advances in understanding hypertension development in sub-Saharan Africa**. *Journal of Human Hypertension* 2017.
14. Thorogood M, Connor MD, Lewando Hundt G, Tollman SM: **Understanding and managing hypertension in an African sub-district: A multidisciplinary approach1**. *Scandinavian Journal of Public Health* 2007, **35**(69 suppl):52-59.
15. Yusufali A, Khatib R, Islam S, AlHabib K, Kelishadi R, Rangarajan S, Yusuf S: **LBOS 03-01 PREVALENCE, AWARENESS, TREATMENT, AND CONTROL OF HYPERTENSION IN THE MIDDLE EAST: RESULTS FROM THE PROSPECTIVE URBAN RURAL EPIDEMIOLOGY (PURE) STUDY**. *J Hypertens* 2016, **34 Suppl 1**:e551.
16. Poulter N, Khaw K, Hopwood B, Mugambi M, Peart W, Rose G, Sever P: **Blood pressure and its correlates in an African tribe in urban and rural environments**. *Journal of epidemiology and community health* 1984, **38**(3):181-185.
17. Glass TA, McAtee MJ: **Behavioral science at the crossroads in public health: extending horizons, envisioning the future**. *Social science & medicine* 2006, **62**(7):1650-1671.
18. Agyemang C: **Rural and urban differences in blood pressure and hypertension in Ghana, West Africa**. *Public Health* 2006, **120**.
19. Agyemang C, Redekop WK, Owusu-Dabo E, Bruijnzeels MA: **Blood pressure patterns in rural, semi-urban and urban children in the Ashanti region on Ghana, West Africa**. *BMC Public Health* 2005, **5**.
20. Kagura J, Adair LS, Musa MG, Pettifor JM, Norris SA: **Blood pressure tracking in urban black South African children: birth to twenty cohort**. *BMC pediatrics* 2015, **15**(1):1.
21. Goon D, Amusa L, Mhlongo D, Khoza L, Any-Anwu F: **Elevated blood pressure among Rural South African Children in Thohoyandou, South Africa**. *Iran J Pub Health* 2013, **42**.
22. Kemp C, Pienaar AE, Schutte AE: **The prevalence of hypertension and the relationship with body composition in Grade 1 learners in the North West Province of South Africa**. *South African Journal of Sports Medicine* 2011, **23**(4).
23. Monyeki K, Kemper H, Makgae P: **The association of fat patterning with blood pressure in rural South African children: the Ellisras Longitudinal Growth and Health Study**. *International journal of epidemiology* 2006, **35**(1):114-120.
24. Schutte A, Van Rooyen J, Huisman H, Kruger H, Malan N, De Ridder J: **Dietary risk markers that contribute to the aetiology of hypertension in black South African children: the THUSA BANA study**. *Journal of human hypertension* 2003, **17**(1):29-35.
25. Cois A, Ehrlich R: **Analysing the socioeconomic determinants of hypertension in South Africa: a structural equation modelling approach**. *BMC public health* 2014, **14**(1):414.
26. Kagura J, Adair LS, Pisa PT, Griffiths PL, Pettifor JM, Norris SA: **Association of socioeconomic status change between infancy and adolescence, and blood pressure, in South African young adults: Birth to Twenty Cohort**. *BMJ open* 2016, **6**(3):e008805.
27. Wilson DK, Kliewer W, Plybon L, Sica DA: **Socioeconomic status and blood pressure reactivity in healthy black adolescents**. *Hypertension* 2000, **35**(1):496-500.
28. Brummett BH, Babyak MA, Siegler IC, Shanahan M, Harris KM, Elder GH, Williams RB: **Systolic Blood Pressure, Socioeconomic Status, and Biobehavioral Risk Factors in a Nationally Representative US Young Adult Sample**. *Hypertension* 2011, **58**(2):161-166.

For peer review only - http://bmjopen.bmj.com/site/about/guidelines.xhtml

29. Chaix B, Bean K, Leal C, Thomas F, Havard S, Evans D, Jégo B, Pannier B: **Individual/Neighborhood Social Factors and Blood Pressure in the RECORD Cohort Study**. *Which Risk Factors Explain the Associations?* 2010, **55**(3):769-775.

30. Griffiths PL, Sheppard ZA, Johnson W, Cameron N, Pettifor JM, Norris SA: **Associations between household and neighbourhood socioeconomic status and systolic blood pressure among urban South African adolescents**. *Journal of biosocial science* 2012, **44**(04):433-458.

31. Longo-Mbenza B, Luila EL, M'Buyamba-Kabangu J: **Nutritional status, socio-economic status, heart rate, and blood pressure in African school children and adolescents**. *International journal of cardiology* 2007, **121**(2):171-177.

32. Aounallah-Skhiri H, El Ati J, Traissac P, Romdhane HB, Eymard-Duvernay S, Delpeuch F, Achour N, Maire B: **Blood pressure and associated factors in a North African adolescent population. A national cross-sectional study in Tunisia**. *BMC Public Health* 2012, **12**(1):1.

33. Afrifa–Anane E, Agyemang C, Codjoe SNA, Ogedegbe G, Aikins Ad-G: **The association of physical activity, body mass index and the blood pressure levels among urban poor youth in Accra, Ghana**. *BMC public health* 2015, **15**(1):269.

34. Anane AE, Agyemang C, Codjoe SNA, Ogedegbe G, Aikins A: **The association of physical activity, Body mass index and blood pressure among urban poor youth in Accra, Ghana**. *BMC Public Health* 2015, **15**.

35. Hu G, Barengo NC, Tuomilehto J, Lakka TA, Nissinen A, Jousilahti P: **Relationship of physical activity and body mass index to the risk of hypertension: a prospective study in Finland**. *Hypertension* 2004, **43**(1):25-30.

36. Juraschek SP, Blaha MJ, Whelton SP, Blumenthal R, Jones SR, Keteyian SJ, Schairer J, Brawner CA, Al-Mallah MH: **Physical fitness and hypertension in a population at risk for cardiovascular disease: the Henry Ford ExercIse Testing (FIT) Project**. *Journal of the American Heart Association* 2014, **3**(6):e001268.

37. Väistö J, Eloranta A-M, Viitasalo A, Tompuri T, Lintu N, Karjalainen P, Lampinen E-K, Ågren J, Laaksonen DE, Lakka H-M: **Physical activity and sedentary behaviour in relation to cardiometabolic risk in children: cross-sectional findings from the Physical Activity and Nutrition in Children (PANIC) Study**. *International Journal of Behavioral Nutrition and Physical Activity* 2014, **11**(1):55.

38. Munthali RJ, Kagura J, Lombard Z, Norris SA: **Childhood adiposity trajectories are associated with late adolescent blood pressure: birth to twenty cohort**. *BMC Public Health* 2016, **16**(1):1-10.

39. STATSSA: **Mortality and causes of death in South Africa, 2015: Findings from death notification**. In. Pretoria: Statistics South Africa; 2017 1–140. .

40. Peer N, Steyn K, Lombard C, Gwebushe N, Levitt N: **A high burden of hypertension in the urban black population of Cape Town: The Cardiovascular Risk in Black South Africans (CRIBSA) Study**. *PLoS One* 2013, **8**(11):e78567.

41. Pisa P, Behanan R, Vorster H, Kruger A: **Social drift of cardiovascular disease risk factors in Africans from the North West Province of South Africa: the PURE study: cardiovascular topics**. *Cardiovascular journal of Africa* 2012, **23**(7):371-388.

42. Steyn K, Fourie J, Lombard C, Katzenellenbogen J, Bourne L, Jooste P: **Hypertension in the black community of the Cape Peninsula, South Africa**. *East African medical journal* 1996, **73**(11):758-763.

43. Tibazarwa K, Ntyintyane L, Sliwa K, Gerntholtz T, Carrington M, Wilkinson D, Stewart S: **A time bomb of cardiovascular risk factors in South Africa: results from the Heart of Soweto Study "Heart Awareness Days"**. *International journal of cardiology* 2009, **132**(2):233-239.

For peer review only - http://bmjopen.bmj.com/site/about/guidelines.xhtml

44. Kahn K, Collinson MA, Gómez-Olivé FX, Mokoena O, Twine R, Mee P, Afolabi SA, Clark BD, Kabudula CW, Khosa A: **Profile: Agincourt health and socio-demographic surveillance system**. *International journal of epidemiology* 2012, **41**(4):988-1001.
45. Pradeilles R, Rousham EK, Norris SA, Griffiths PL: **Urban South African adolescents' perceptions of their neighborhood socio-economic environments: the Birth to Twenty plus cohort study**. *Children Youth and Environments* 2014, **24**(3):173-200.
46. Richter L, Norris S, Pettifor J, Yach D, Cameron N: **Cohort profile: Mandela's children: the 1990 Birth to Twenty study in South Africa**. *International journal of epidemiology* 2007, **36**(3):504-511.
47. Chobanian AV: **Joint National Committee on Prevention, Detection, Evaluation, and Treatment of High Blood Pressure. National Heart, Lung, and Blood Institute; National High Blood Pressure Education Program Coordinating Committee: Seventh report of the Joint National Committee on Prevention, Detection, Evaluation, and Treatment of High Blood Pressure**. *Hypertension* 2003, **42**:1206-1252.
48. Strandberg TE, Pitkala K: **What is the most important component of blood pressure: systolic, diastolic or pulse pressure?** *Current opinion in nephrology and hypertension* 2003, **12**(3):293-297.
49. Cameron N: **The measurement of human growth**: Taylor & Francis; 1984.
50. Lohman TG, Roche AF, Martorell R: **Anthropometric standardization reference manual**: Human kinetics books; 1988.
51. Houweling TA, Kunst AE, Mackenbach JP: **Measuring health inequality among children in developing countries: does the choice of the indicator of economic status matter?** *International journal for equity in health* 2003, **2**(1):8.
52. Feeley AB, Musenge E, Pettifor JM, Norris SA: **Investigation into longitudinal dietary behaviours and household socio-economic indicators and their association with BMI Z-score and fat mass in South African adolescents: the Birth to Twenty (Bt20) cohort**. *Public health nutrition* 2013, **16**(04):693-703.
53. Jones LL, Griffiths PL, Adair LS, Norris SA, Richter LM, Cameron N: **A comparison of the socio-economic determinants of growth retardation in South African and Filipino infants**. *Public health nutrition* 2008, **11**(12):1220-1228.
54. Bull FC, Maslin TS, Armstrong T: **Global physical activity questionnaire (GPAQ): nine country reliability and validity study**. *Journal of Physical Activity and health* 2009, **6**(6):790-804.
55. VanderWeele TJ: **Mediation analysis: a practitioner's guide**. *Annual review of public health* 2016, **37**:17-32.
56. Hu Lt, Bentler PM: **Cutoff criteria for fit indexes in covariance structure analysis: Conventional criteria versus new alternatives**. *Structural equation modeling: a multidisciplinary journal* 1999, **6**(1):1-55.
57. Schermelleh-Engel K, Moosbrugger H, Müller H: **Evaluating the fit of structural equation models: Tests of significance and descriptive goodness-of-fit measures**. *Methods of psychological research online* 2003, **8**(2):23-74.
58. Vandenberg RJ: **Introduction: Statistical and Methodological Myths and Urban Legends: Where, Pray Tell, Did They Get This Idea?** In.: Sage Publications Sage CA: Thousand Oaks, CA; 2006.
59. Fan X, Sivo SA: **Sensitivity of fit indexes to misspecified structural or measurement model components: Rationale of two-index strategy revisited**. *Structural Equation Modeling* 2005, **12**(3):343-367.
60. Alwin DF, Hauser RM: **The decomposition of effects in path analysis**. *American sociological review* 1975:37-47.

For peer review only

61. Williams R, Allison PD, Moral-Benito E: **Xtdpdml: Linear dynamic panel-data estimation using maximum likelihood and structural equation modeling**. In.; 2016.
62. Kerry SM, Emmett L, Micah FB, Martin-Peprah R, Antwi S, Phillips RO, Plange-Rhule J, Eastwood JB, Cappuccio FP: **Rural and semi-urban differences in salt intake, and its dietary sources, in Ashanti, West Africa**. *Ethnicity & disease* 2005, **15**(1):33-39.
63. Lissock CNAA, Sobngwi E, Ngassam E, Ngoa LSE: **Rural and urban differences in metabolic profiles in a Cameroonian population**. *Pan African Medical Journal* 2011, **10**.
64. Stringhini S, Forrester TE, Plange-Rhule J, Lambert EV, Viswanathan B, Riesen W, Korte W, Levitt N, Tong L, Dugas LR: **The social patterning of risk factors for noncommunicable diseases in five countries: evidence from the modeling the epidemiologic transition study (METS)**. *BMC Public Health* 2016, **16**(1):956.
65. de Hoog ML, van Eijsden M, Stronks K, Gemke RJ, Vrijkotte TG: **Association between body size and blood pressure in children from different ethnic origins**. *Cardiovascular diabetology* 2012, **11**(1):136.
66. Dinsa G, Goryakin Y, Fumagalli E, Suhrcke M: **Obesity and socioeconomic status in developing countries: a systematic review**. *Obesity reviews* 2012, **13**(11):1067-1079.
67. Masala G, Bendinelli B, Versari D, Saieva C, Ceroti M, Santagiuliana F, Caini S, Salvini S, Sera F, Taddei S: **Anthropometric and dietary determinants of blood pressure in over 7000 Mediterranean women: the European Prospective Investigation into Cancer and Nutrition-Florence cohort**. *Journal of hypertension* 2008, **26**(11):2112-2120.
68. Wilson BL, Albright GL, Steiner SS, Andreassi JL: **Cardiodynamic response to psychological and cold pressor stress: further evidence for stimulus response specificity and directional fractionation**. *Biofeedback and Self-regulation* 1991, **16**(1):45-53.
69. Goon D, Amusa L, Mhlongo D, Khoza L, Any-Anwu F: **Elevated blood pressure among rural South African children in Thohoyandou, South Africa**. *Iranian journal of public health* 2013, **42**(5):489.
70. Moselakgomo VK, Toriola AL, Shaw BS, Goon DT, Akinyemi O: **Body mass index, overweight, and blood pressure among adolescent schoolchildren in Limpopo province, South Africa**. *Revista Paulista de Pediatria* 2012, **30**(4):562-569.
71. Lynch JW, Smith GD, Kaplan GA, House JS: **Income inequality and mortality: importance to health of individual income, psychosocial environment, or material conditions**. *BMJ: British Medical Journal* 2000, **320**(7243):1200.
72. Ploubidis GB, Mathenge W, De Stavola B, Grundy E, Foster A, Kuper H: **Socioeconomic position and later life prevalence of hypertension, diabetes and visual impairment in Nakuru, Kenya**. *International journal of public health* 2013, **58**(1):133-141.
73. Drewnowski A: **The cost of US foods as related to their nutritive value**. *The American journal of clinical nutrition* 2010, **92**(5):1181-1188.
74. Popkin BM: **The nutrition transition and obesity in the developing world**. *The Journal of nutrition* 2001, **131**(3):871S-873S.
75. Sawaya AL, Sesso R, Florencio TM, Fernandes MT, Martins PA: **Association between chronic undernutrition and hypertension**. *Maternal & child nutrition* 2005, **1**(3):155-163.

For peer review only - http://bmjopen.bmj.com/site/about/guidelines.xhtml

For peer review only

Table 2: Descriptive characteristics

	Total	n	Urban	n	Rural	p value
Age (years)	22.04 (1.24)	492	22.77 (0.49)	476	21.28 (1.31)	0.001
Weight (kg)	64.62 (14.82)	492	64.67 (15.6)	473	64.55 (14.03)	0.90
Height (m)	1.61 (0.007)	492	1.60 (0.07)	475	1.61 (0.07)	0.001
BMI (kg/m^2)	25.05 (5.59)	492	25.32 (5.91)	476	24.78 (5.24)	0.13
BMI classification (%)		492		476		0.015
Underweight (<18.4 kg/m^2)	5.98		7.10		4.82	
Normal weight (18.5-24.9 kg/m^2)	51.34		46.45		56.39	
Overweight (25-29.9 kg/m^2)	26.19		29.21		23.06	
Obese (>=30 kg/m^2)	16.49		17.24		15.72	
Waist circumference (cm)	80.60 (12.08)	492	80.18 (12.63)	476	81.03 (11.47)	0.26
Central obesity, WC ≥ 80 cm , %	43.81	492	45.70	476	44.74	0.55
Household SES index (sum of assets)	7.24 (2.70)	492	8.83 (2.37)	476	5.59 (1.91)	<0.001
Total MVPA (min/week)*	870(280-1810)	492	420(160-900)	385	1680(970-2580)	<0.001
Sitting time (mins/day)*	300 (240-480)	492	360 (240-480)	385	300 (180-360)	<0.001
Systolic blood pressure	106.68 (11.64)	492	110.30 (11.4)	471	102.89 (10.7)	<0.001
Diastolic blood pressure	70.23 (9.00)	492	72.78 (8.3)	471	67.57 (9.0)	<0.001
BP classification (%)		492		471		<0.001
Low BP	12.46		5.49		19.75	
Normal BP	69.16		67.48		70.91	

Prehypertension	16.20		23.58		8.49	
Hypertensive	2.18		3.46		0.85	
Elevated BP (%)	18.38		27.04		9.34	<0.001
Highest Education attained (%)		480		371		<0.001
Primary school	1.18		0		2.70	
Secondary school	60.75		48.33		76.81	
Tertiary education	38.07		51.67		20.49	

Data presented as mean (SD) otherwise stated; ⁎ Median (IQR)

For peer review only

Table 3a: Structural equation model for SES, MVPA and BMI on SBP in urban women

Effect of: N=489	Outcome:	Direct effects(95% CI)	Indirect effects(95% CI)	Total effects(95% CI)	Proportion of total effect mediated
**Household assets**	SBP<br>via BMI	-0.34 (-0.75; 0.07)	0.05 (-0.05; 0.14)	-0.29 (-0.70; 0.12)	0.13 [a]
	BMI<br>via MVPA	0.13 (-0.09; 0.35)	-0.014 (-0.05; 0.013)	0.11 (-0.11; 0.33)	0.1 [a]
	MVPA	-41.71 (-73.48; -9.94)**		-41.71 (-73.48; -9.94)**	
**MVPA**	SBP<br>via BMI	-0.0002 (-0.001; 0.001)	0.0001 (-0.0001; 0.0004)	-0.0000 (-0.0012; 0.0011)	0.3 [a]
**BMI**	SBP	0.37 (0.21; 0.53)***		0.37 (0.21; 0.53)***	

Adjusted for age; * P<0.05; ** P< 0.01; ***P< 0.001; SBP; systolic blood pressure, MVPA; moderate to vigorous intensity physical activity, BMI; body mass index, SES; social economic status, [a] Assessed using the absolute values for both indirect and direct effects

Urban Fit Indices: LR test of model vs. saturated: chi2(4) = 0.97, Prob > chi2 = 0.91; RMSEA = 0.00; **CFI= 1.00** Comparative

For peer review only - http://bmjopen.bmj.com/site/about/guidelines.xhtml

fit index;

TLI= 1.12 Tucker-Lewis index; **SRMR=0.011:** Standardized root mean squared residual, CD= 0.017 Coefficient of determination.

For peer review only

Table 3b: Structural equation model for SES, MVPA and BMI on SBP in rural women

Effect of: N= 378	Outcome:	Direct effects(95% CI)	Indirect effects(95% CI)	Total effects(95% CI)	Proportion of total effect mediated
**Household assets**	SBP	-0.65 (-1.19; -0.096)*		-0.56 (-1.12; -0.02)*	0.11 [a]
	via BMI		0.08 (-0.04; 0.19)		
	BMI	0.27 (0.01; 0.53)*		0.26 (-0.005; 0.53)*	0.04
	via MVPA		-0.01 (-0.04; 0.01)		
	MVPA	-29.51 (-87.81; 28.78)		-29.51 (-87.81; 28.78)	
**MVPA**	SBP	0.0004 (-.0005729 .0013)		0.0005 (-0.0005; 0.0015)	0.2
	via BMI		0.0001 (-0.0000; 0.0003)		
**BMI**	SBP	0.33 (0.12; 0.54)**		0.33 (0.12; 0.54)**	

Adjusted for age; * P<0.05; ** P< 0.01; ***P< 0.001; SBP; systolic blood pressure, MVPA; moderate to vigorous physical activity, BMI; body mass index, SES; social economic status, [a] Assessed using the absolute values for both indirect and direct effects

For peer review only - http://bmjopen.bmj.com/site/about/guidelines.xhtml

Rural Fit Indices: LR test of model vs. saturated: chi2 (4) = 10.51, Prob > chi2 = 0.03; RMSEA = 0.066**; CFI= 0.72** Comparative fit index;

TLI= 0.37 Tucker-Lewis index; **SRMR= 0.04:** Standardized root mean squared residual, CD= 0.03 Coefficient of determination.

For peer review only

Table 3c: Structural equation model for SES, MVPA and BMI on SBP in the pooled sample of urban and rural women

**Effect of: N=867**	**Outcome:**	**Direct effects(95% CI)**	**Indirect effects(95% CI)**	**Total effects(95% CI)**	**Proportion of total effect mediated**
**Household assets**	SBP via BMI	0.23 (-0.08; 0.54)	0.23 (0.10; 0.35)***	0.46 (0.15; 0.76)**	0.5
	BMI via MVPA	0.20 (0.05; 0.34)**	-0.05 (-.100; 0.003)	0.15 (0.01; 0.29)*	0.25 [a]
	MVPA	-144.83 (-170.55; -119.12)***		-144.83 (-170.55; -119.12)***	
**MVPA**	SBP via BMI	-0.001 (-0.002; -0.0005)**	0.0001 (-0.0000; 0.0002)	-0.001 (-0.002; -0.0003)**	0.1 [a]
**BMI**	SBP	0.35 (0.21; 0.49)***		0.35 (0.21; 0.49)***	

Adjusted for age; * P<0.05; ** P< 0.01; ***P< 0.001; SBP; systolic blood pressure, MVPA; moderate to vigorous intensity physical activity, BMI; body mass index, SES; social economic status, [a] Assessed using the absolute values for both indirect and direct effects

For peer review only

Pooled Fit Indices: LR test of model vs. saturated: chi2 (4) = 24.829, Prob > chi2 = 0.000; RMSEA = 0.077**; CFI= 0.89** Comparative fit index; TLI= 0.75 Tucker-Lewis index; **SRMR=0.033:** Standardized root mean squared residual, CD=0.137 Coefficient of determination.

For peer review only

Figure legends

Figure 1: Selection of study participants in rural and urban

Figure 2: Structural equation model for SES, MVPA and BMI on SBP in urban

Figure 3: Structural equation model for SES, MVPA and BMI on SBP in rural

Figure 4: Structural equation model for SES, MVPA and BMI on SBP pooled

For peer review only - http://bmjopen.bmj.com/site/about/guidelines.xhtml

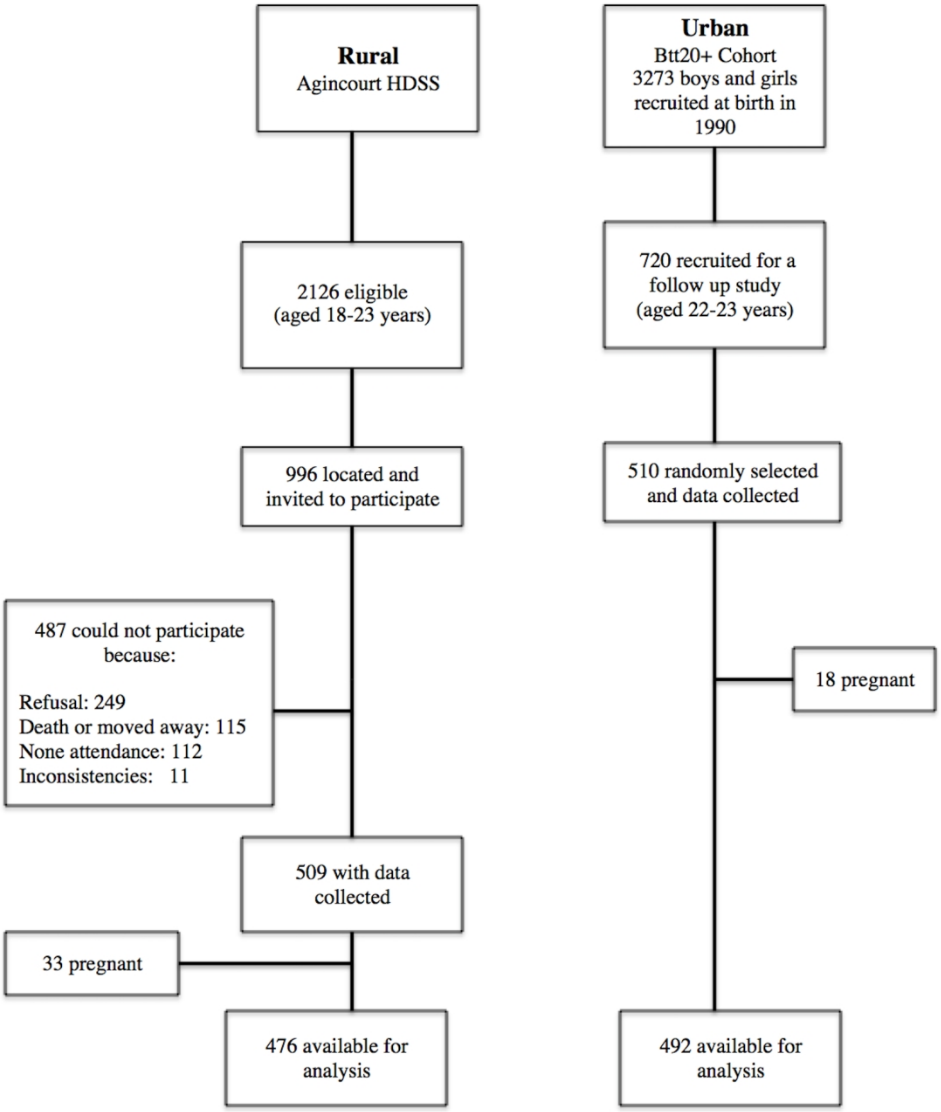

Figure 1: Selection of study participants in rural and urban

144x167mm (300 x 300 DPI)

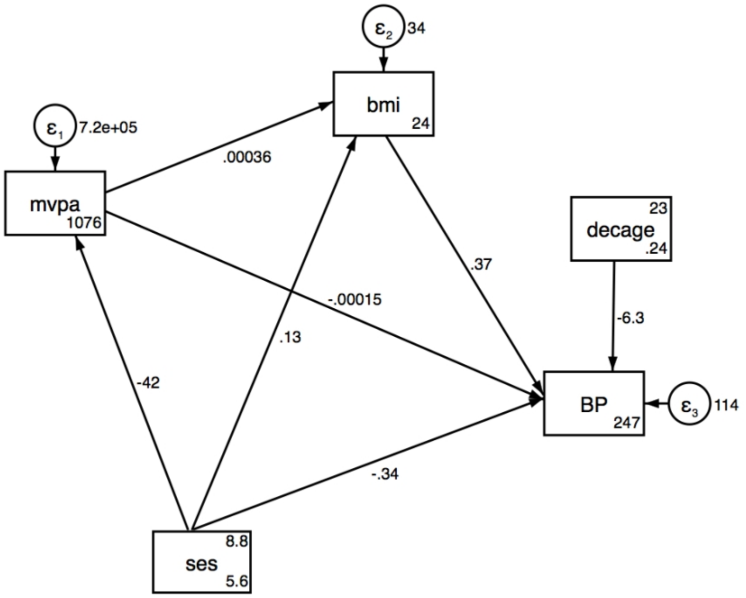

Figure 2: Structural equation model for SES, MVPA and BMI on SBP in urban

106x70mm (300 x 300 DPI)

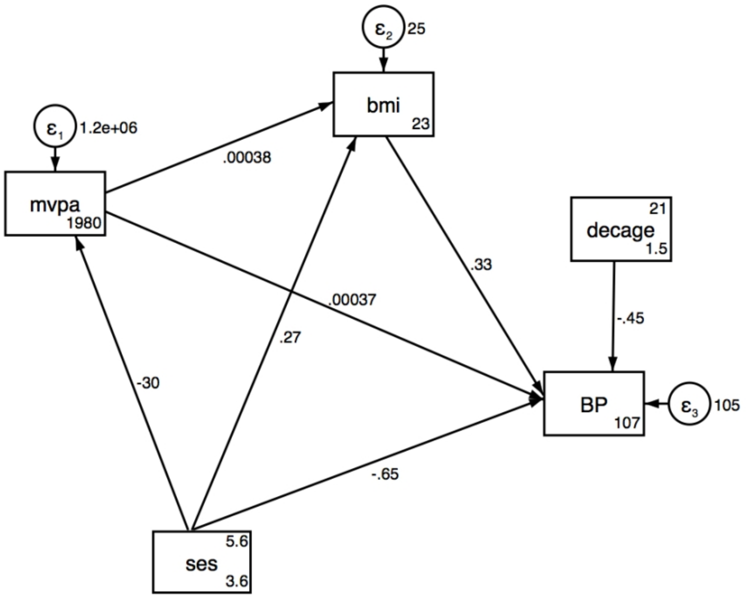

Figure 3: Structural equation model for SES, MVPA and BMI on SBP in rural

106x70mm (300 x 300 DPI)

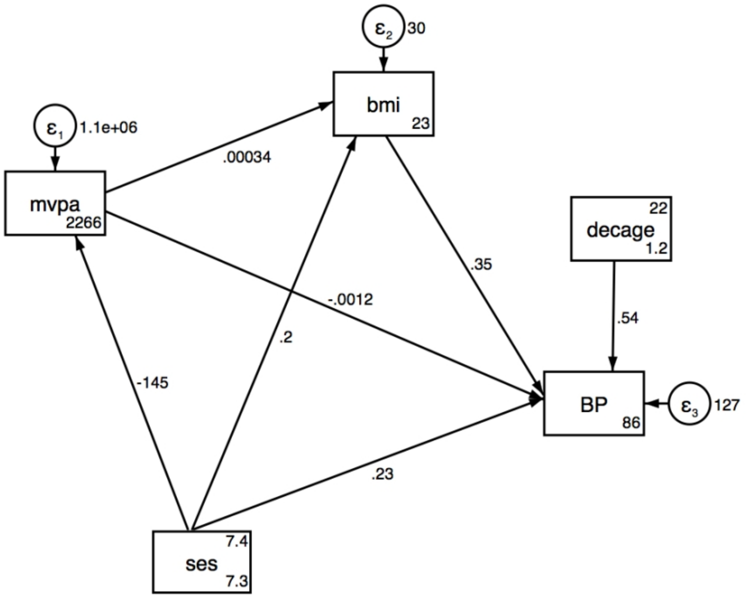

Figure 4: Structural equation model for SES, MVPA and BMI on SBP pooled

106x70mm (300 x 300 DPI)

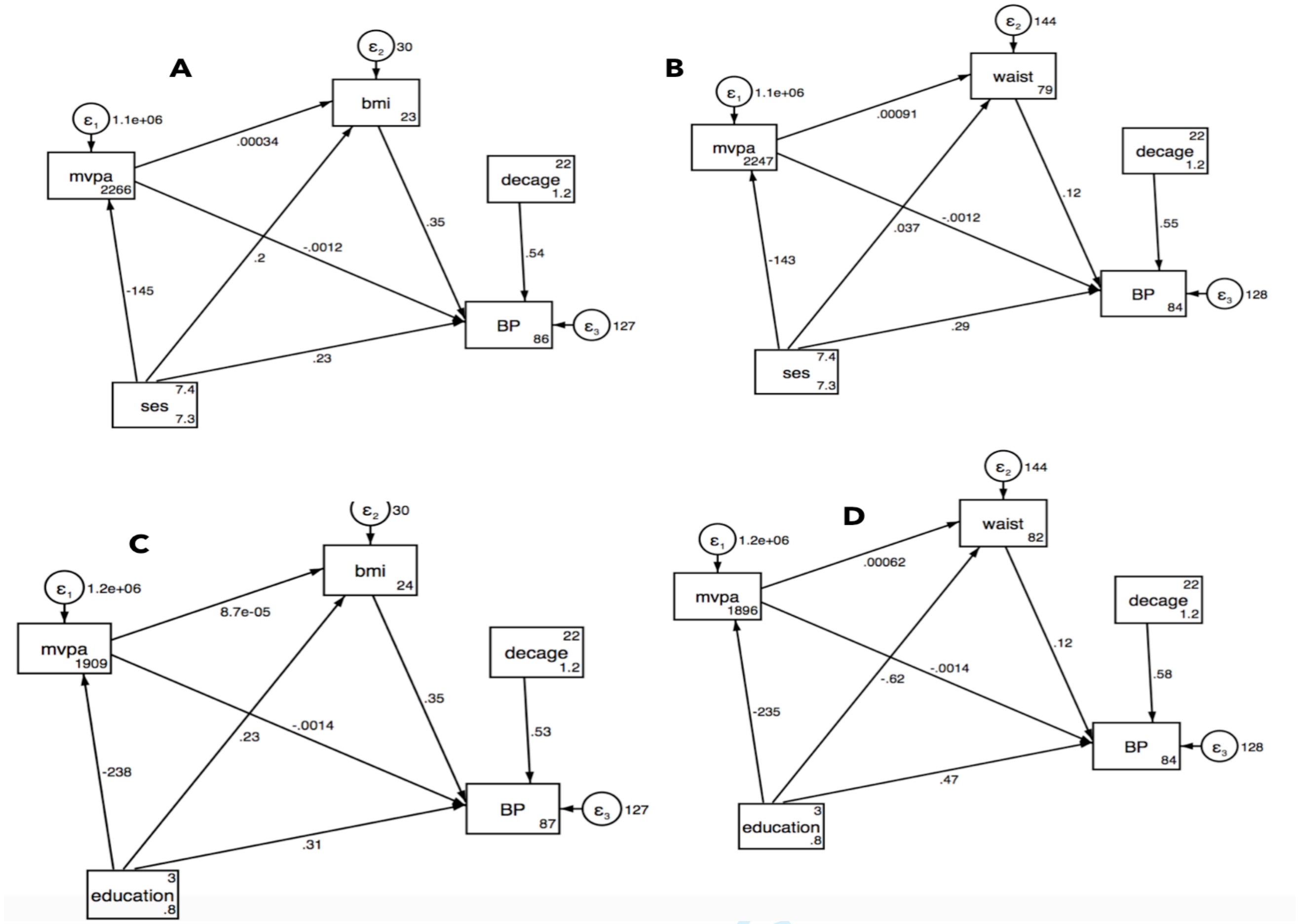

Figure SI; A: Structural equation model for **SES**, MVPA and **BMI** on SBP pooled; B: Structural equation model for **SES**, MVPA and **WC** on SBP pooled; C: Structural equation model for **education,** MVPA and **BMI** on SBP pooled; D: Structural equation model for **education,** MVPA and **WC** on SBP pooled.

For peer review only - http://bmjopen.bmj.com/site/about/guidelines.xhtml

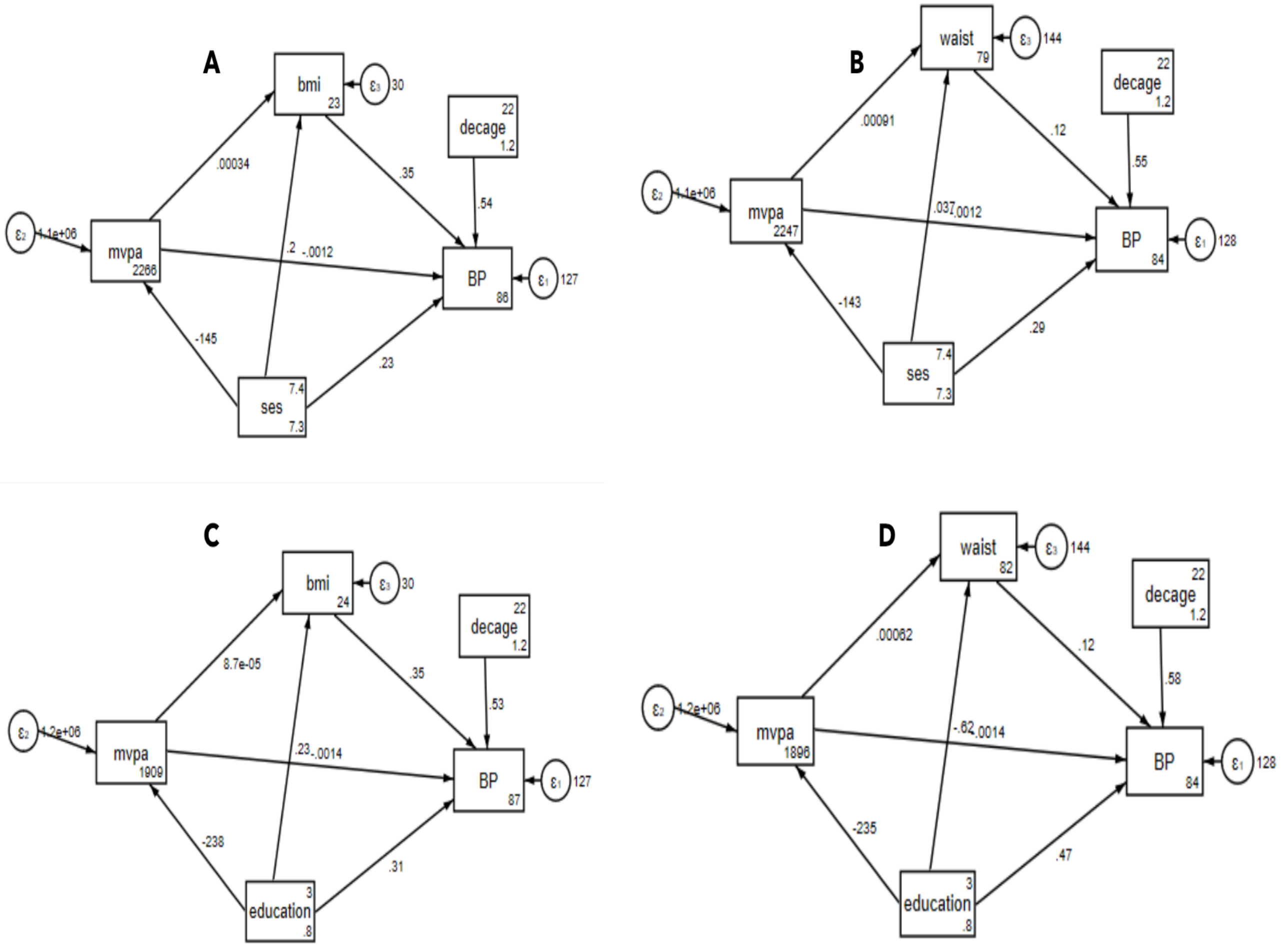

Figure S2 - SEMs based on Satorra–Bentler; A: Structural equation model for **SES**, MVPA and **BMI** on SBP pooled; B: Structural equation model for **SES**, MVPA and **WC** on SBP pooled; C: Structural equation model for **education,** MVPA and **BMI** on SBP pooled; D: Structural equation model for **education,** MVPA and **WC** on SBP pooled.

STROBE Statement—checklist of items that should be included in reports of observational studies

	Item No	Recommendation
**Title and abstract**	1	(*a*) Indicate the study's design with a commonly used term in the title or the abstract **MS Page 4 Par 2**
		(*b*) Provide in the abstract an informative and balanced summary of what was done and what was found **MS Page 4**
**Introduction**		
Background/rationale	2	Explain the scientific background and rationale for the investigation being reported **MS Pages 5-6**
Objectives	3	State specific objectives, including any prespecified hypotheses **MS Page 6 Par 2**
**Methods**		
Study design	4	Present key elements of study design early in the paper **Methods: MS Page 6 Par 3**
Setting	5	Describe the setting, locations, and relevant dates, including periods of recruitment, exposure, follow-up, and data collection **Methods: MS Page 6 Par 3**
Participants	6	(*a*) *Cohort study*—Give the eligibility criteria, and the sources and methods of selection of participants. Describe methods of follow-up **N/A**<br><br>*Case-control study*—Give the eligibility criteria, and the sources and methods of case ascertainment and control selection. Give the rationale for the choice of cases and controls **N/A**<br>*Cross-sectional study*—Give the eligibility criteria, and the sources and methods of selection of participants **Methods: MS Page 6 Par 3**
		(*b*) *Cohort study*—For matched studies, give matching criteria and number of exposed and unexposed **N/A**<br>*Case-control study*—For matched studies, give matching criteria and the number of controls per case **N/A**
Variables	7	Clearly define all outcomes, exposures, predictors, potential confounders, and effect modifiers. Give diagnostic criteria, if applicable **Methods: MS Page 6 Par 4 – Page 8 Par 1**
Data sources/ measurement	8*	For each variable of interest, give sources of data and details of methods of assessment (measurement). Describe comparability of assessment methods if there is more than one group **Methods: MS Page 6 Par 4 – Page 8 Par 1**
Bias	9	Describe any efforts to address potential sources of bias **Methods: MS Page 6 Par 3**
Study size	10	Explain how the study size was arrived at **Methods: MS Page 6 Par 3**
Quantitative variables	11	Explain how quantitative variables were handled in the analyses. If applicable, describe which groupings were chosen and why **Methods: MS Page 8 Par 2**
Statistical methods	12	(*a*) Describe all statistical methods, including those used to control for confounding **Statistical analyses: MS Page 8 Par 2 and 3**
		(*b*) Describe any methods used to examine subgroups and interactions **N/A**
		(*c*) Explain how missing data were addressed **Statistical analyses: MS Page 8 Par 2**
		(*d*) *Cohort study*—If applicable, explain how loss to follow-up was addressed<br>*Case-control study*—If applicable, explain how matching of cases and controls was

For peer review only - http://bmjopen.bmj.com/site/about/guidelines.xhtml

	addressed **N/A**<br>*Cross-sectional study*—If applicable, describe analytical methods taking account of sampling strategy **N/A**
	(*e*) Describe any sensitivity analyses **N/A**

Continued on next page

For peer review only

For peer review only - http://bmjopen.bmj.com/site/about/guidelines.xhtml

**Results**		
Participants	13*	(a) Report numbers of individuals at each stage of study—eg numbers potentially eligible, examined for eligibility, confirmed eligible, included in the study, completing follow-up, and analysed **Results: Page 8 Par 5 – Page 9 Par 1**
		(b) Give reasons for non-participation at each stage **N/A**
		(c) Consider use of a flow diagram **N/A**
Descriptive data	14*	(a) Give characteristics of study participants (eg demographic, clinical, social) and information on exposures and potential confounders **Page 8 Par 5 – Page 9 Par 1**
		(b) Indicate number of participants with missing data for each variable of interest **Results: Page 8 Par 5**
		(c) *Cohort study*—Summarise follow-up time (eg, average and total amount**) N/A**
Outcome data	15*	*Cohort study*—Report numbers of outcome events or summary measures over time **N/A**
		Case-control study—Report numbers in each exposure category, or summary measures of exposure **N/A**
		Cross-sectional study—Report numbers of outcome events or summary measures **Results: Page 8 Par 5**
Main results	16	(*a*) Give unadjusted estimates and, if applicable, confounder-adjusted estimates and their precision (eg, 95% confidence interval). Make clear which confounders were adjusted for and why they were included **Results: Page 9 Par 1**
		(*b*) Report category boundaries when continuous variables were categorized **N/A**
		(*c*) If relevant, consider translating estimates of relative risk into absolute risk for a meaningful time period **N/A**
Other analyses	17	Report other analyses done—eg analyses of subgroups and interactions, and sensitivity analyses **N/A**
**Discussion**		
Key results	18	Summarise key results with reference to study objectives **Discussion: Page 9 Par 2**
Limitations	19	Discuss limitations of the study, taking into account sources of potential bias or imprecision. Discuss both direction and magnitude of any potential bias **Discussion: Page 11 Par 4**
Interpretation	20	Give a cautious overall interpretation of results considering objectives, limitations, multiplicity of analyses, results from similar studies, and other relevant evidence **Page 9 Par 3 – Page 11 Par 3**
Generalisability	21	Discuss the generalisability (external validity) of the study results **Discussion: Page 9 Par 3 – Page 11**
**Other information**		
Funding	22	Give the source of funding and the role of the funders for the present study and, if applicable, for the original study on which the present article is based **Funding: Page 12 Par 1**

*Give information separately for cases and controls in case-control studies and, if applicable, for exposed and unexposed groups in cohort and cross-sectional studies.

Note: An Explanation and Elaboration article discusses each checklist item and gives methodological background and published examples of transparent reporting. The STROBE checklist is best used in conjunction with this article (freely available on the Web sites of PLoS Medicine at http://www.plosmedicine.org/, Annals of Internal Medicine at http://www.annals.org/, and Epidemiology at http://www.epidem.com/). Information on the STROBE Initiative is available at www.strobe-statement.org.

For peer review only
